# Parallel Synthesis of a Library of Symmetrically- and Dissymmetrically-disubstituted Imidazole-4,5-dicarboxamides Bearing Amino Acid Esters

**DOI:** 10.3390/molecules14010352

**Published:** 2009-01-13

**Authors:** Rosanna Solinas, John C. DiCesare, Paul W. Baures

**Affiliations:** Department of Chemistry and Biochemistry, The University of Tulsa, 800 South Tucker Drive, Tulsa, OK 74104; E-mails: rosannasolinas@yahoo.it (R. S.), john-dicesare@utulsa.edu (J-C. D.)

**Keywords:** Imidazole, NIH Roadmap, Heterocyclic scaffold, Drug discovery.

## Abstract

The imidazole-4,5-dicarboxylic acid scaffold is readily derivatized with amino acid esters to afford symmetrically- and dissymmetrically-disubstituted imidazole-4,5-dicarboxamides with intramolecularly hydrogen bonded conformations that predispose the presentation of amino acid pharmacophores. In this work, a total of 45 imidazole-4,5-dicarboxamides bearing amino acid esters were prepared by parallel synthesis. The library members were purified by column chromatography on silica gel and the purified compounds characterized by LC-MS with LC detection at 214 nm. A selection of the final compounds was also analyzed by ^1^H-NMR spectroscopy. The analytically pure final products have been submitted to the Molecular Library Small Molecule Repository (MLSMR) for screening in the Molecular Library Screening Center Network (MLSCN) as part of the NIH Roadmap.

## Introduction

Small molecule biological probes that influence protein function are useful tools for studying the cell and can also serve as lead structures in the development of new therapeutic agents [1,2,3,4,5]. The NIH Roadmap [6] project has created opportunities for the development of new chemical libraries for subsequent screening by the Molecular Libraries Screening Center Network (MLSCN) in a combined effort to identify unique and useful biological probes. 

Selecting compounds for screening is an important, albeit challenging, task [7,8]. Ideally, the compounds are pure and of known structure, contain only non-reactive functional groups, have a possibility of some level of selectivity towards a given protein target or at least toward a protein class, and have the physical properties suitable for use in biological assays, including aqueous solubility and cellular permeability. Compounds that meet part or all of these parameters are generally described as “drug-like,” even if their chemical structures can be quite divergent—from marine natural products with multiple stereocenters and many polar functional groups to small molecule achiral synthetic compounds that are quite hydrophobic [9,10]. 

This paper describes the synthesis and characterization of an imidazole-4,5-dicarboxamide (I45DC) library substituted with two α-amino acid esters. We have been using the imidazole-4,5-dicarboxylic acid (I45DA) scaffold to design compounds that inhibit specific biological targets [11,12,13], as well as for the synthesis of chemical libraries to be used in the MLSCN screening effort. Nevertheless, no dissymmerically-disubstituted I45DCs bearing only α-amino acid esters and only two symmetrically-disubstituted I45DCs bearing α-amino acids esters have been previously reported in the literature. There are related bis-I45DCs bearing one α-amino acid ester per I45DC that we used to target a protein-protein interaction [12], as well as dissymmetrically-disubstituted I45DCs bearing one α-amino acid ester along with either a primary or secondary alkanamine that we are concurrently reporting [14]. 

Amino acid side chains are natural recognition elements in substrate-enzyme, ligand-receptor, and protein-protein interactions, and we have shown that I45DCs form a strong intramolecular hydrogen bond that remains stable even in water at pH 7 [15]. Moreover, the strong intramolecular hydrogen bond anticipated in each of these I45DCs accomplishes three important tasks: it predisposes the amino acid ester substituents to be separated by a distance comparable with side chain separations found adjacent on one side in an α-helix or on one side in a β-strand secondary structure [12,16], yields a quasi ring that combines with the imidazole to yield mimics of substituted purines [13,16], and offers added low-energy conformational flexibility for the compound to adapt to the binding site, which is an advantage over an all covalently bonded scaffold.

Our design of this library incorporated two amino acid ester pharmacophores in each I45DC, reasoning that α-amino acids are natural recognition elements in substrate-enzyme, ligand-receptor, and protein-protein interactions. All of the final compounds are of analytical purity and have been submitted to the Molecular Library Small Molecule Repository (MLSMR) for use by the Molecular Library Screening Center Network (MLSCN).

## Results and Discussion

A library for high-throughput screening and hit identification in the discovery phase of identifying small molecule probes and drugs must strike a balance between sufficient numbers of compounds in class to sample pharmacophoric space without a significant redundancy. This balance saves time and energy in both delivering the compounds and performing the bioassay, as well as in the overall costs of the effort. 

The α-amino acid esters used in the synthesis of this library were of the *S*-configuration and are given in Table 1. We opted for a set of mostly hydrophobic side chains in this work, since a less-than-perfect burial of a hydrophobic side chain in a binding cleft would still be expected to show some bioactivity in a screening effort, in contrast with a polar α-amino acid where electrostatic interactions, such as hydrogen bonding, form quite specific interactions geometrically and may not bind at all if those conditions are not met. This stated, the amino acids in this library include Ala, Leu, and Phe, along with Gly. Lysine was also included, but the side chain left protected by a Boc group in this initial library. The carboxylic acid was protected with either a *tert*-butyl or benzyl ester to provide a hydrophobic group for binding interactions, while also providing a convenient deprotection and modification strategy for derivitizing bioactive compounds. 

**Table 1:** Amino acid ester salts, **2**{*1*-*9*}, used in library synthesis.

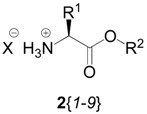			
Member	R^1^	R^2^	X 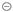
**2**{*1*}	H	C(CH_3_)_3_	Cl
**2**{*2*}	H	CH_2_Ph	Cl
**2**{*3*}	CH_3_	C(CH_3_)_3_	Cl
**2**{*4*}	CH_3_	CH_2_Ph	Cl
**2**{*5*}	CH_2_CH(CH_3_)_2_	C(CH_3_)_3_	Cl
**2**{*6*}	CH_2_CH(CH_3_)_2_	CH_2_Ph	OSO_2_C_6_H_4_CH_3_
**2**{*7*}	CH_2_Ph	C(CH_3_)_3_	Cl
**2**{*8*}	CH_2_Ph	CH_2_Ph	Cl
**2**{*9*}	(CH_2_)_4_NH_2_	C(CH_3_)_3_	Cl

The resulting I45DC library members have reasonable drug-like properties, as illustrated by their molecular weights that range from 382−724 g/mol with an average value of 522 g/mol, cLogP values that range from 0.35−4.83 with an average value of 2.95, and relatively few rotatable bonds [17,18,19].

**Scheme 1:**
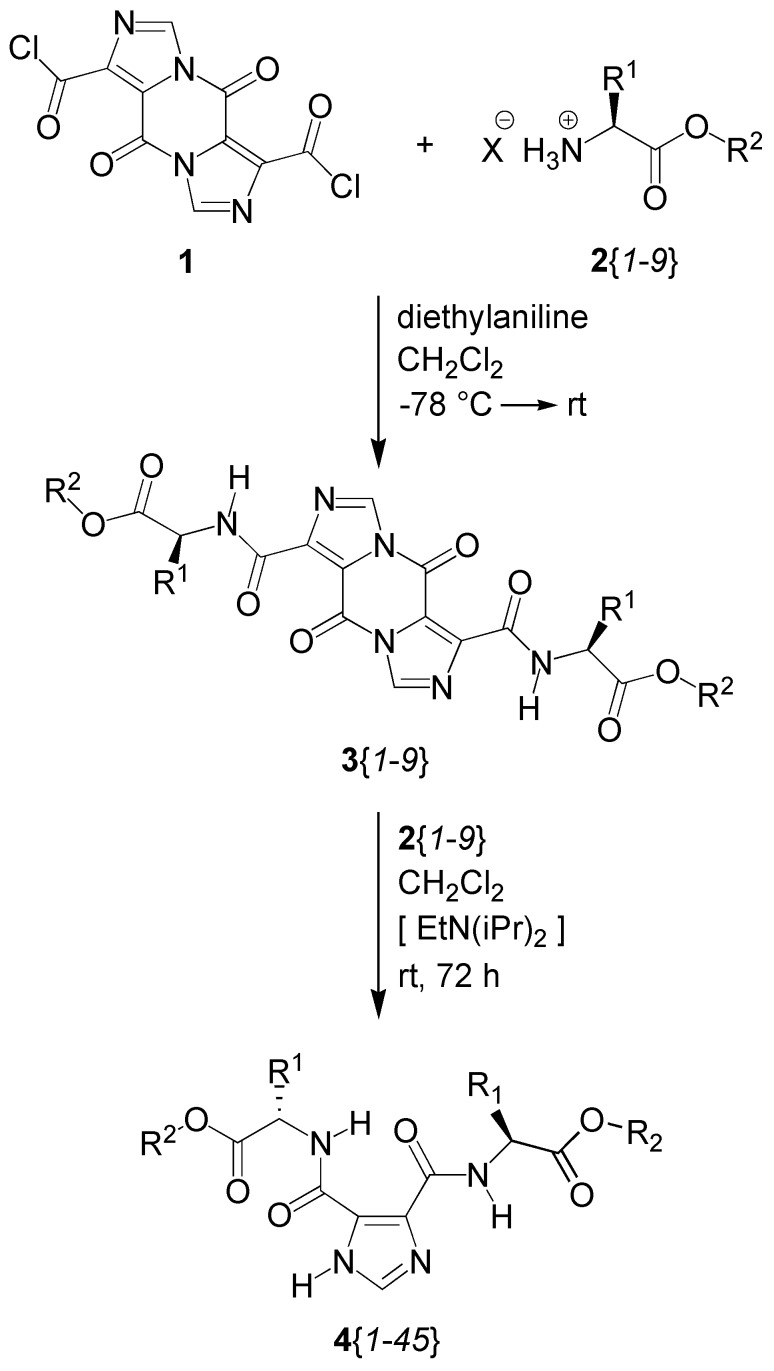
Synthesis of library members.

The synthetic strategy to the pyrazine intermediates and final dI45DCs is shown in Scheme 1. The starting pyrazine diacid chloride, **1**, was prepared as previously described [8], as were the amino acid ester substituted pyrazines, **3** [14]. Reaction of the appropriate pyrazine, **3**, with two equivalents of a second amino acid ester hydrochloride or tosylate salt, **2**{1-9}, and two equivalents of *N*,*N*-diisopropyl-ethylamine as a scavenger for the acid produced a good average yield (73%) of the final I45DC products, **4**, following purification by column chromatography. All of the compounds were analyzed by using LC-MS with LC detection at 214 nm, while selected compounds were analyzed by ^1^H-NMR spectroscopy. The yields to the 9 sI45DCs and 36 dI45DCs are given in Table 2 and Table 3, respectively. A comparative analysis of the low, high, average, and median yields as a function of an α-amino acid ester is given in Table 4 and indicates there is no consequence on reaction yield based on structure. We have included tables of data (formula, molecular weight, cLogP values, physical form of the compound, *R_f_* values, and retention times in the LC-MS) for library members **5{**1-45}, LC-MS spectra for all library members, LC-MS data for 10 reactions to crude library members, as well as ^1^H-NMR spectra for the crude reactions and purified library members for 15 representative compounds in a supporting file. The same information is also available for each compound at the project website [20].

**Table 2:** Symmetrically-disubstituted Amino Acid Ester I45DCs, **4**{*1*-*9*}.

Cmpd.	Reactants		Yield (%)
**4{***1*}	**2**{*1*}	**2**{*1*}	79
**4{***2*}	**2**{*2*}	**2**{*2*}	22
**4{***3*}	**2**{*3*}	**2**{*3*}	69
**4{***4*}	**2**{*4*}	**2**{*4*}	12
**4{***5*}	**2**{*5*}	**2**{*5*}	83
**4{***6*}	**2**{*6*}	**2**{*6*}	97
**4{***7*}	**2**{*7*}	**2**{*7*}	62
**4{***8*}	**2**{*8*}	**2**{*8*}	73
**4{***9*}	**2**{*9*}	**2**{*9*}	86

**Table 3:** Dissymmetrically-disubstituted Amino Acid Ester I45DCs, **4**{*10*-*45*}.

Cmpd.	Reactants		Yield (%)	Cmpd.	Reactants		Yield (%)
**4{***10*}	**2**{*1*}	**2**{*2*}	83	**4{***28*}	**2**{3}	**2**{*7*}	54
**4{***11*}	**2**{*1*}	**2**{*3*}	86	**4{***29*}	**2**{3}	**2**{*8*}	56
**4{**1*2*}	**2**{*1*}	**2**{*4*}	85	**4{***30*}	**2**{3}	**2**{*9*}	77
**4{**1*3*}	**2**{*1*}	**2**{*5*}	91	**4{***31*}	**2**{4}	**2**{*5*}	86
**4{**1*4*}	**2**{*1*}	**2**{*6*}	83	**4{***32*}	**2**{*4*}	**2**{*6*}	97
**4{**1*5*}	**2**{*1*}	**2**{*7*}	78	**4{***33*}	**2**{*4*}	**2**{*7*}	59
**4{**1*6*}	**2**{*1*}	**2**{*8*}	84	**4{***34*}	**2**{*4*}	**2**{*8*}	90
**4{**1*7*}	**2**{*1*}	**2**{*9*}	61	**4{***35*}	**2**{*4*}	**2**{*9*}	63
**4{**1*8*}	**2**{*2*}	**2**{*3*}	56	**4{***36*}	**2**{*5*}	**2**{*6*}	94
**4{**1*9*}	**2**{*2*}	**2**{*4*}	70	**4{***37*}	**2**{*5*}	**2**{*7*}	87
**4{***20*}	**2**{*2*}	**2**{*5*}	69	**4{***38*}	**2**{*5*}	**2**{*8*}	85
**4{***21*}	**2**{*2*}	**2**{*6*}	55	**4{***39*}	**2**{*5*}	**2**{*9*}	75
**4{***22*}	**2**{*2*}	**2**{*7*}	67	**4{***40*}	**2**{*6*}	**2**{*7*}	88
**4{***23*}	**2**{*2*}	**2**{*8*}	64	**4{***41*}	**2**{*6*}	**2**{*8*}	96
**4{***24*}	**2**{*2*}	**2**{*9*}	62	**4{***42*}	**2**{*6*}	**2**{*9*}	95
**4{***25*}	**2**{3}	**2**{*4*}	57	**4{***43*}	**2**{*7*}	**2**{*8*}	48
**4{***26*}	**2**{3}	**2**{*5*}	76	**4{***44*}	**2**{*7*}	**2**{*9*}	59
4{*27*}	**2**{3}	**2**{*6*}	94	**4{***45*}	**2**{*8*}	**2**{*9*}	56

**Table 4:** Percent yields of purified library members based on amino acid esters, **2**{1-9}.

Member	Low	High	Average	Median
**2**{*1*}	61	91	81	83
**2**{*2*}	22	83	61	64
**2**{*3*}	54	94	69	69
**2**{*4*}	12	97	69	70
**2**{*5*}	69	94	83	85
**2**{*6*}	55	97	89	94
**2**{*7*}	48	88	67	62
**2**{*8*}	48	96	72	73
**2**{*9*}	56	95	70	63

A total of 10 reactions were run and the individual crude reactions compared to the corresponding pure compounds in order to estimate the level of purity in the crude library and to provide some indication of the major impurities present. As with the dI45DC libraries bearing amino acid esters and alkanamines [14], the major impurity in this library results from hydrolysis of the pyrazine intermediate, **3**, to give an imidazole-4-substituted carboxamide-5-carboxylic acid. We observed evidence of this impurity by LC-MS in 8 out of 10 of the crude reactions, although the level of its presence appears low compared to the desired product based on the UV trace at 214 nm. There was evidence by LC-MS for this impurity in the other 2 crude reactions, but this ion gave a relatively low number of counts in the MS spectrum and, more significantly, the signal was also under the product peak, so we think the presence of this ion is likely an ionization pathway of the product in these two instances and does not mean there was significant hydrolysis in the reaction. Indeed, one of these reactions provided a 97% purified yield and the other a 79% purified yield, supporting this hypothesis. There is no evidence in the LC-MS traces of any other substantial impurities in the crude reactions, although we know that the diisopropylethylamine hydrochloride (DIEA**.**HCl) is still present. 

The ^1^H-NMR spectra of the crude reactions are similar to those of their purified products, aside from the extra DIEA**.**HCl signals and variations in the amide and imidazole NH signals in the crude reactions as compared with their purified products (see supporting information). The amide and imidazole NH chemical shifts are known to be sensitive to both acidity and solvent [15], and it is hypothesized that the presence of the DIEA**.**HCl is the cause for the difference in the chemical shifts between the crude and purified compounds. 

**Figure 1:**
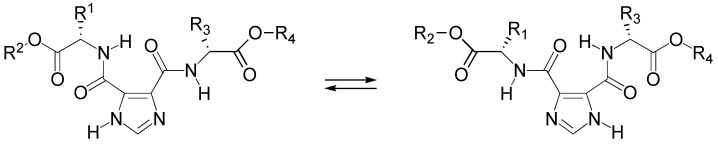
Two intramolecularly hydrogen bonded conformations of the dI45DCs.

These compounds have two intramolecularly hydrogen bonded conformations as illustrated in Figure 1. The percentage of each conformation for these compounds has not been determined, although we have noted in the past that the structure can bias one conformation over another by as much as 80% to 20% [13,21]. One advantage of this intramolecular hydrogen bond is that it yields two conformations of every compound in this library and that either conformation or both may be bioactive. We reason that a compound with significant bioactivity would be discovered in preliminary biological screens even when tested at half of the initial screening concentration, as would be in the case when there are two equivalent hydrogen bonded conformations. Thus, this library provides two possible bioactive structures for each compound. Subsequent structure-activity relationship development can include the synthesis of imidazole-4-ester-5-amide derivatives with chiral α-hydroxyacid esters, incorporating one *N*-methylamino acid ester in the dI45DC, or *N*-alkylating the imidazole ring in order to control the hydrogen bond donor and acceptor and thereby gain insight on the bioactive conformation [16].

The library members have all been submitted to and accepted into the MLSMR for screening by the MLSCN. At the time of this writing, two of the compounds in this library, **4**{9} and **4**{39}, showed bioactivity in initial screens towards calpain II, but each just missed the activity cutoff in the follow up investigation which was greater than 50% inhibition when tested at 0.225 mM [22]. The current biological data for all of these compounds is best accessed through the PubChem database through a structure search [23]. Table 5 provides the compound number in this report with the corresponding PubChem SID numbers that can also be used to find information in PubChem. There is also recently reported software, PubChemSR, that operates in a Windows environment and may be useful in helping researchers mine the data found in PubChem [24].

**Table 5:** Cross reference guide for library members with the PubChem database (SID identification number).

Cmpd.	PubChem SID^‡^	Cmpd.	PubChem SID^‡^
**4{***1*}	26732547	**4{***24*}	49733438
**4{***2*}	49713852	**4{***25*}	49713850
**4{***3*}	26732537	**4{***26*}	26732546
**4{***4*}	49733436	**4{***27*}	26732531
**4{***5*}	26732515	**4{***28*}	26732516
**4{***6*}	49734139	**4{***29*}	49713848
**4{***7*}	26732535	**4{***30*}	26732538
**4{***8*}	49733439	**4{***31*}	49713847
**4{***9*}	26732539	**4{***32*}	50096426
**4{***10*}	49713854	**4{***33*}	49734319
**4{***11*}	26732541	**4{***34*}	49714451
**4{***12*}	49713857	**4{***35*}	49713851
**4{***13*}	26732520	**4{***36*}	49713845
**4{***14*}	26732540	**4{***37*}	26732533
**4{***15*}	26732542	**4{***38*}	49731974
**4{***16*}	49713858	**4{***39*}	26732534
**4{***17*}	26732514	**4{***40*}	49713844
**4{***18*}	49713855	**4{***41*}	49713843
**4{***19*}	49731975	**4{***42*}	49713846
**4{***20*}	49713856	**4{***43*}	49713849
**4{***21*}	49713853	**4{***44*}	26732536
**4{***22*}	49731976	**4{***45*}	50096427
**4{***23*}	49733857		

^‡^The PubChem information for each library member, along with the current bioassay results, can be found by inserting the appropriate SID number into the spaces indicated by ######### in the following link: [http://pubchem.ncbi.nlm.nih.gov/summary/summary.cgi?sid=########&loc =ec_rcs]. Since each submission of a compound results in a unique PubChem SID number, it is useful to search for other samples of the same compound under the link “Related Structures”. Biological data may be listed for one sample of a compound and not for other samples of the same compound.

## Experimental

### General

All apparatus was oven-dried and cooled in a desiccator. All reagents were purchased from commercial suppliers and used without further purification. Reagent grade CH_2_Cl_2_ was distilled from CaH_2_ before use. Thin layer chromatography (TLC) was performed on 250 µm glass-backed silica gel plates and visualized using UV. Column chromatography was performed on silica gel (Merck, grade 9385, 230-400 mesh, 60 Å). The columns were prepared in plastic 20 or 30 mL syringe bodies and filled to approximately 2/3 of their capacity with silica gel. This column volume was sufficient for purification of most reaction products because the most substantial impurity is the imidazole-4,5-dicarboxylic acid substituted with a carboxamide and carboxylic acid that absorbs strongly to silica gel. In select cases, a second column was necessary in order to achieve analytically pure material. ^1^H-NMR spectra were recorded at 10 mM at 400 MHz in CDCl_3_ with CHCl_3_ as the internal reference (δ 7.24). The names of the final compounds are provided in Table 6 at the end of the experimental section.

### LC-MS Analysis

Characterization of the purity and identity of the library members was carried out by liquid chromatography-mass spectrometry (LC-MS) using a Varian 500-MS LC Ion Trap mass spectrometer. Solutions of the compounds were prepared at an approximate concentration of 1 mg/mL by first adding <10% by volume of CH_2_Cl_2_ to dissolve the sample and then diluting to the final volume with HPLC grade methanol. Five microliters of the sample was injected onto a Polaris 5 mm C18-A (50×2.0 mm) HPLC column and eluted with a gradient of CH_3_CN/H_2_O containing 0.1% CH_3_CO_2_H at a 0.2 mL/min flow rate. Compounds were detected at 214 nm. The gradient was as follows: 0 min., 4:6 CH_3_CN/H_2_O → 1 min., 4:6 CH_3_CN/H_2_O → 6 min., 9:1 CH_3_CN/H_2_O → 8 min., 9:1 CH_3_CN/H_2_O → 9 min., 4:6 CH_3_CN/H_2_O → 10 min., 4:6 CH_3_CN/H_2_O. The mass spectrum was recorded for the entire elution time by using ESI detection from 50-800 (*m*/*z*) with the following parameters: capillary voltage at 60.0, R_f_ loading at 100%, drying gas at 250 °C, spray chamber at 50 °C, nebulizer gas at 50.0 psi, drying gas at 25 psi, and damping gas at 0.8 mL/min. 

### Synthesis

*Symmetrically- and Dissymmetrically-disubstituted Imidazole-4,5-dicarboxamides* **[4{1-45}].** Screw capped culture tubes were dried overnight in a drying oven. Each tube was filled with a unique combination of one amino acid ester substituted pyrazine **3**{1-9} (0.100 mmol) and one α-amino acid ester hydrochloride or tosylate salt **2**{1-9} (0.200 mmol) (for a total of 45 tubes). Dry CH_2_Cl_2_ (3 mL) was added to each tube. The tubes were then cooled to 0 °C and *N,N*-diisopropylethylamine (25.8 mg, 34.8 μL, 0.200 mmol) was added. The tubes were purged with argon and sealed. The resulting solutions were shaken for 3 days at room temperature. The progress of each reaction was followed with TLC by using a mixture of EtOAc/hexanes (1:1) as the eluant. The solvent was evaporated and the residues were purified by column chromatography on silica gel using EtOAc/hexanes (1:1) and then just EtOAc as the eluants. The desired fractions were combined and concentrated to give the final products **4**{1-45}. The purity and identity of the final products was determined by LC-MS as well as ^1^H NMR spectroscopy for selected examples.

**Table 6:** Final Product Numbers and Nomenclature.

cmpd	Name
**4{***1*}	4,5-bis[(*tert*-butoxyglycyl)carbonyl]-1*H*-imidazole
**4{***2*}	4,5-bis[(benzyloxyglycyl)carbonyl]-1 *H*-imidazole
**4{***3*}	4,5-bis[(*tert*-butoxy-*S*-alanyl)carbonyl]-1*H*-imidazole
**4{***4*}	4,5-bis[(benzyloxy-*S*-alanyl)carbonyl]-1*H*-imidazole
**4{***5*}	4,5-bis[(*tert*-butoxy-*S*-leucyl)carbonyl]-1*H*-imidazole
**4{***6*}	4,5-bis[(benzyloxy-*S*-leucyl)carbonyl]-1*H*-imidazole
**4{***7*}	4,5-bis[(*tert*-butoxy-*S*-phenylalanyl)carbonyl]-1*H*-imidazole
**4{***8*}	4,5-bis[(benzyloxy-*S*-phenylalanyl)carbonyl]-1*H*-imidazole
**4{***9*}	4,5-bis[(*tert*-butoxy*-S*-[*N*^ε^-(*tert*-butoxy)carbonyl]lysyl)carbonyl]-1*H*-imidazole
**4{***10*}	4-[(benzyloxyglycyl)carbonyl]-5-[( *tert*-butoxyglycyl)carbonyl]-1*H*-imidazole
**4{***11*}	4-[(*tert*-butoxy-*S*-alanyl)carbonyl]-5-[(*tert*-butoxyglycyl)carbonyl]-1*H*-imidazole
**4{***12*}	4-[(benzyloxy-*S*-alanyl)carbonyl]-5-[(*tert*-butoxyglycyl)carbonyl]-1*H*-imidazole
**4{***13*}	4-[(*tert*-butoxy-*S*-leucyl)carbonyl]-5-[(*tert*-butoxyglycyl)carbonyl]-1*H*-imidazole
**4{***14*}	4-[(benzyloxy-*S*-leucyl)carbonyl]-5-[(*tert*-butoxyglycyl)carbonyl]-1*H*-imidazole
**4{***15*}	4-[(*tert*-butoxy-*S*-phenylalanyl)carbonyl]-5-[(benzyloxyglycyl)carbonyl]-1*H*-imidazole
**4{***16*}	4-[(benzyloxy-*S*-phenylalanyl)carbonyl]-5-[(benzyloxyglycyl)carbonyl]-1*H*-imidazole
**4{***17*}	4-[(*tert*-butoxy*-S*-[*N*^ε^-(*tert*-butoxy)carbonyl]lysyl)carbonyl]-5-[(benzyloxyglycyl)carbonyl]-1*H*-imidazole
**4{***18*}	4-[(*tert*-butoxy-*S*-alanyl)carbonyl]-5-[(benzyloxyglycyl)carbonyl]-1*H*-imidazole
**4{***19*}	4-[(benzyloxy-*S*-alanyl)carbonyl]-5-[(benzyloxyglycyl)carbonyl]-1*H*-imidazole
**4{***20*}	4-[(*tert*-butoxy-*S*-leucyl)carbonyl]-5-[(benzyloxyglycyl)carbonyl]-1*H*-imidazole
**4{***21*}	4-[(benzyloxy-*S*-leucyl)carbonyl]-5-[(benzyloxyglycyl)carbonyl]-1*H*-imidazole
**4{***22*}	4-[(*tert*-butoxy-*S*-phenylalanyl)carbonyl]-5-[(benzyloxyglycyl)carbonyl]-1*H*-imidazole
**4{***23*}	4-[(benzyloxy-*S*-phenylalanyl)carbonyl]-5-[(benzyloxyglycyl)carbonyl]-1*H*-imidazole
**4{***24*}	4-[(*tert*-butoxy*-S*-[*N*^ε^-(*tert*-butoxy)carbonyl]lysyl)carbonyl]-5-[(benzyloxyglycyl)carbonyl]-1*H*-imidazole
**4{***25*}	4-[(benzyloxy-*S*-alanyl)carbonyl]-5-[(*tert*-butoxy-*S*-alanyl)carbonyl]-1*H*-imidazole
**4{***26*}	4-[(*tert*-butoxy-*S*-leucyl)carbonyl]-5-[(*tert*-butoxy-*S*-alanyl)carbonyl]-1*H*-imidazole
**4{***27*}	4-[(benzyloxy- *S*-leucyl)carbonyl]-5-[(*tert*-butoxy-*S*-alanyl)carbonyl]-1*H*-imidazole
**4{***28*}	4-[(*tert*-butoxy-*S*-phenylalanyl)carbonyl]-5-[(*tert*-butoxy-*S*-alanyl)carbonyl]-1*H*-imidazole
**4{***29*}	4-[(benzyloxy-*S*-phenylalanyl)carbonyl]-5-[(*tert*-butoxy-*S*-alanyl)carbonyl]-1*H*-imidazole
**4{***30*}	4-[(*tert*-butoxy*-S*-[*N*^ε^-(*tert*-butoxy)carbonyl]lysyl)carbonyl]-5-[(*tert*-butoxy-*S*-alanyl)carbonyl]-1*H*-imidazole
**4{***31*}	4-[(*tert*-butoxy-*S*-leucyl)carbonyl]-5-[(benzyloxy-*S*-alanyl)carbonyl]-1*H*-imidazole
**4{***32*}	4-[(benzyloxy-*S*-leucyl)carbonyl]-5-[(benzyloxy-*S*-alanyl)carbonyl]-1*H*-imidazole
**4{***33*}	4-[(*tert*-butoxy-*S*-phenylalanyl)carbonyl]-5-[(benzyloxy-*S*-alanyl)carbonyl]-1*H*-imidazole
**4{***34*}	4-[(benzyloxy-*S*-phenylalanyl)carbonyl]-5-[(benzyloxy-*S*-alanyl)carbonyl]-1*H*-imidazole
**4{***35*}	4-[(*tert*-butoxy*-S*-[*N*^ε^-(*tert*-butoxy)carbonyl]lysyl)carbonyl]-5-[(benzyloxy-*S*-alanyl)carbonyl]-1*H*-imidazole
**4{***36*}	4-[(benzyloxy-*S*-leucyl)carbonyl]-5-[(*tert*-butoxy-*S*-leucyl)carbonyl]-1*H*-imidazole
**4{***37*}	4-[(*tert*-butoxy-*S*-phenylalanyl)carbonyl]-5-[(*tert*-butoxy-*S*-leucyl)carbonyl]-1*H*-imidazole
**4{***38*}	4-[(benzyloxy- *S*-phenylalanyl)carbonyl]-5-[(*tert*-butoxy-*S*-leucyl)carbonyl]-1*H*-imidazole
**4{***39*}	4-[(*tert*-butoxy*-S*-[*N*^ε^-(*tert*-butoxy)carbonyl]lysyl)carbonyl]-5-[(*tert*-butoxy-*S*-leucyl)carbonyl]-1*H*-imidazole
**4{***40*}	4-[(*tert*-butoxy-*S*-phenylalanyl)carbonyl]-5-[(benzyloxy-*S*-leucyl)carbonyl]-1*H*-imidazole
**4{***41*}	4-[(benzyloxy- *S*-phenylalanyl)carbonyl]-5-[(benzyloxy-*S*-leucyl)carbonyl]-1*H*-imidazole
**4{***42*}	4-[(*tert*-butoxy*-S*-[*N*^ε^-(*tert*-butoxy)carbonyl]lysyl)carbonyl]-5-[(benzyloxy-*S*-leucyl)carbonyl]-1*H*-imidazole
**4{***43*}	4-[(benzyloxy-*S*-phenylalanyl)carbonyl]-5-[(*tert*-butoxy-*S*-phenylalanyl)carbonyl]-1*H*-imidazole
**4{***44*}	4-[(*tert*-butoxy*-S*-[*N*^ε^-(*tert*-butoxy)carbonyl]lysyl)carbonyl]-5-[(*tert*-butoxy-*S*-phenylalanyl)carbonyl]-1*H*-imidazole
**4{***45*}	4-[(*tert*-butoxy*-S*-[*N*^ε^-(*tert*-butoxy)carbonyl]lysyl)carbonyl]-5-[(benzyloxy-*S*-phenylalanyl)carbonyl]-1*H*-imidazole

## Supplementary Files

Supplementary File 1

Supplementary File 2

Supplementary File 3
