# Supplementary material for: Parallel Synthesis of a Library of Symmetrically- and Dissymmetrically-disubstituted Imidazole-4,5-dicarboxamides Bearing Amino Acid Esters
Source: Molecules. 2009 Jan 13;14(1):352–63. doi: 10.3390/molecules14010352 (PMC2635021; doi:10.3390/molecules14010352)
Supplement: Supplementary File 1 [file molecules-14-00352-s001.pdf]

Parallel Synthesis of a Library of Symmetrically- and Dissymmetrically-disubstituted Imidazole-4,5-dicarboxamides Bearing Amino Acid Esters

*Rosanna Solinas, John C. DiCesare, and Paul W. Baures**

Department of Chemistry and Biochemistry, The University of Tulsa, 800 South Tucker Drive, Tulsa, OK 74104

Supporting Information

Table of Contents

Page S1-2. Title Page and Table of Contents
Page S3. **Table S1.** Symmetrically-disubstituted Amino Acid Ester I45DCs, **4**{*1-9*}.
Page S4. **Table S2.** Dissymmetrically-disubstituted Amino Acid Ester I45DCs, **4**{*10-45*}.
Page S5. **Figure S1.** LC/MS data for **4{**1}.
Page S6. **Figure S2.** LC/MS data for **4{**2}.
Page S7. **Figure S3.** LC/MS data for **4{**3}.
Page S8. **Figure S4.** LC/MS data for **4{**4}.
Page S9. **Figure S5.** LC/MS data for **4{**5}.
Page S10. **Figure S6.** LC/MS data for **4{**6}.
Page S11. **Figure S7.** LC/MS data for **4{**7}.
Page S12. **Figure S8.** LC/MS data for **4{**8}.
Page S13. **Figure S9.** LC/MS data for **4{**9}.
Page S14. **Figure S10.** LC/MS data for **4{**10}.
Page S15. **Figure S11.** LC/MS data for **4{**11}.
Page S16. **Figure S12.** LC/MS data for **4{**12}.
Page S17. **Figure S13.** LC/MS data for **4{**13}.
Page S18. **Figure S14.** LC/MS data for **4{**14}.
Page S19. **Figure S15.** LC/MS data for **4{**15}.
Page S20. **Figure S16.** LC/MS data for **4{**16}.
Page S21. **Figure S17.** LC/MS data for **4{**17}.
Page S22. **Figure S18.** LC/MS data for **4{**18}.
Page S23. **Figure S19.** LC/MS data for **4{**19}.
Page S24. **Figure S20.** LC/MS data for **4{**20}.
Page S25. **Figure S21.** LC/MS data for **4{**21}.
Page S26. **Figure S22.** LC/MS data for **4{**22}.
Page S27. **Figure S23.** LC/MS data for **4{**23}.
Page S28. **Figure S24.** LC/MS data for **4{**24}.
Page S29. **Figure S25.** LC/MS data for **4{**25}.
Page S30 **Figure S26.** LC/MS data for **4{**26}.
Page S31. **Figure S27.** LC/MS data for **4{**27}.
Page S32. **Figure S28.** LC/MS data for **4{**28}.
Page S33. **Figure S29.** LC/MS data for **4{**29}.
Page S34. **Figure S30.** LC/MS data for **4{**30}.
Page S35. **Figure S31.** LC/MS data for **4{**31}.
Page S36. **Figure S32.** LC/MS data for **4{**32}.
Page S37. **Figure S33.** LC/MS data for **4{**33}.
Page S38. **Figure S34.** LC/MS data for **4{**34}.
Page S39. **Figure S35.** LC/MS data for **4{**35}.
Page S40. **Figure S36.** LC/MS data for **4{**36}.
Page S41. **Figure S37.** LC/MS data for **4{**37}.
Page S42. **Figure S38.** LC/MS data for **4{**38}.
Page S43. **Figure S39.** LC/MS data for **4{**39}.
Page S44. **Figure S40.** LC/MS data for **4{**40}.
Page S45. **Figure S41.** LC/MS data for **4{**41}.
Page S46. **Figure S42.** LC/MS data for **4{**42}.
Page S47. **Figure S43.** LC/MS data for **4{**43}.
Page S48. **Figure S44.** LC/MS data for **4{**44}.
Page S49. **Figure S45.** LC/MS data for **4{**45}.
Page S50. **Figure S46.** LC/MS data for the crude reaction to yield **4{**1}.
Page S51. **Figure S47.** LC/MS data for the crude reaction to yield **4{**7}.

Page S52. **Figure S48.** LC/MS data for the crude reaction to yield **4{**8}.
Page S53. **Figure S49.** LC/MS data for the crude reaction to yield **4{**16}.
Page S54. **Figure S50.** LC/MS data for the crude reaction to yield **4{**24}.
Page S55. **Figure S51.** LC/MS data for the crude reaction to yield **4{**26}.
Page S56. **Figure S52.** LC/MS data for the crude reaction to yield **4{**30}.
Page S57. **Figure S53.** LC/MS data for the crude reaction to yield **4{**32}.
Page S58. **Figure S54.** LC/MS data for the crude reaction to yield **4{**35}.
Page S59. **Figure S55.** LC/MS data for the crude reaction to yield **4{**40}.
Page S60. **Figure S56.** ^{1}H-NMR for **4{**7}.
Page S61. **Figure S57.** ^{1}H-NMR for **4{**8}.
Page S62. **Figure S58.** ^{1}H-NMR for **4{**14}.
Page S63. **Figure S59.** ^{1}H-NMR for **4{**17}.
Page S64. **Figure S60.** ^{1}H-NMR for **4{**22}.
Page S65. **Figure S61.** ^{1}H-NMR for **4{**23}.
Page S66. **Figure S62.** ^{1}H-NMR for **4{**25}.
Page S67. **Figure S63.** ^{1}H-NMR for **4{**26}.
Page S68. **Figure S64.** ^{1}H-NMR for **4{**30}.
Page S69. **Figure S65.** ^{1}H-NMR for **4{**35}.
Page S70. **Figure S66.** ^{1}H-NMR for **4{**38}.
Page S71. **Figure S67.** ^{1}H-NMR for **4{**39}.
Page S72. **Figure S68.** ^{1}H-NMR for **4{**40}.
Page S73. **Figure S69.** ^{1}H-NMR for **4{**42}.
Page S74. **Figure S70.** ^{1}H-NMR for **4{**43}.
Page S75. **Figure S71.** ^{1}H-NMR for the crude reaction to yield **4{**1}.
Page S76. **Figure S72.** ^{1}H-NMR for the crude reaction to yield **4{**7}.
Page S77. **Figure S73.** ^{1}H-NMR for the crude reaction to yield **4{**8}.
Page S78. **Figure S74.** ^{1}H-NMR for the crude reaction to yield **4{**16}.
Page S79. **Figure S75.** ^{1}H-NMR for the crude reaction to yield **4{**24}.
Page S80. **Figure S76.** ^{1}H-NMR for the crude reaction to yield **4{**26}.
Page S81. **Figure S77.** ^{1}H-NMR for the crude reaction to yield **4{**30}.
Page S82. **Figure S78.** ^{1}H-NMR for the crude reaction to yield **4{**32}.
Page S83. **Figure S79.** ^{1}H-NMR for the crude reaction to yield **4{**35}.
Page S84. **Figure S80.** ^{1}H-NMR for the crude reaction to yield **4{**40}.

Table S1. Symmetrically-disubstituted Amino Acid Ester I45DCs, **4**{*1-9*}.

compound	formula	MW	C log*P*	form	R_f	R_t (min)
**4**{*1*}	$C_{17}H_{26}N_4O_6$	382.41	0.35	solid	0.79	3.40
**4**{*2*}	$C_{23}H_{22}N_4O_6$	450.44	1.30	solid	0.22	4.06
**4**{*3*}	$C_{19}H_{30}N_4O_6$	410.47	0.97	glass film	0.69	4.91
**4**{*4*}	$C_{25}H_{26}N_4O_6$	478.50	1.92	glass film	0.12	5.04
**4**{*5*}	$C_{25}H_{42}N_4O_6$	494.62	3.89	glass film	0.45	8.39
**4**{*6*}	$C_{31}H_{38}N_4O_6$	562.66	4.83	glass film	0.52	8.11
**4**{*7*}	$C_{31}H_{38}N_4O_6$	562.66	3.81	glass film	0.62	7.73
**4**{*8*}	$C_{37}H_{34}N_4O_6$	630.69	4.76	solid	0.23	7.37
**4**{*9*}	$C_{35}H_{60}N_6O_{10}$	724.44	3.86	glass film	0.86	7.14

Table S2. Dissymmetrically-disubstituted Amino Acid Ester I45DCs, **4**{*10-45*}.

compound	formula	MW	C log*P*	form	R_f	R_t (min)
**4**{*10*}	$C_{20}H_{24}N_4O_6$	416.43	1.17	glass film	0.83	3.67
**4**{*11*}	$C_{18}H_{28}N_4O_6$	396.44	0.66	glass film	0.86	4.00
**4**{*12*}	$C_{21}H_{26}N_4O_6$	430.45	1.14	glass film	0.85	4.31
**4**{*13*}	$C_{21}H_{34}N_4O_6$	438.52	2.12	glass film	0.91	5.86
**4**{*14*}	$C_{24}H_{32}N_4O_6$	472.53	2.59	glass film	0.83	6.04
**4**{*15*}	$C_{24}H_{32}N_4O_6$	472.53	2.42	glass film	0.78	6.00
**4**{*16*}	$C_{27}H_{30}N_4O_6$	506.55	2.90	glass film	0.84	6.18
**4**{*17*}	$C_{26}H_{43}N_5O_8$	553.31	2.11	glass film	0.61	5.45
**4**{*18*}	$C_{21}H_{26}N_4O_6$	430.56	1.48	glass film	0.56	4.36
**4**{*19*}	$C_{24}H_{24}N_4O_6$	464.47	1.61	solid	0.70	4.54
**4**{*20*}	$C_{24}H_{32}N_4O_6$	472.53	2.93	glass film	0.69	6.06
**4**{*21*}	$C_{27}H_{30}N_4O_6$	506.55	3.07	glass film	0.55	6.06
**4**{*22*}	$C_{27}H_{30}N_4O_6$	506.55	2.56	glass film	0.67	5.90
**4**{*23*}	$C_{30}H_{28}N_4O_6$	540.57	3.03	glass film	0.64	5.95
**4**{*24*}	$C_{29}H_{41}N_5O_8$	587.67	2.92	glass film	0.62	5.93
**4**{*25*}	$C_{22}H_{28}N_4O_6$	444.48	1.45	glass film	0.57	5.37
**4**{*26*}	$C_{22}H_{36}N_4O_6$	452.54	2.43	glass film	0.37	7.44
**4**{*27*}	$C_{25}H_{34}N_4O_6$	486.56	2.90	glass film	0.45	6.66
**4**{*28*}	$C_{25}H_{34}N_4O_6$	486.56	2.73	glass film	0.54	6.53
**4**{*29*}	$C_{28}H_{32}N_4O_6$	520.58	3.20	glass film	0.56	6.38
**4**{*30*}	$C_{27}H_{45}N_5O_8$	567.68	2.42	glass film	0.77	6.13
**4**{*31*}	$C_{25}H_{34}N_4O_6$	486.56	3.24	glass film	0.39	6.80
**4**{*32*}	$C_{28}H_{32}N_4O_6$	520.58	3.38	glass film	0.40	6.79
**4**{*33*}	$C_{28}H_{32}N_4O_6$	520.58	2.86	glass film	0.59	6.38
**4**{*34*}	$C_{31}H_{30}N_4O_6$	554.59	3.34	glass film	0.35	6.52
**4**{*35*}	$C_{39}H_{43}N_5O_8$	601.31	2.89	glass film	0.63	6.25
**4**{*36*}	$C_{28}H_{40}N_4O_6$	528.64	4.36	glass film	0.51	8.31
**4**{*37*}	$C_{28}H_{40}N_4O_6$	528.64	4.19	glass film	0.42	7.99
**4**{*38*}	$C_{31}H_{38}N_4O_6$	562.66	4.66	glass film	0.39	7.79
**4**{*39*}	$C_{30}H_{51}N_5O_8$	609.76	3.87	glass film	0.33	7.48
**4**{*40*}	$C_{31}H_{38}N_4O_6$	562.66	4.32	glass film	0.44	7.95
**4**{*41*}	$C_{34}H_{36}N_4O_6$	596.67	4.80	glass film	0.47	7.64
**4**{*42*}	$C_{33}H_{49}N_5O_8$	643.77	4.35	glass film	0.29	7.39
**4**{*43*}	$C_{34}H_{36}N_4O_6$	596.67	4.28	glass film	0.48	7.44
**4**{*44*}	$C_{33}H_{49}N_5O_8$	643.77	4.17	glass film	0.59	7.29
**4**{*45*}	$C_{36}H_{47}N_5O_8$	677.34	4.65	glass film	0.56	7.29

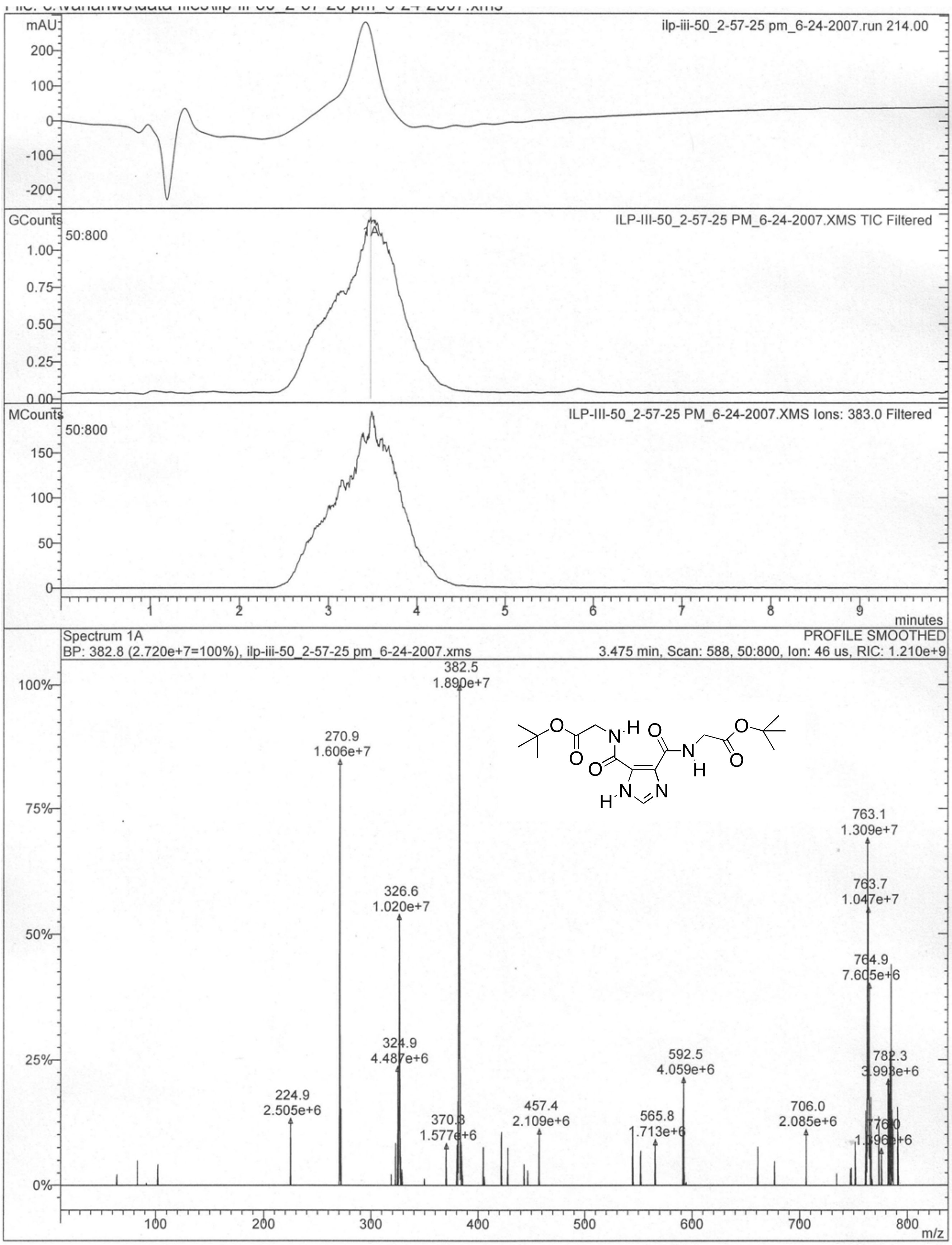

Figure S1. LC/MS data for **4{**1}.

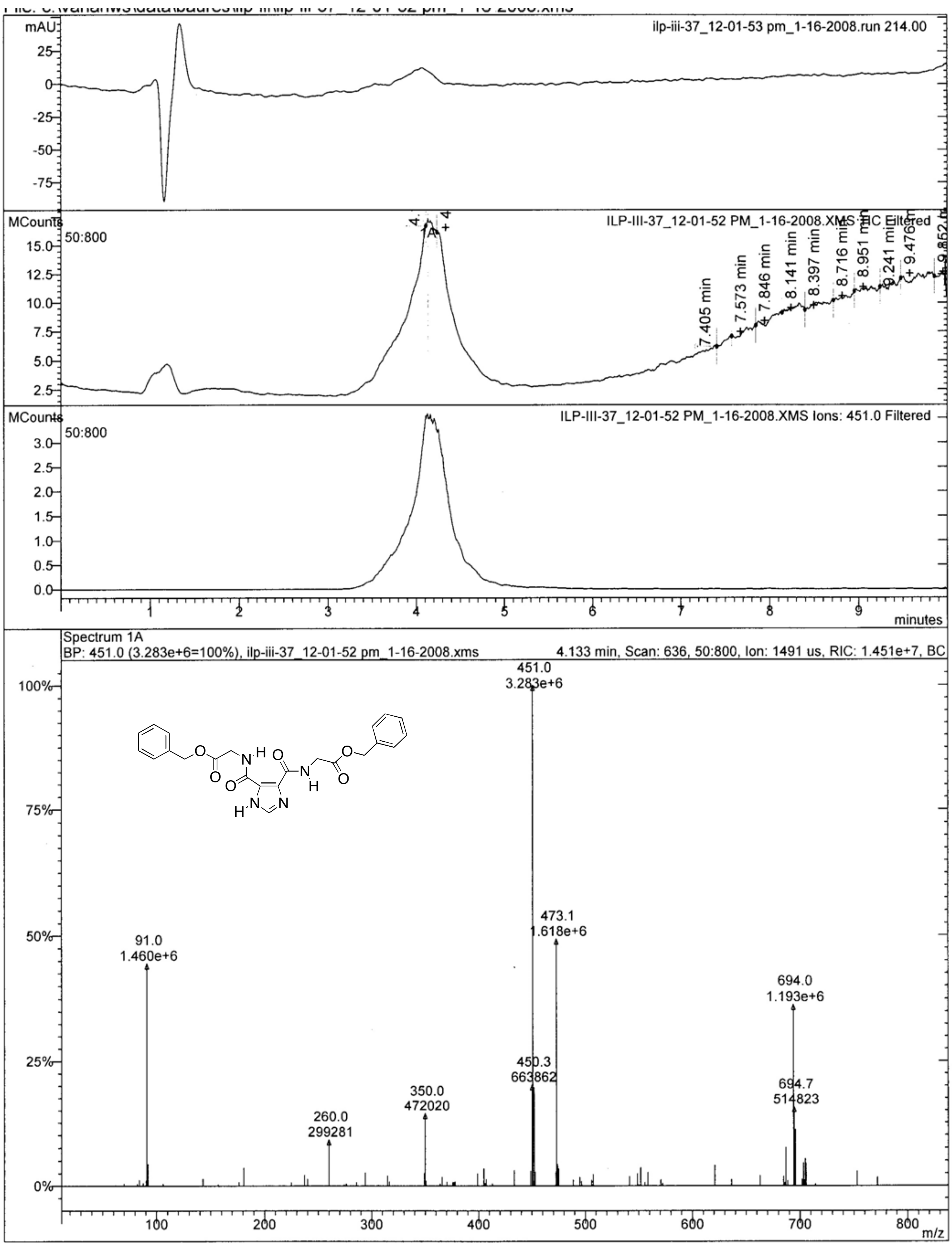

Figure S2. LC/MS data for **4{**2}.

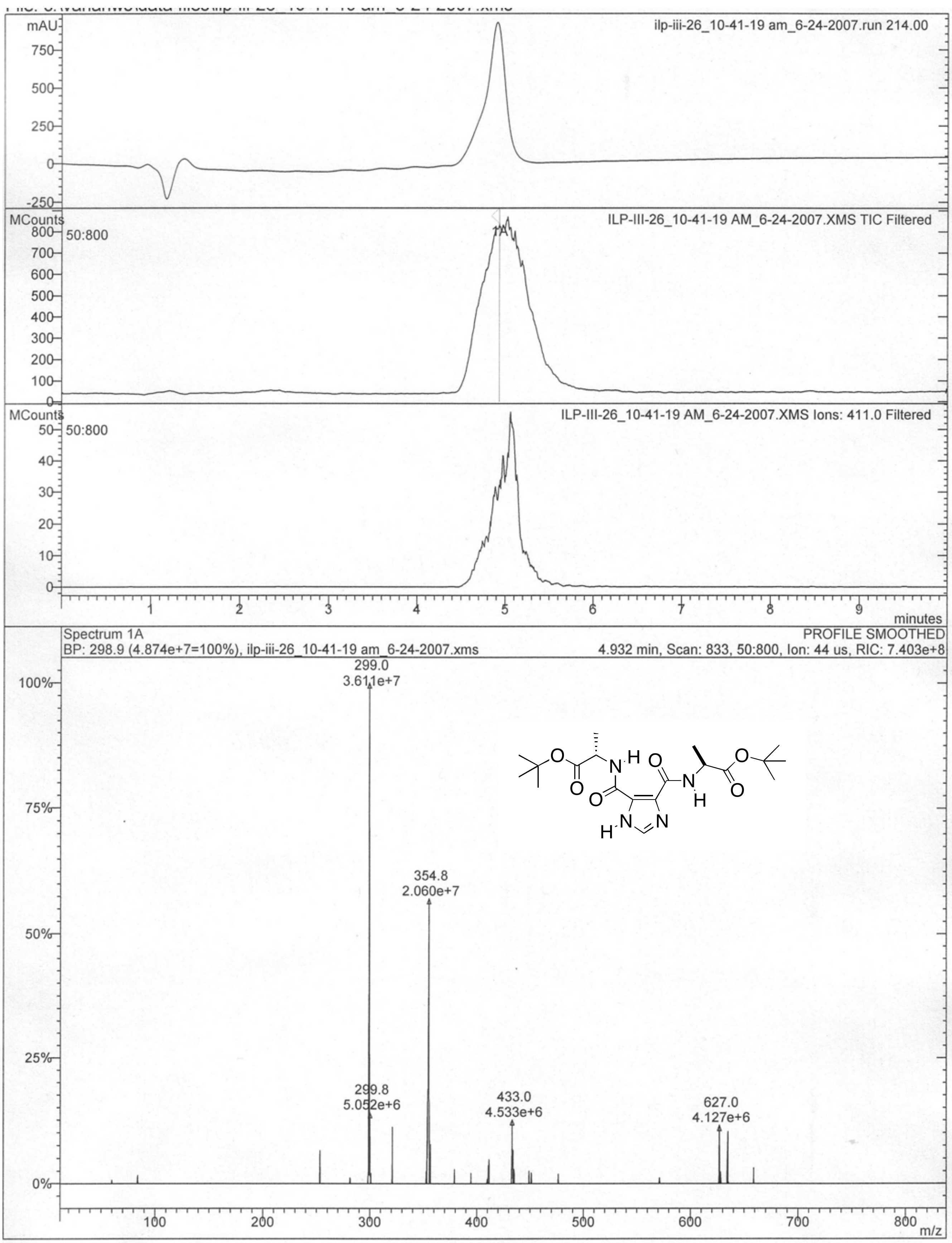

Figure S3. LC/MS data for **4{**3}.

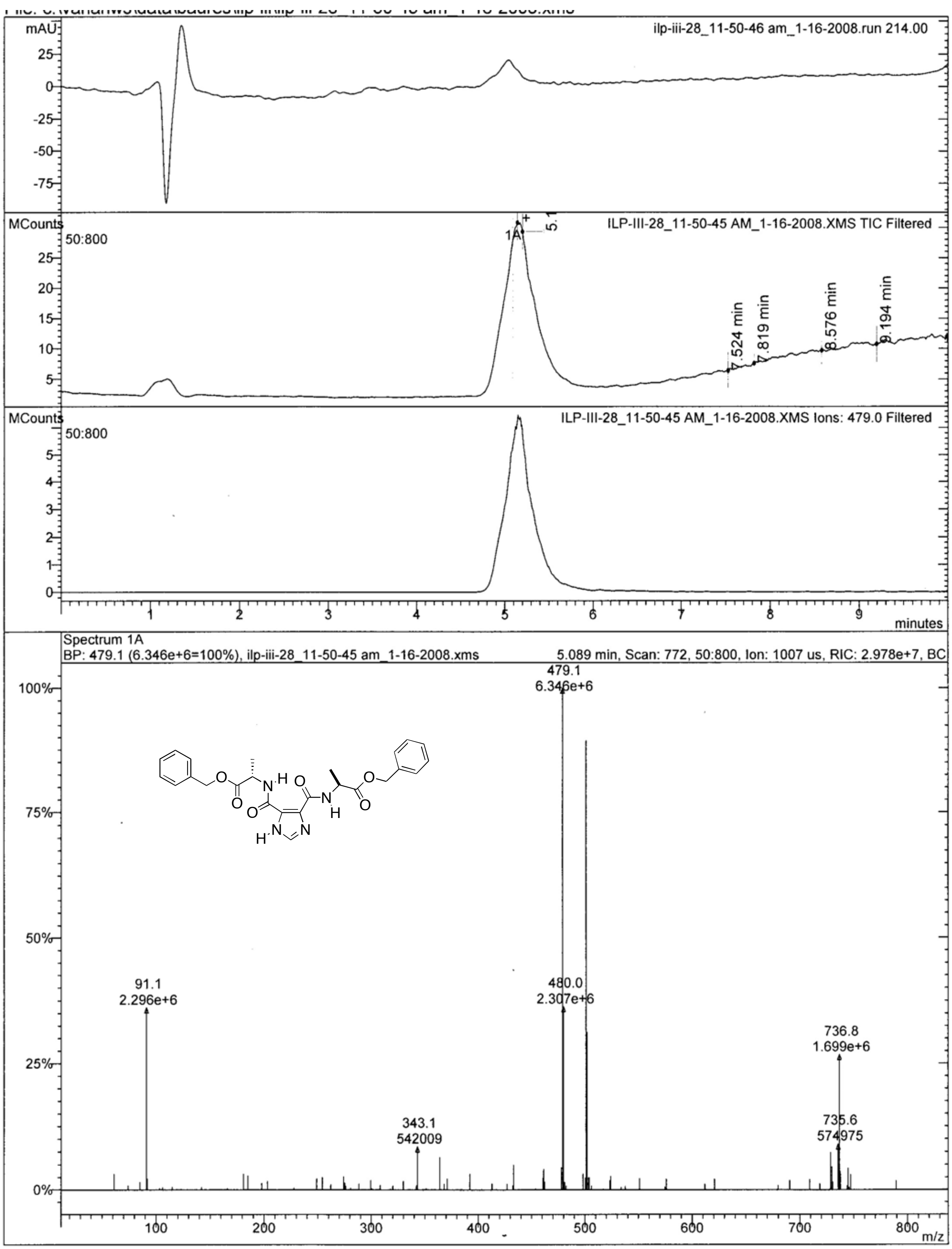

Figure S4. LC/MS data for **4{**4}.

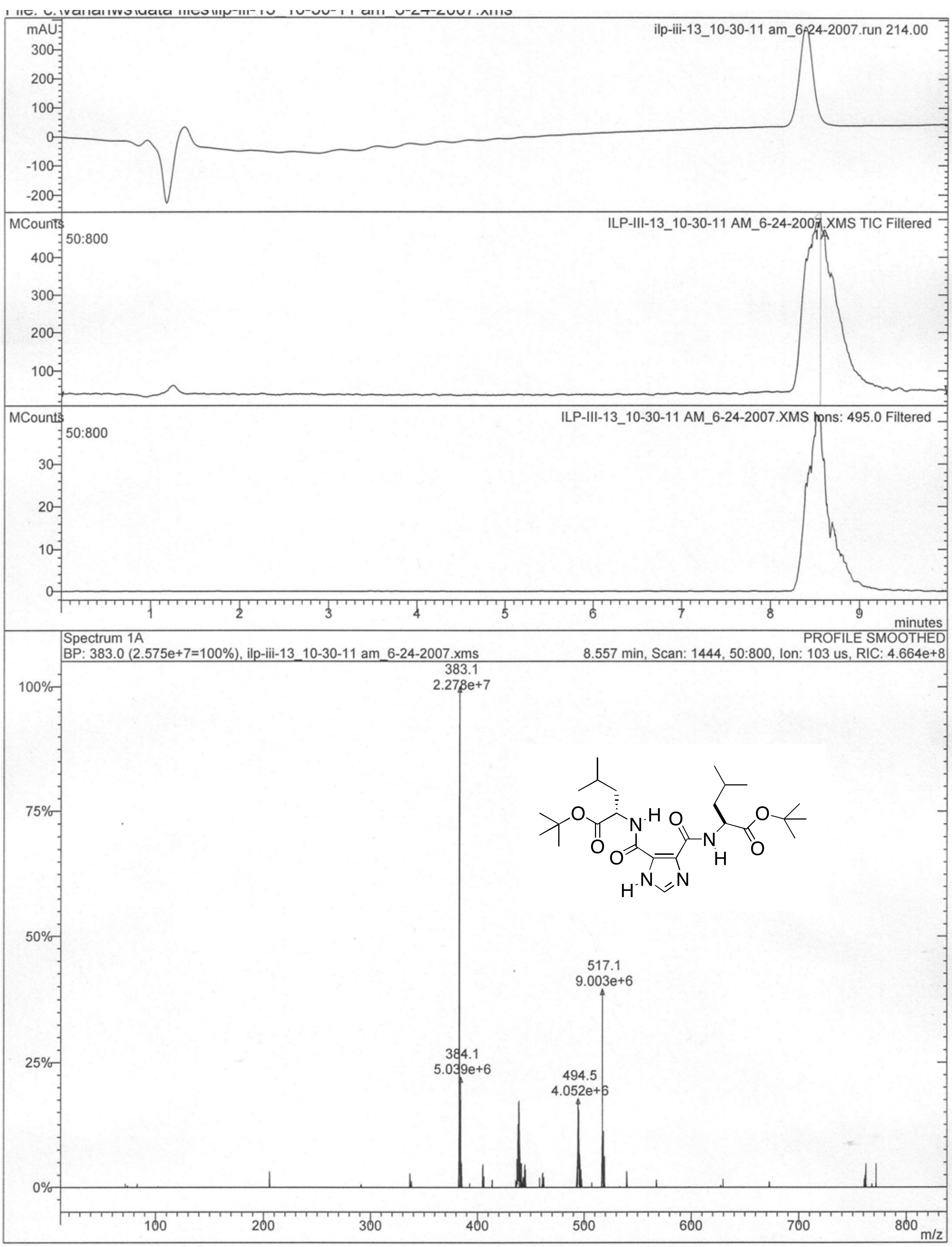

Figure S5. LC/MS data for **4{**5}.

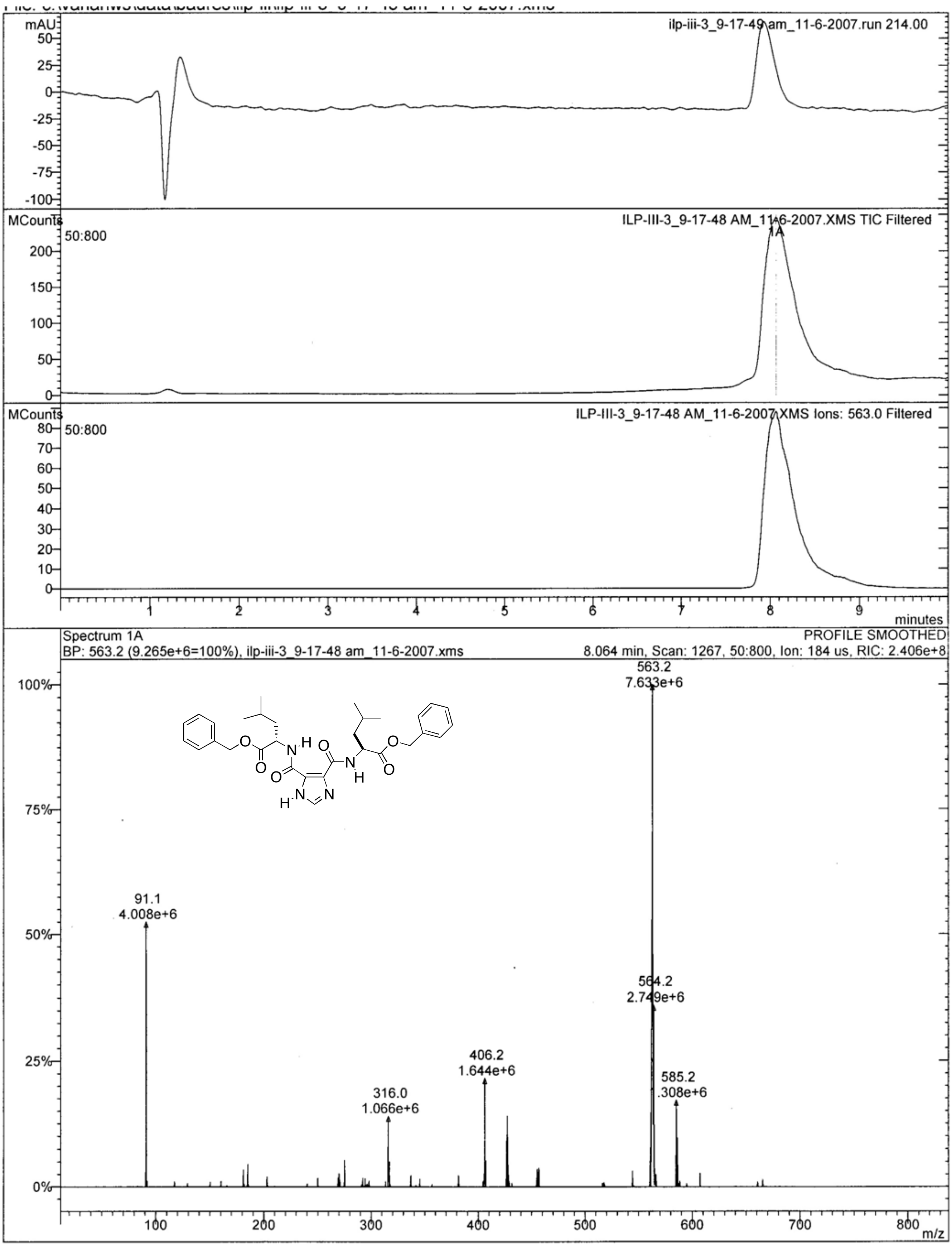

Figure S6. LC/MS data for **4{**6}.

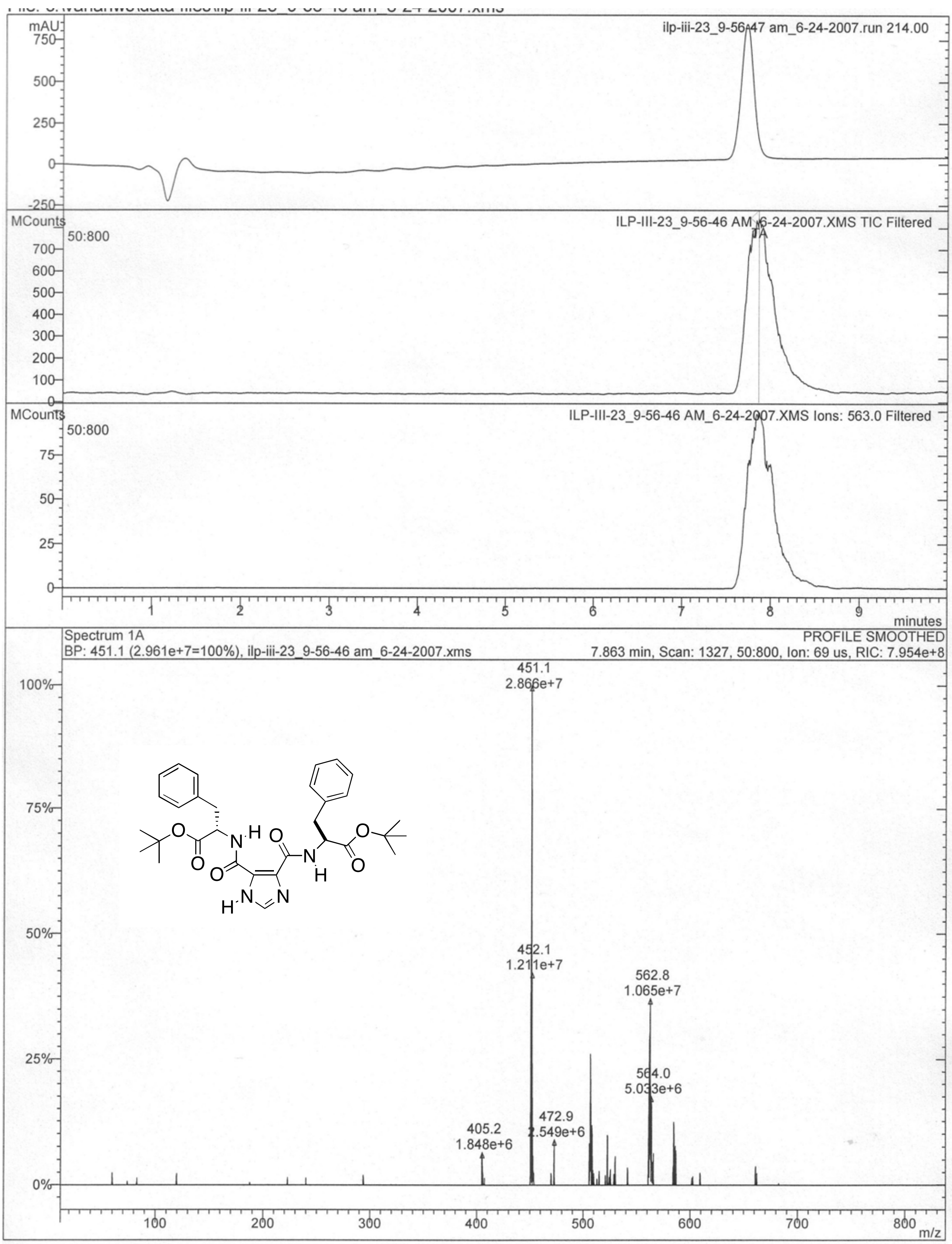

Figure S7. LC/MS data for **4{**7}.

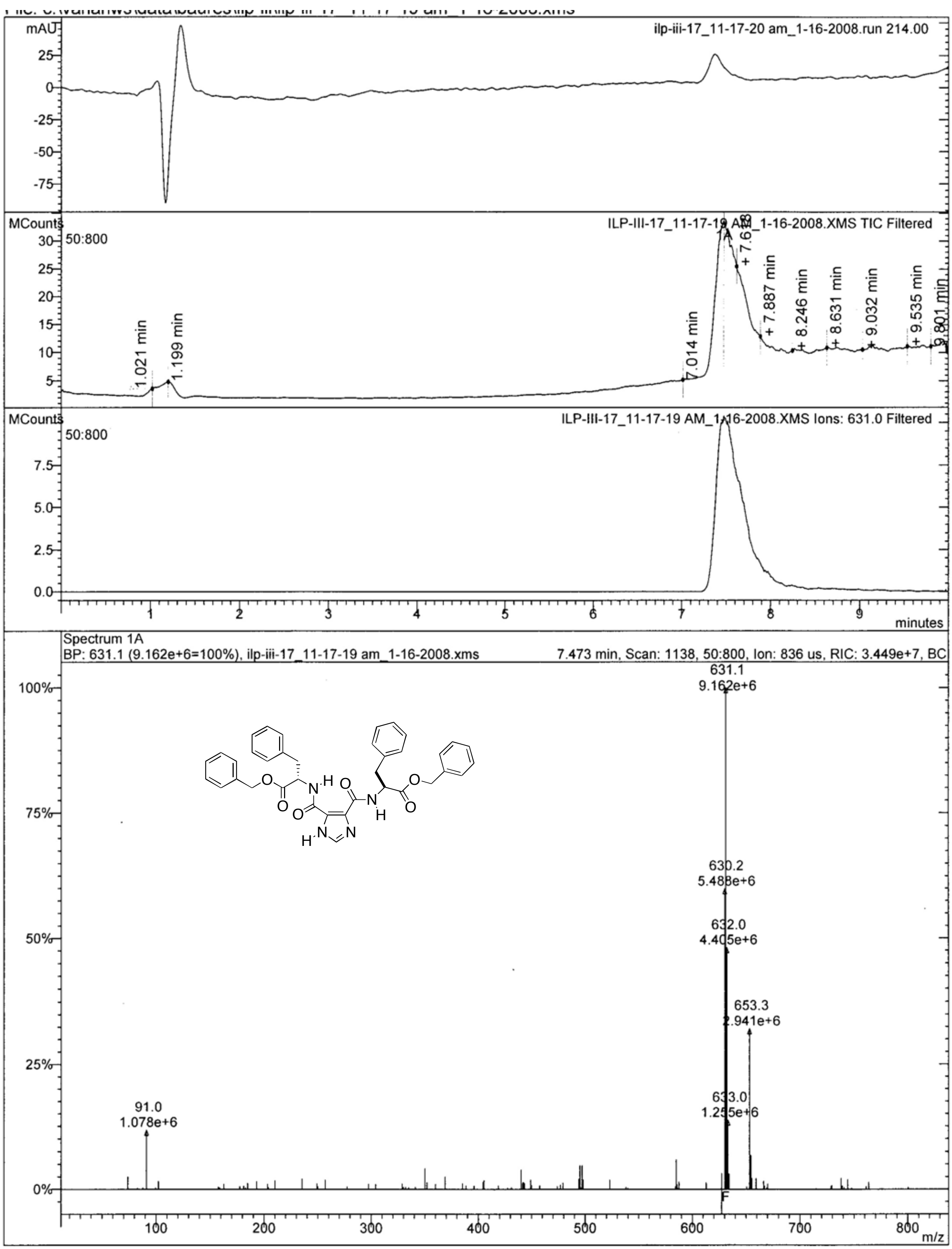

Figure S8. LC/MS data for **4{**8}.

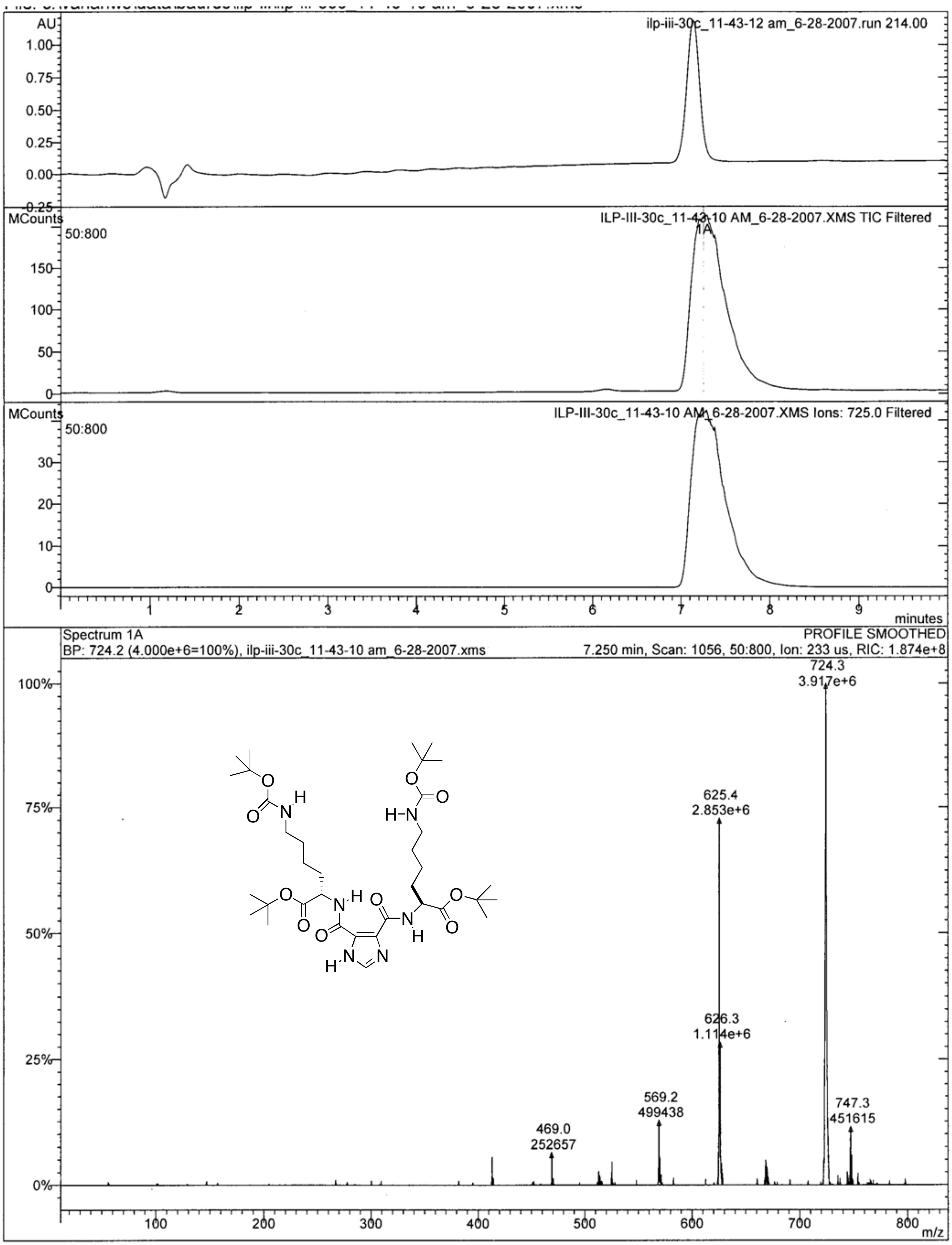

Figure S9. LC/MS data for **4{**9}.

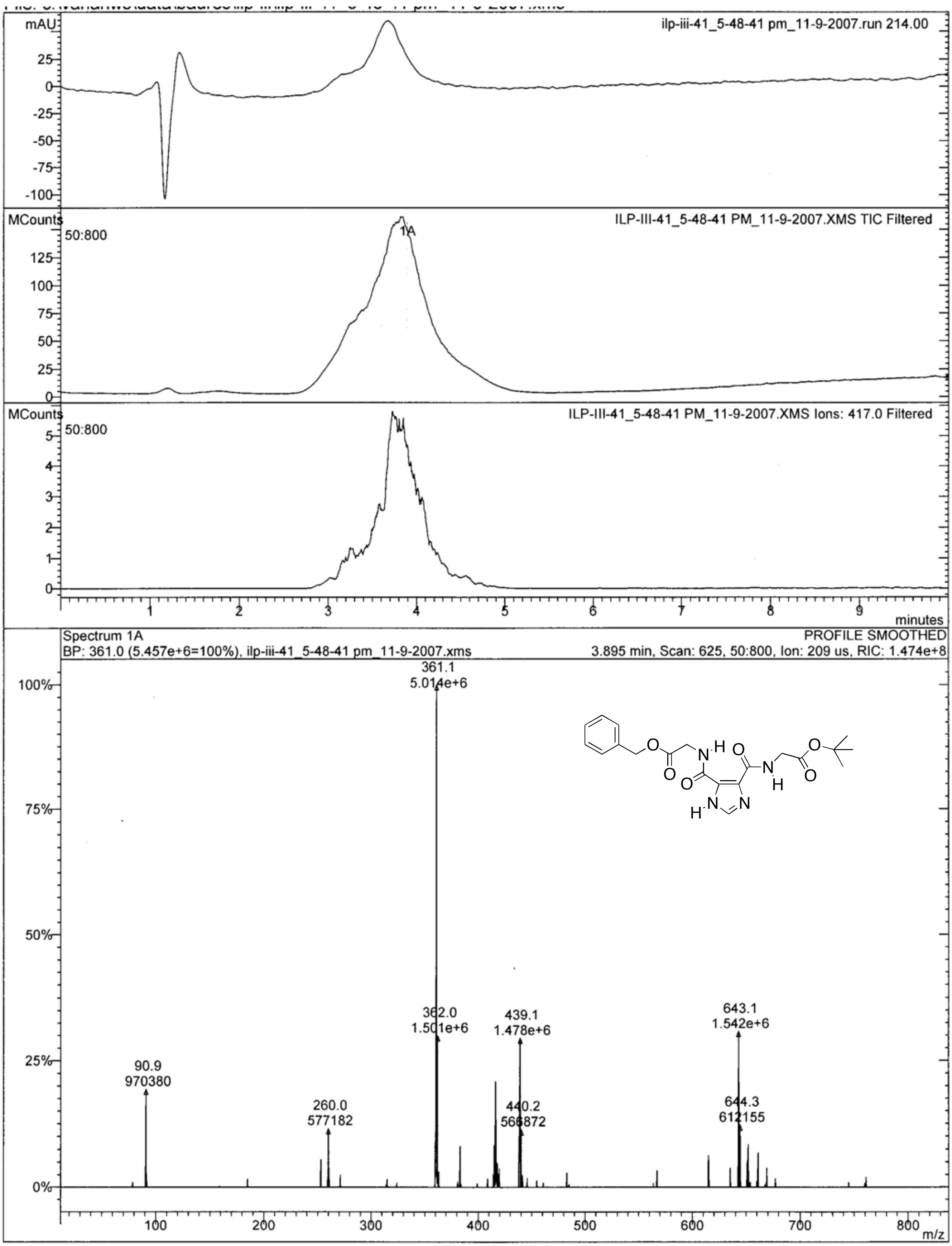

Figure S10. LC/MS data for **4{**10}.

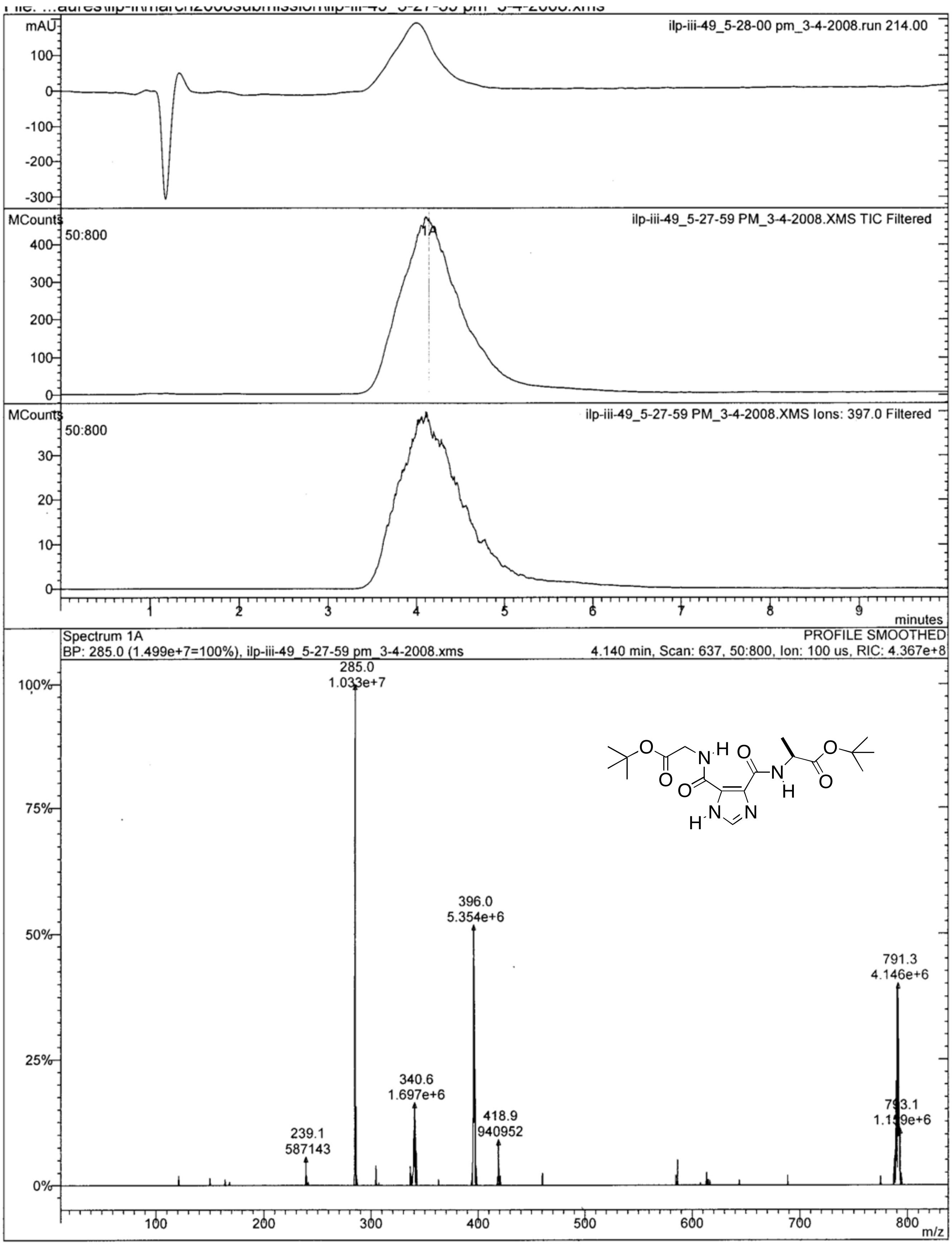

Figure S11. LC/MS data for **4{**11}.

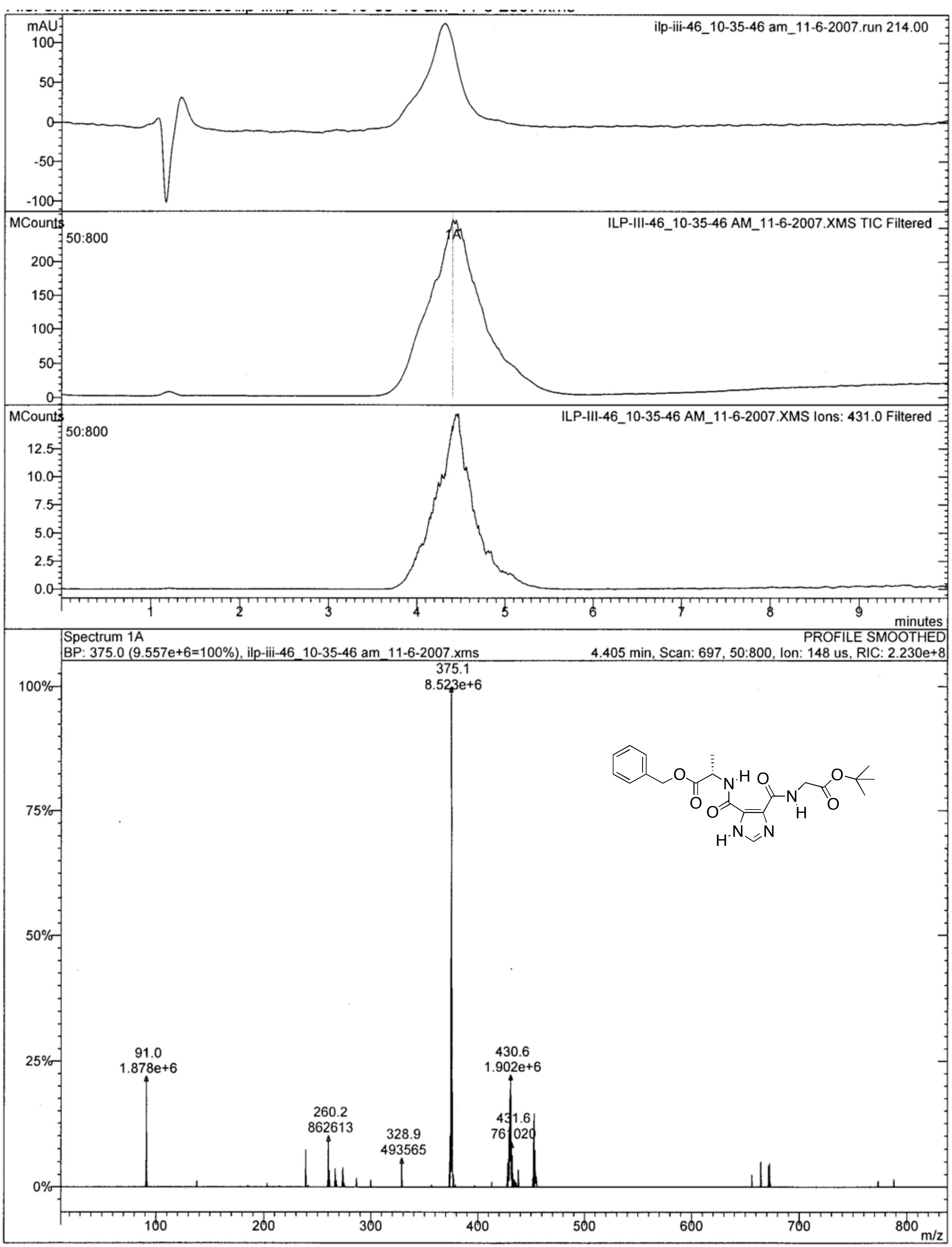

Figure S12. LC/MS data for **4{**12}.

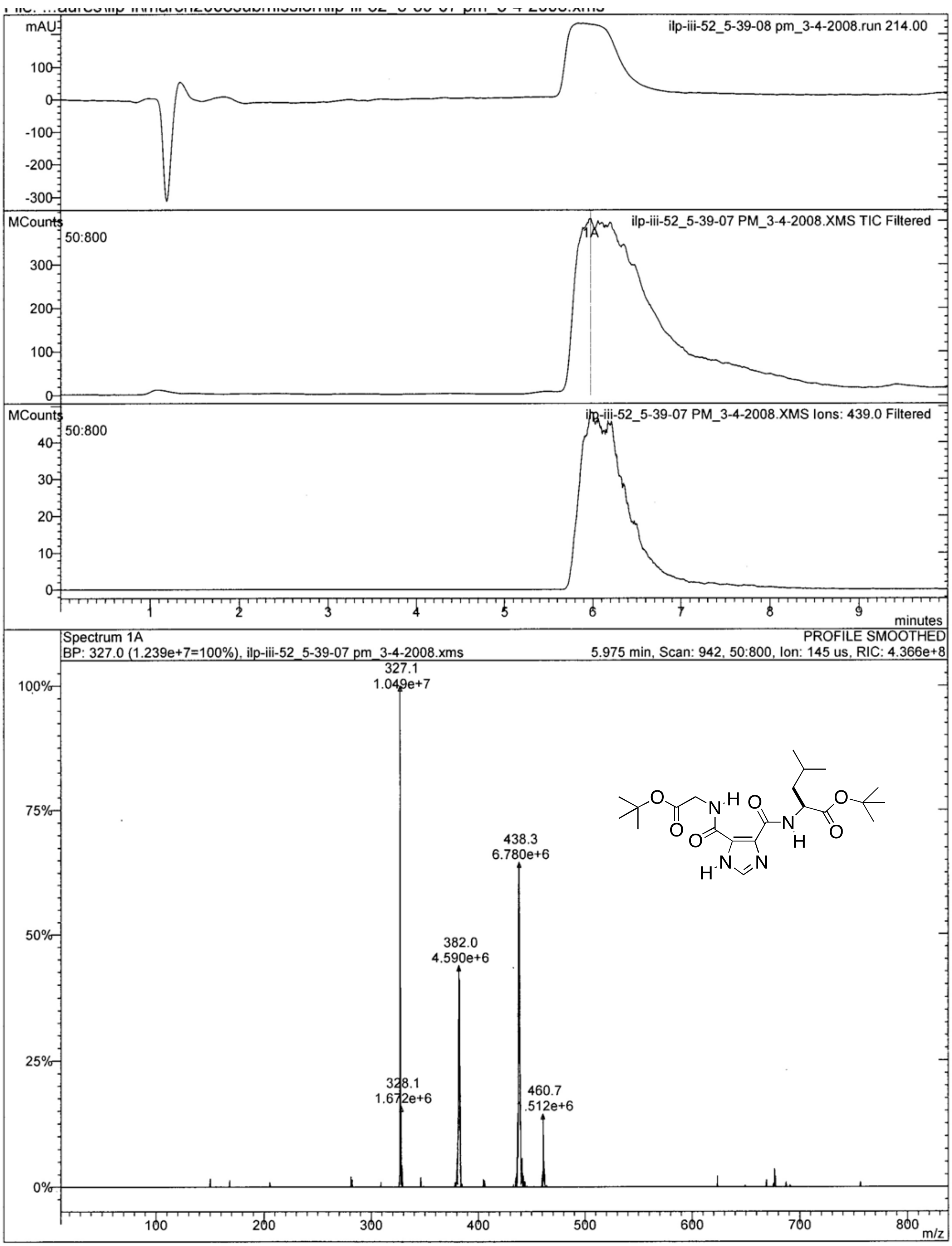

Figure S13. LC/MS data for **4{**13}.

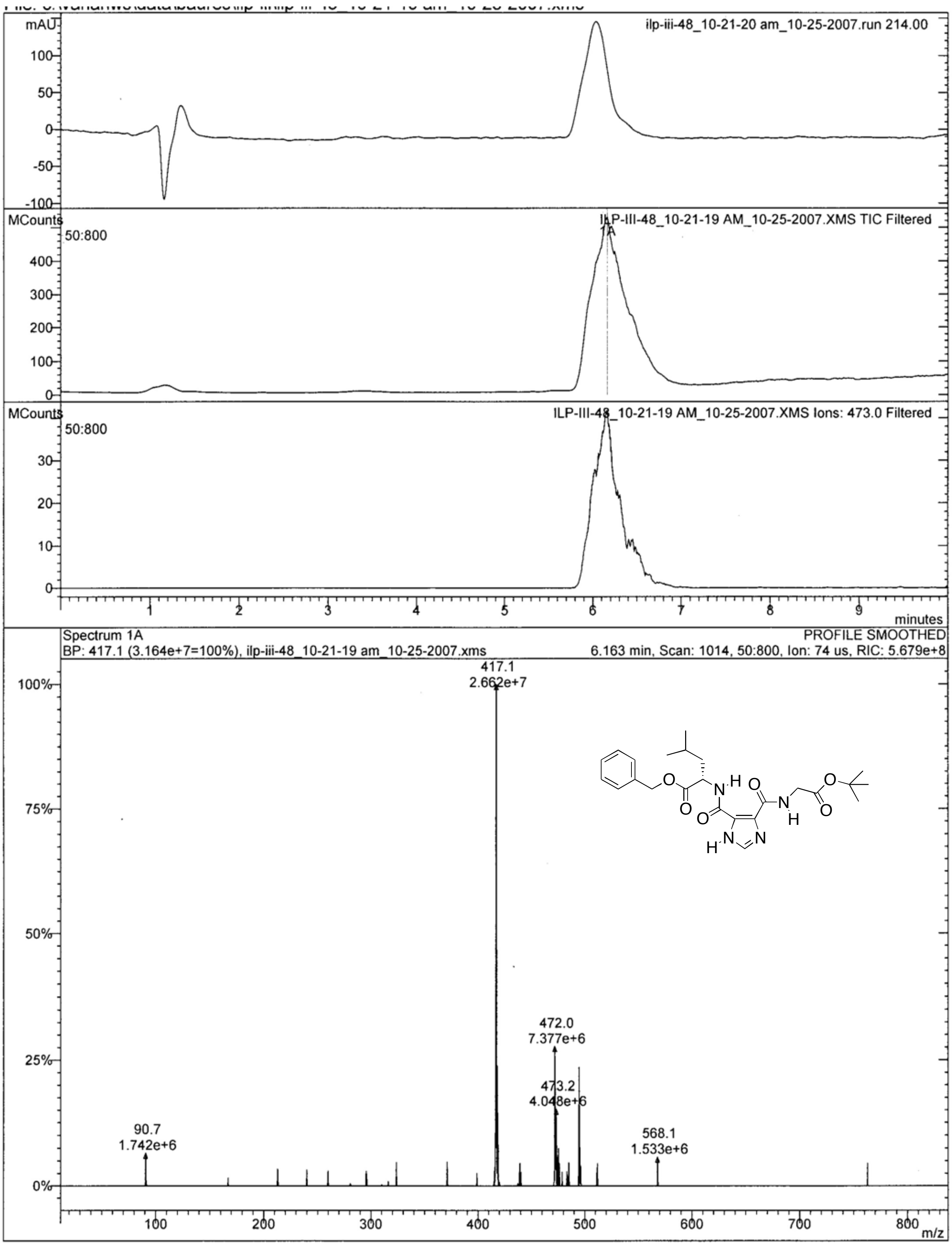

Figure S14. LC/MS data for **4{**14}.

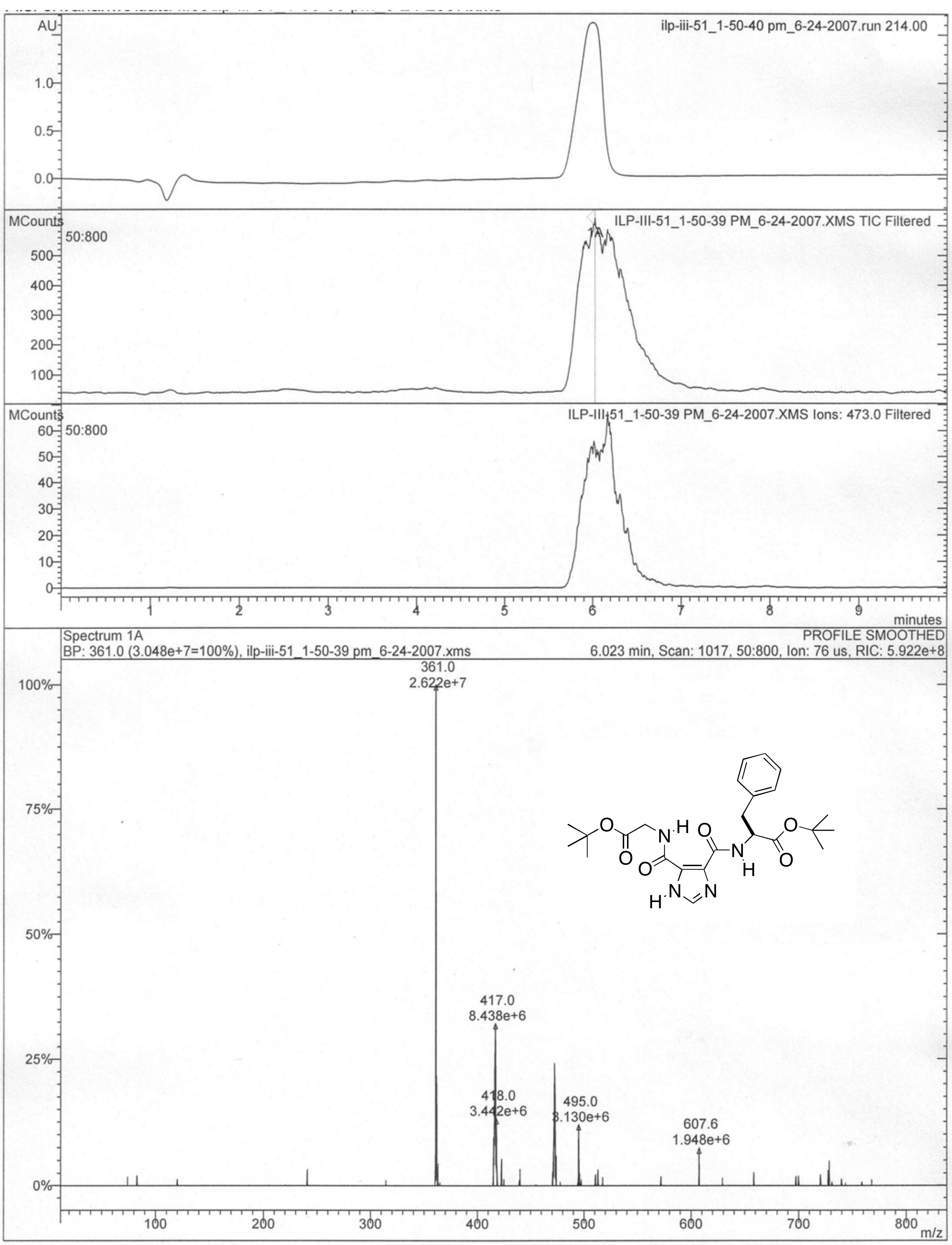

Figure S15. LC/MS data for **4{**15}.

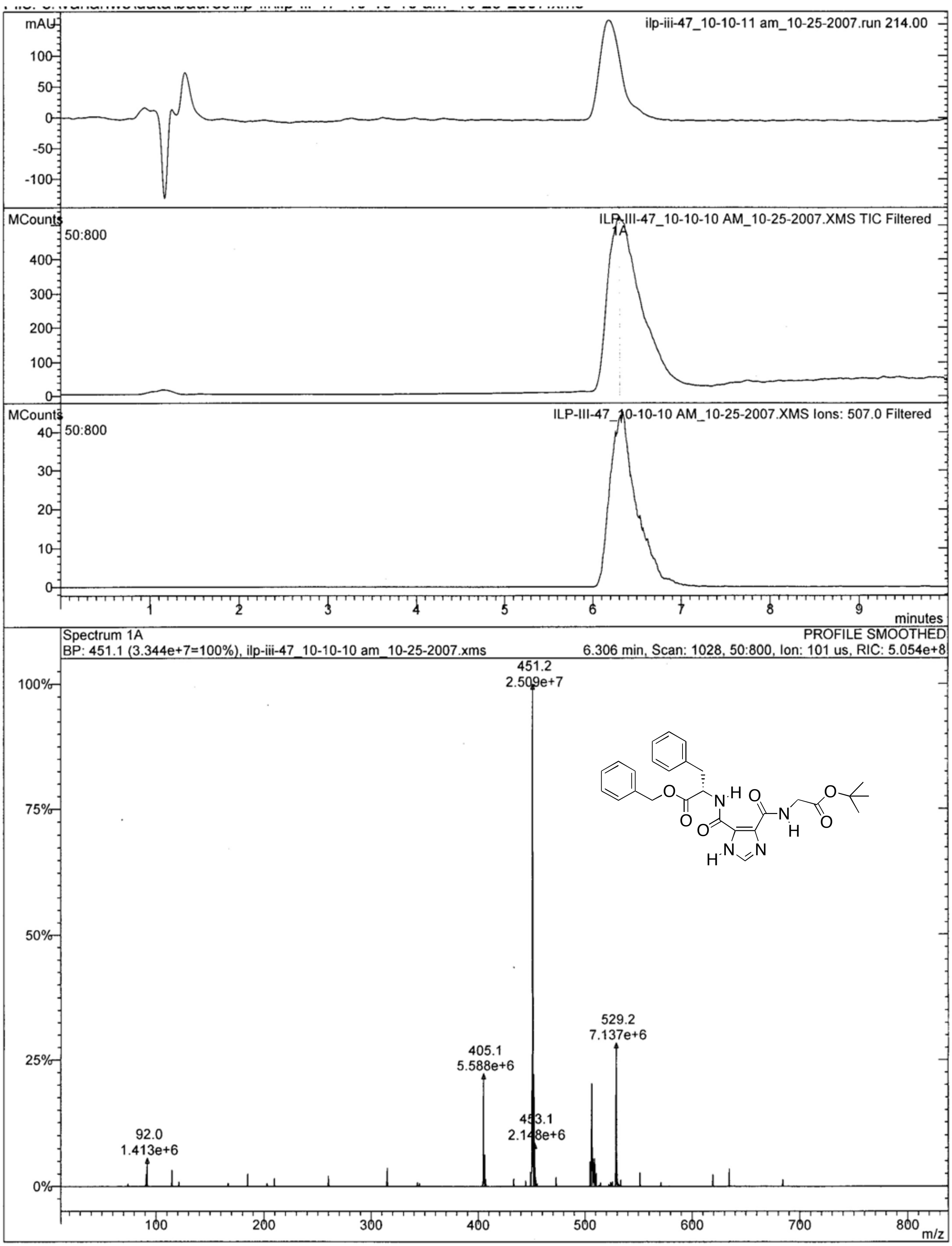

Figure S16. LC/MS data for **4{**16}.

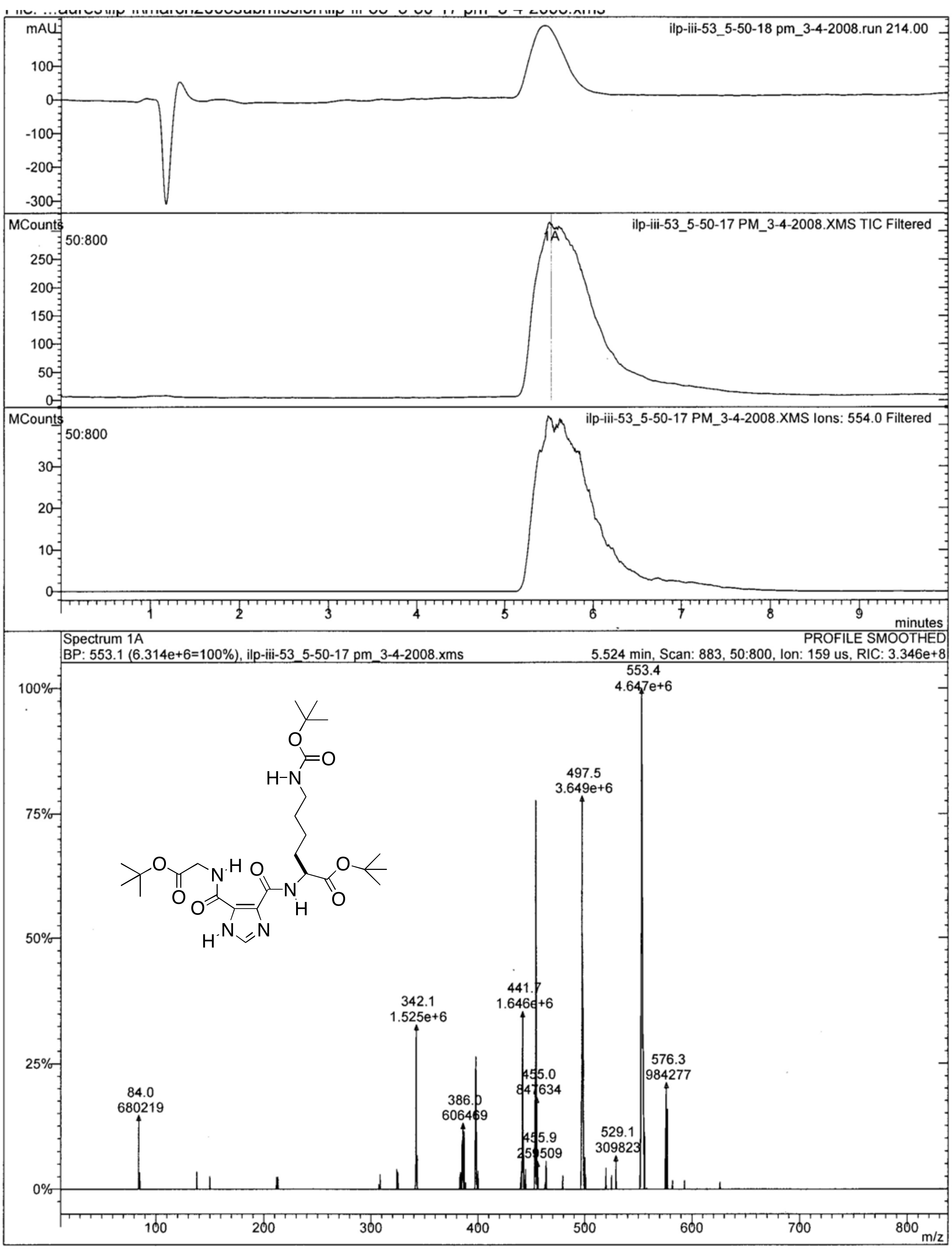

Figure S17. LC/MS data for **4{**17}.

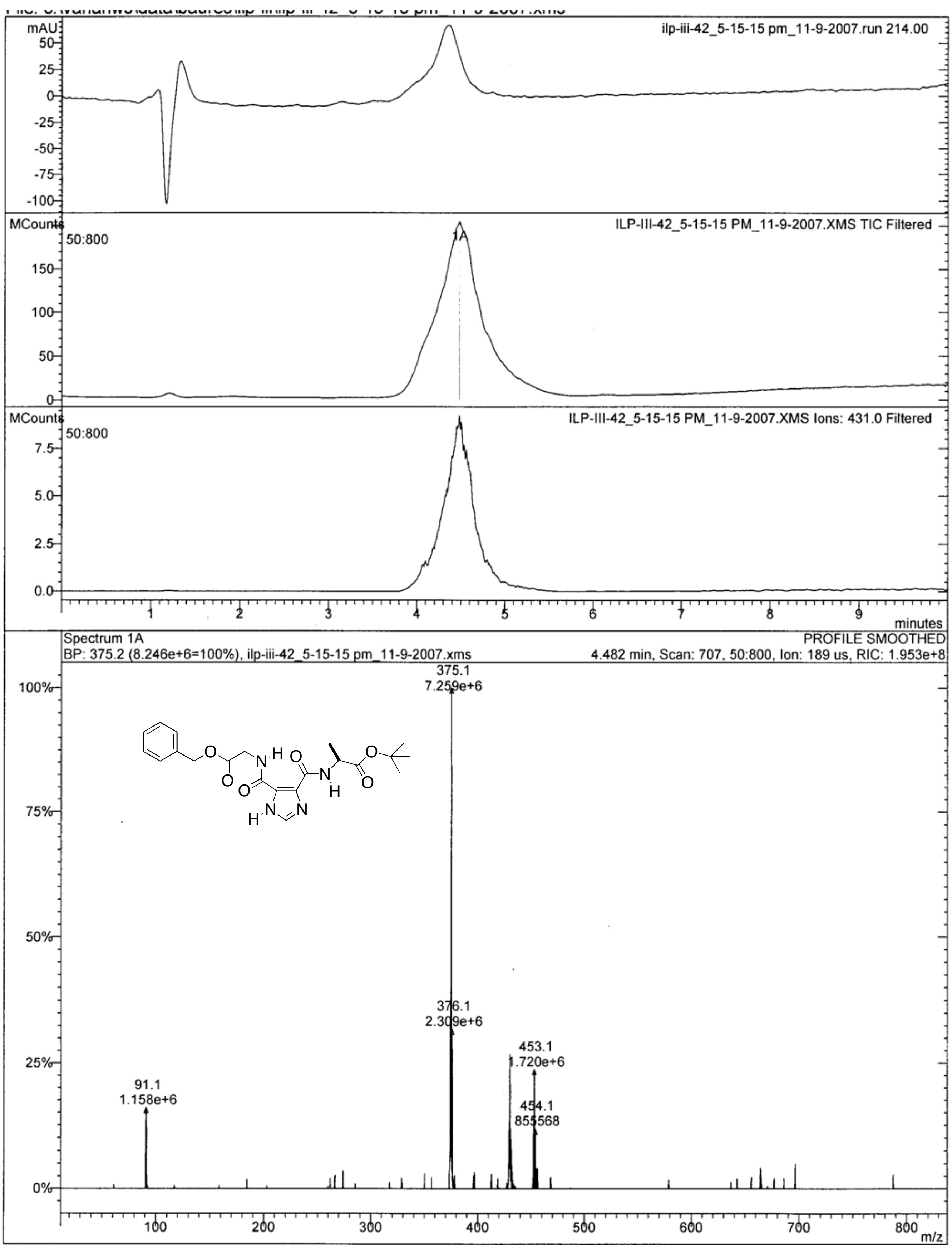

Figure S18. LC/MS data for **4{**18}.

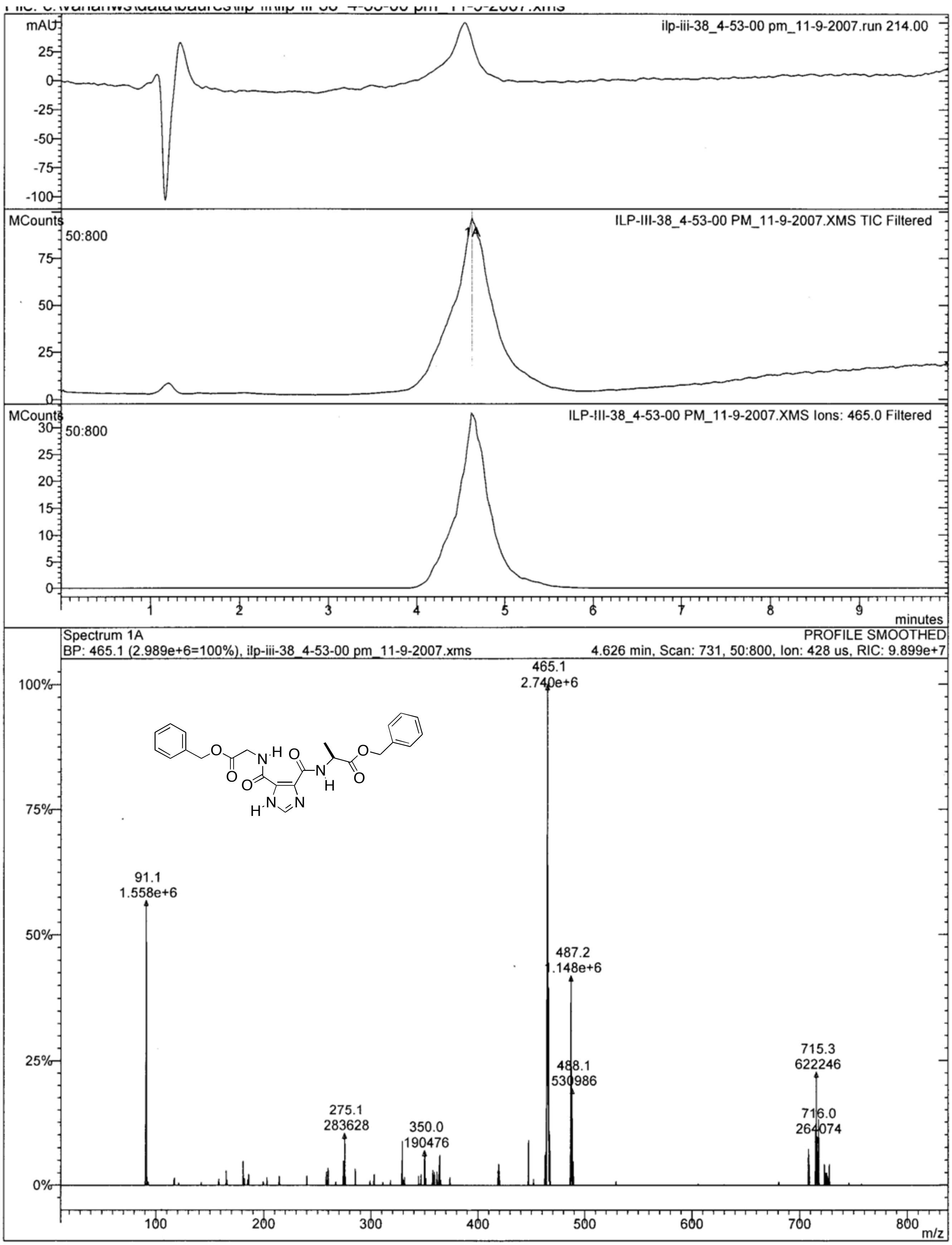

Figure S19. LC/MS data for **4{**19}.

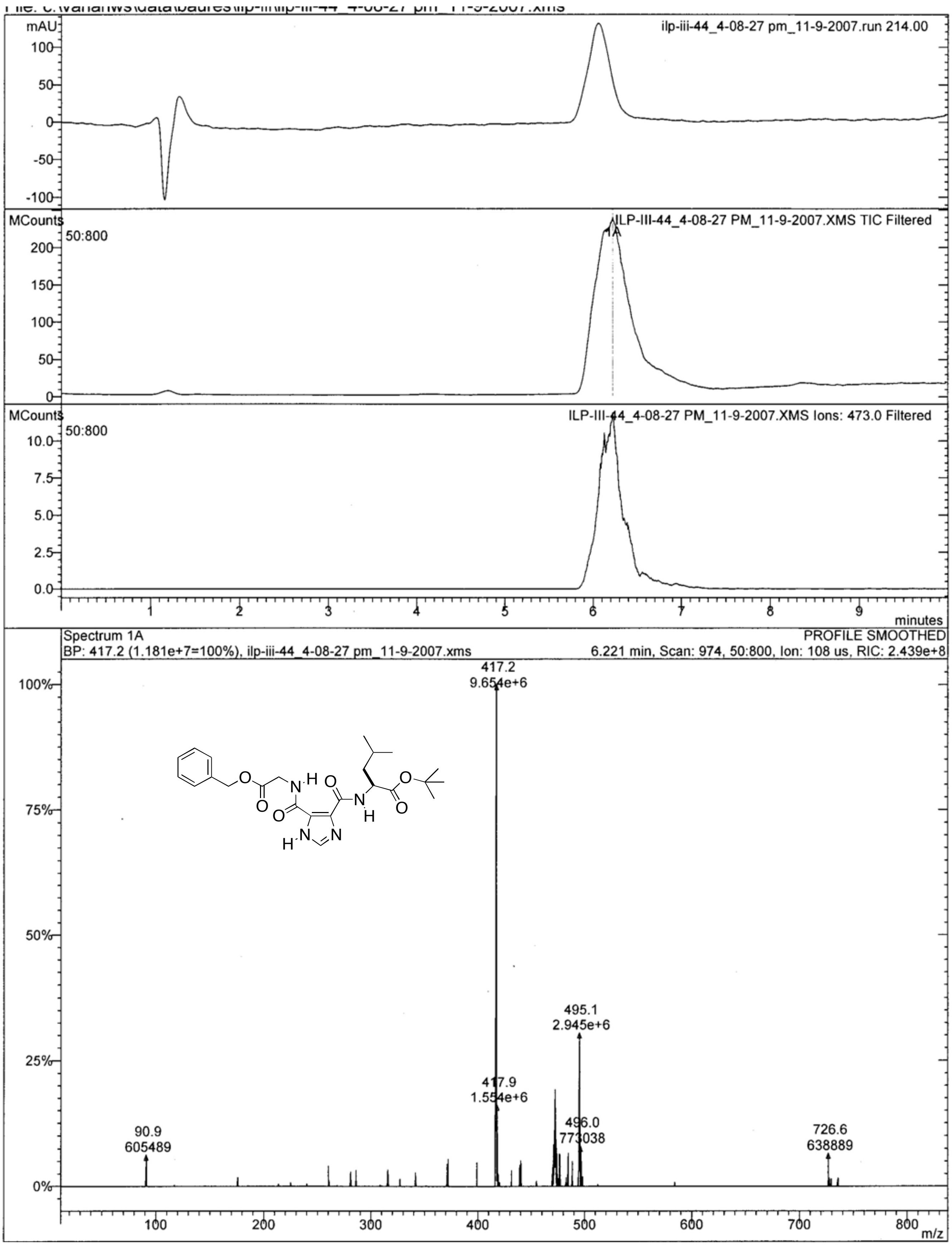

Figure S20. LC/MS data for **4{**20}.

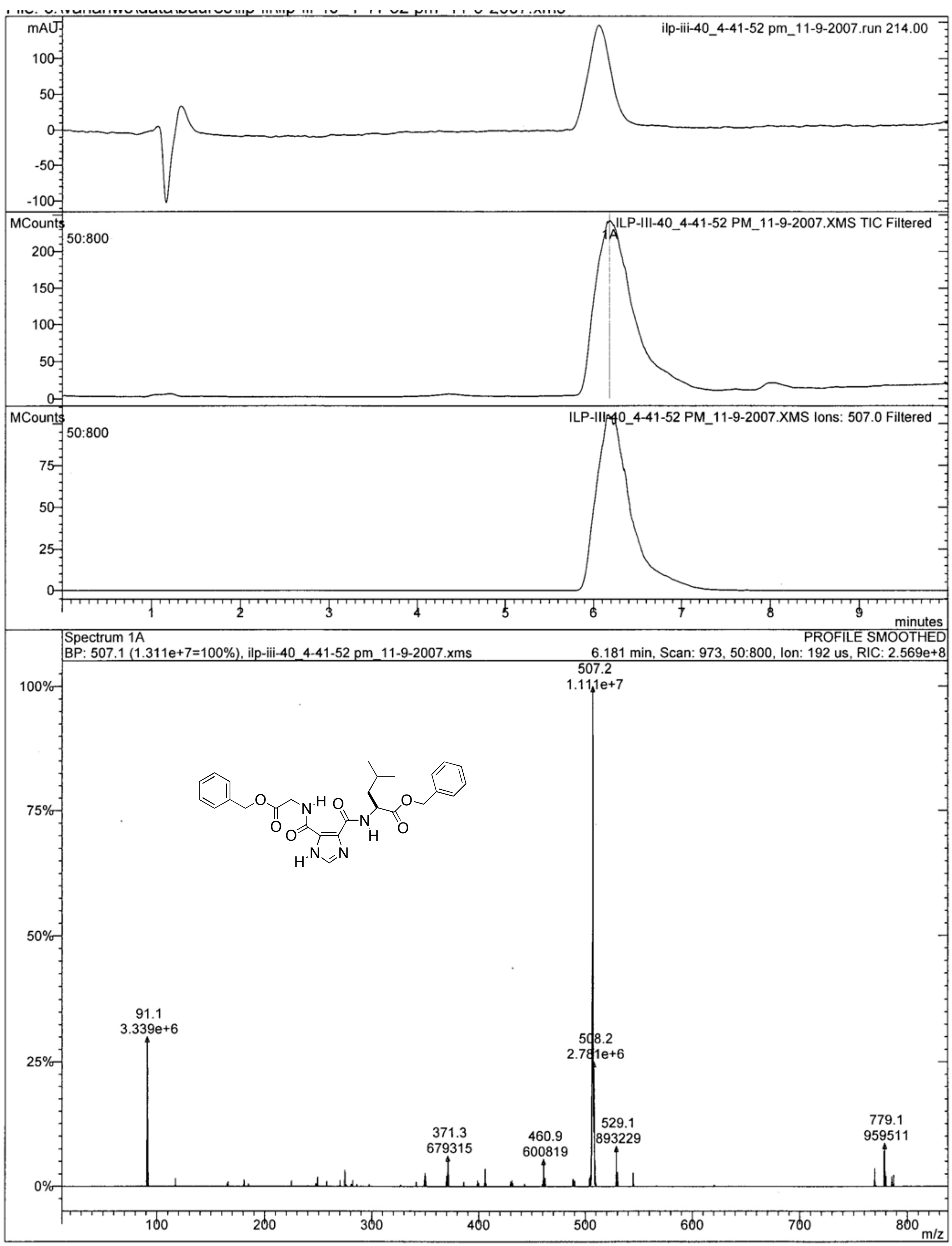

Figure S21. LC/MS data for **4{**21}.

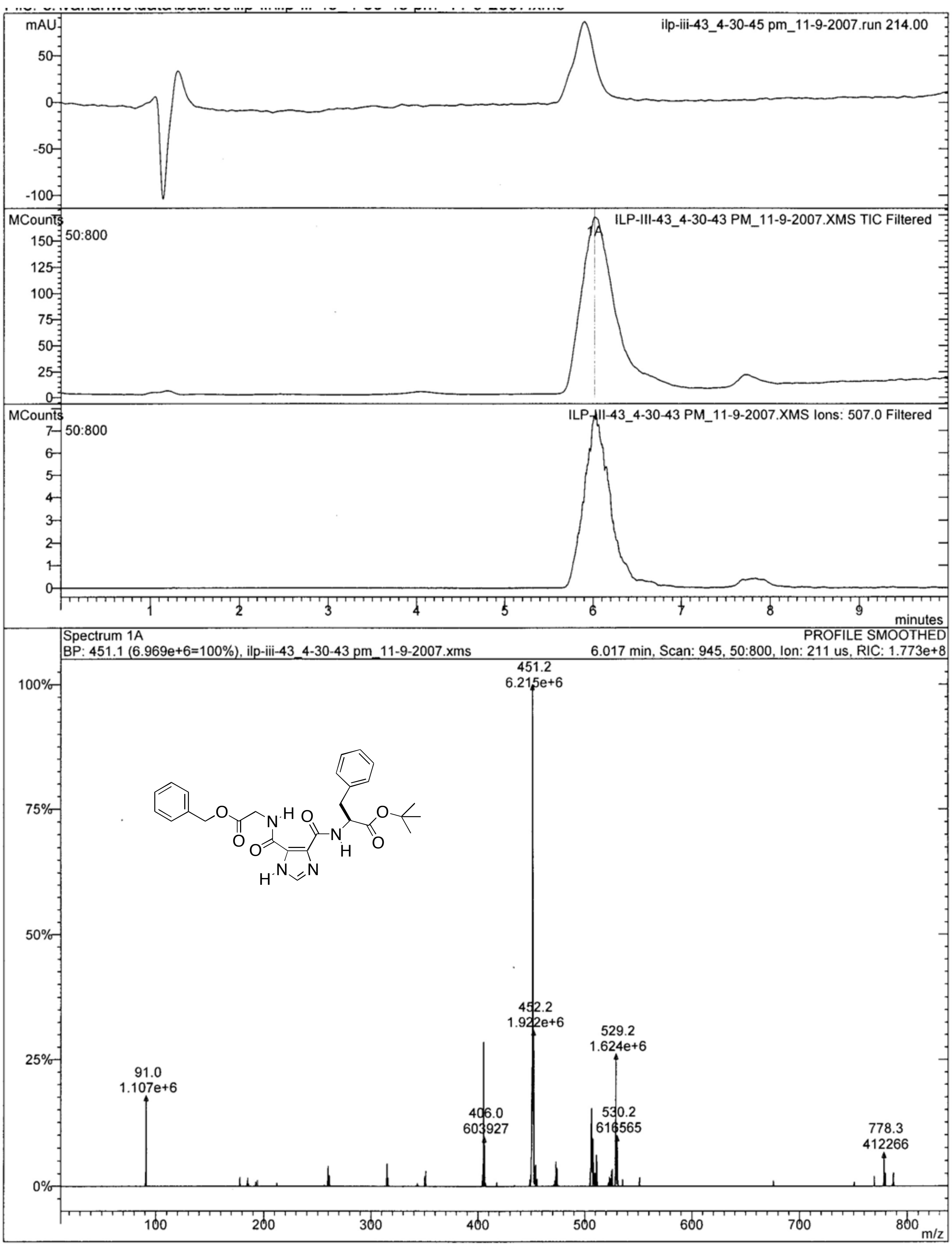

Figure S22. LC/MS data for **4{**22}.

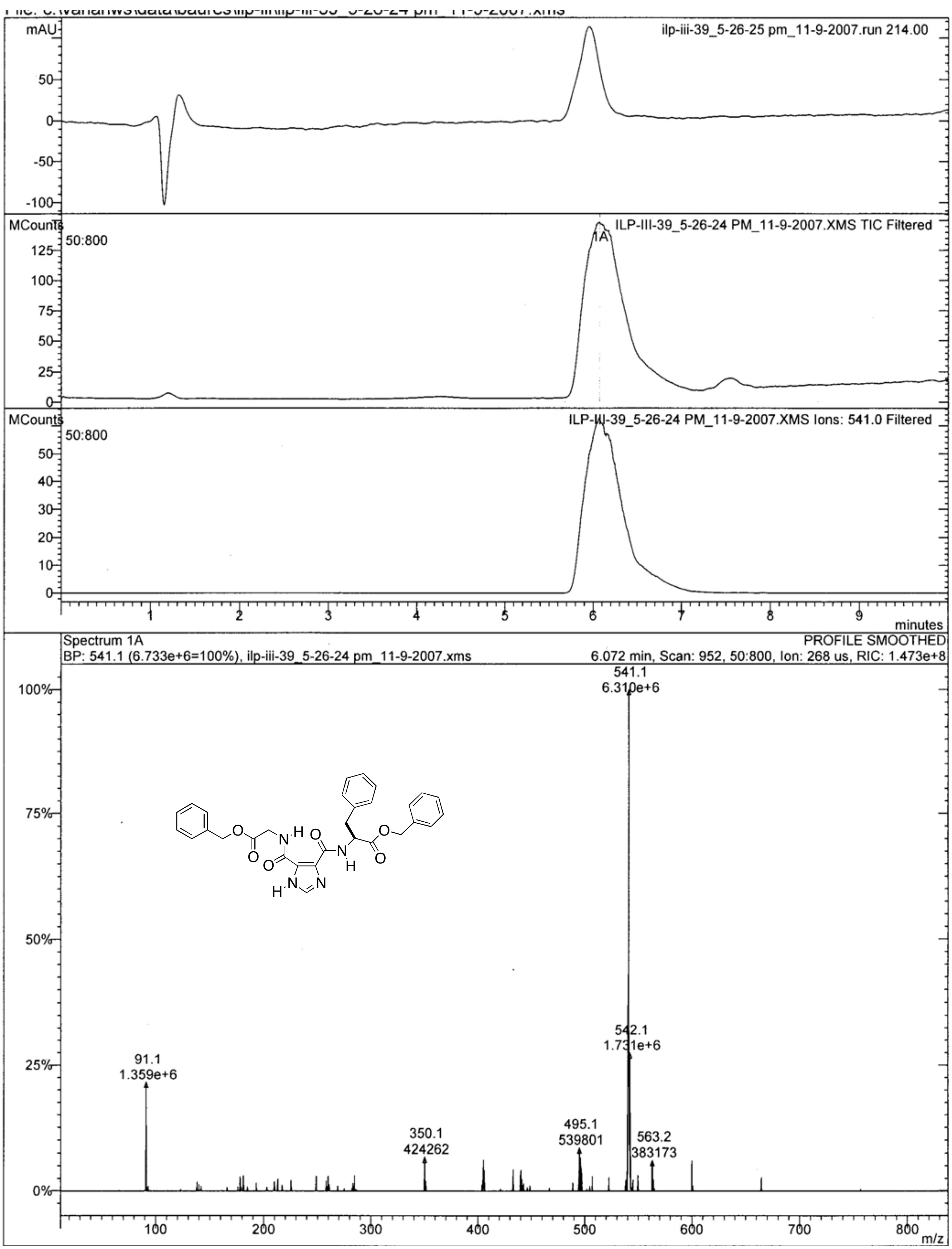

Figure S23. LC/MS data for **4{**23}.

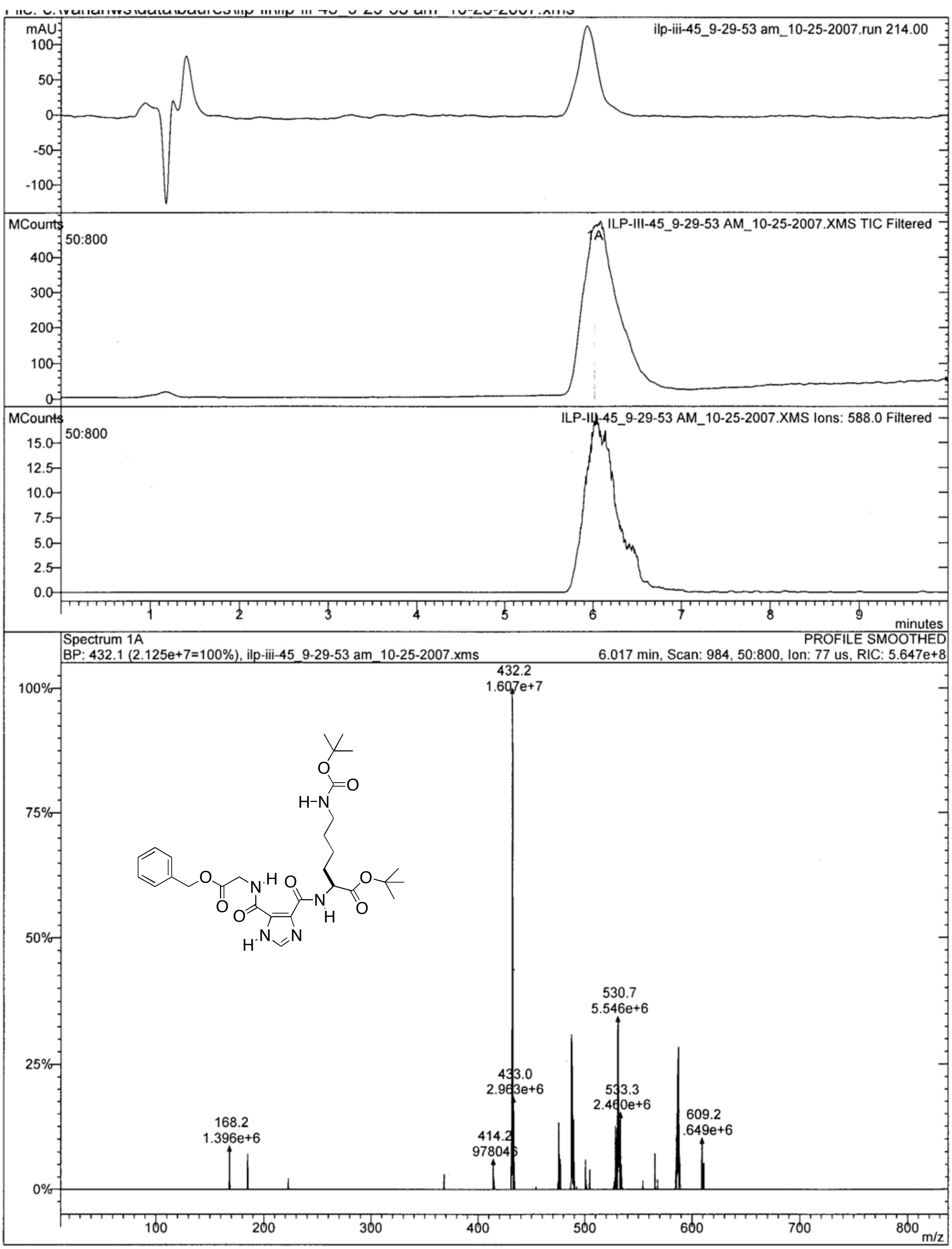

Figure S24. LC/MS data for **4{**24}.

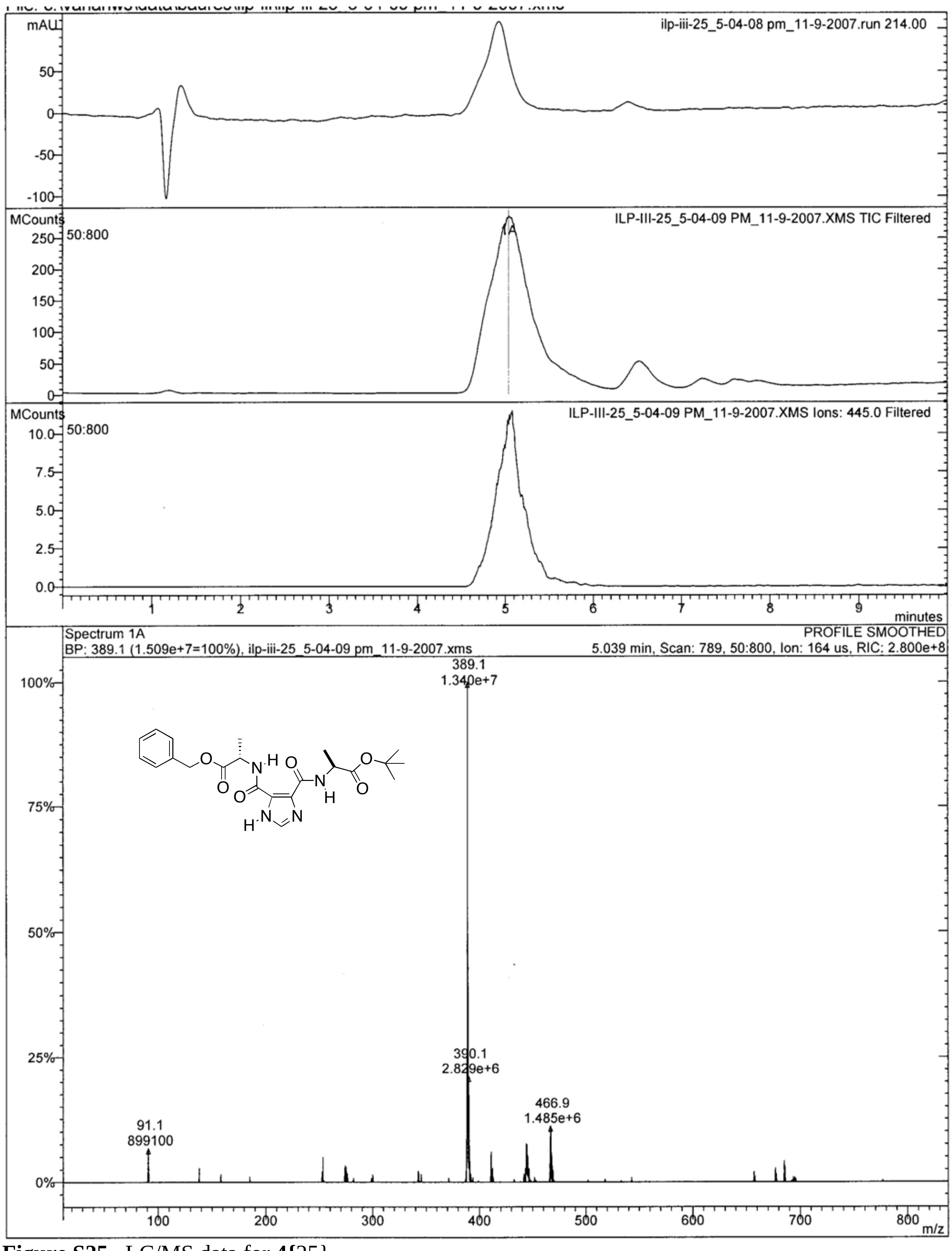

Figure S25. LC/MS data for **4{**25}.

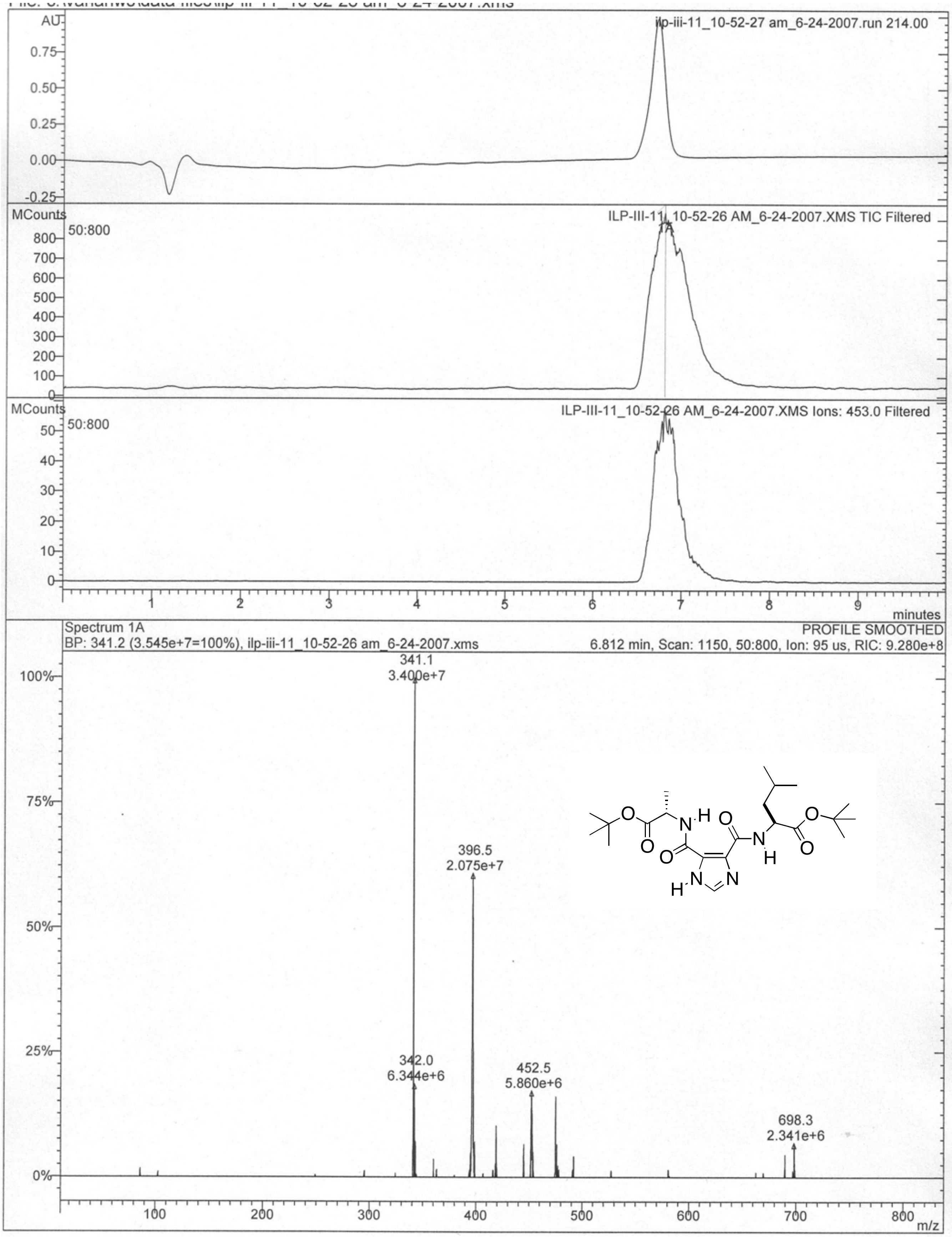

Figure S26. LC/MS data for **4{**26}.
