# Supplementary material for: Parallel Synthesis of a Library of Symmetrically- and Dissymmetrically-disubstituted Imidazole-4,5-dicarboxamides Bearing Amino Acid Esters
Source: Molecules. 2009 Jan 13;14(1):352–63. doi: 10.3390/molecules14010352 (PMC2635021; doi:10.3390/molecules14010352)
Supplement: Supplementary File 2 [file molecules-14-00352-s002.pdf]

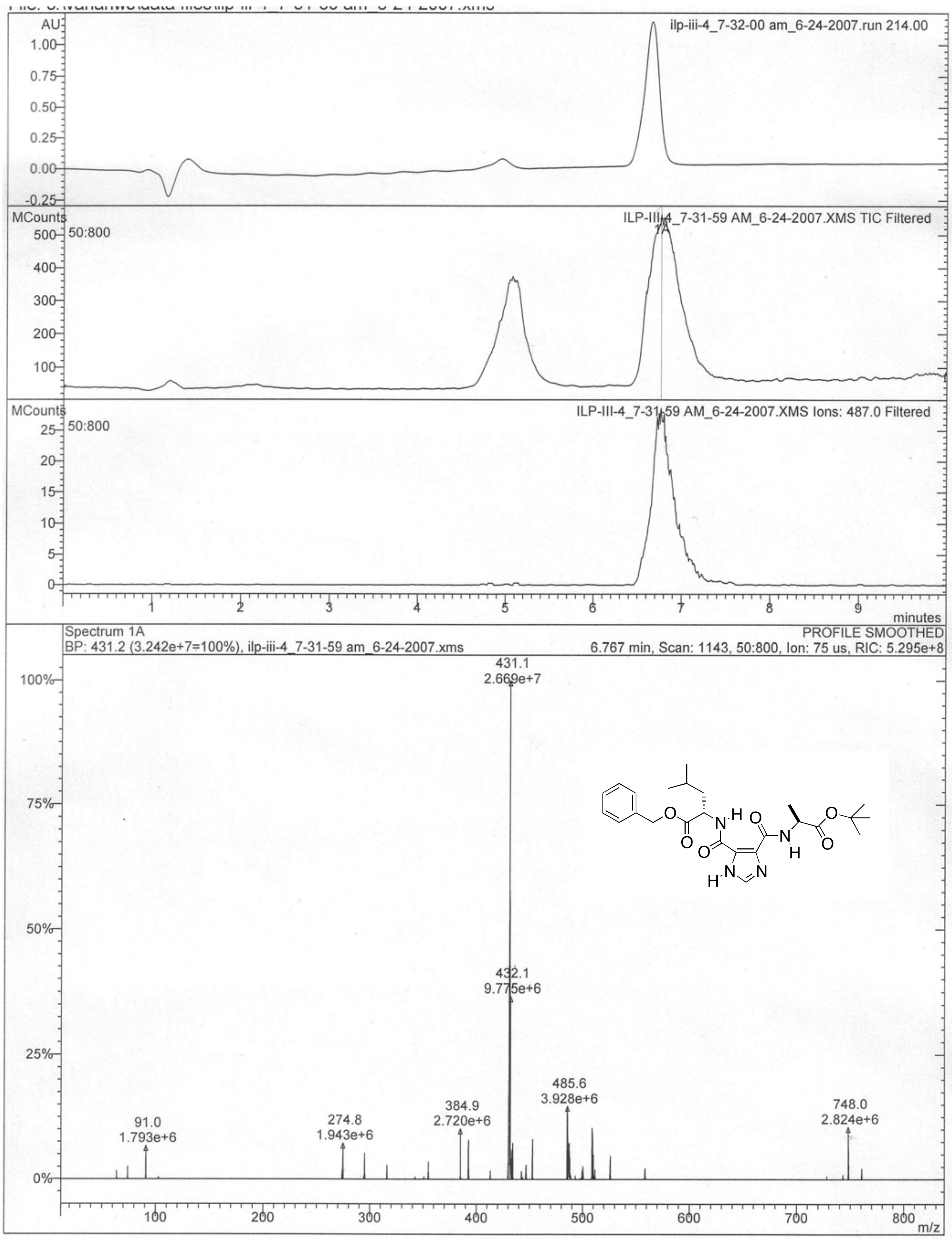

Figure S27. LC/MS data for **4{**27}.

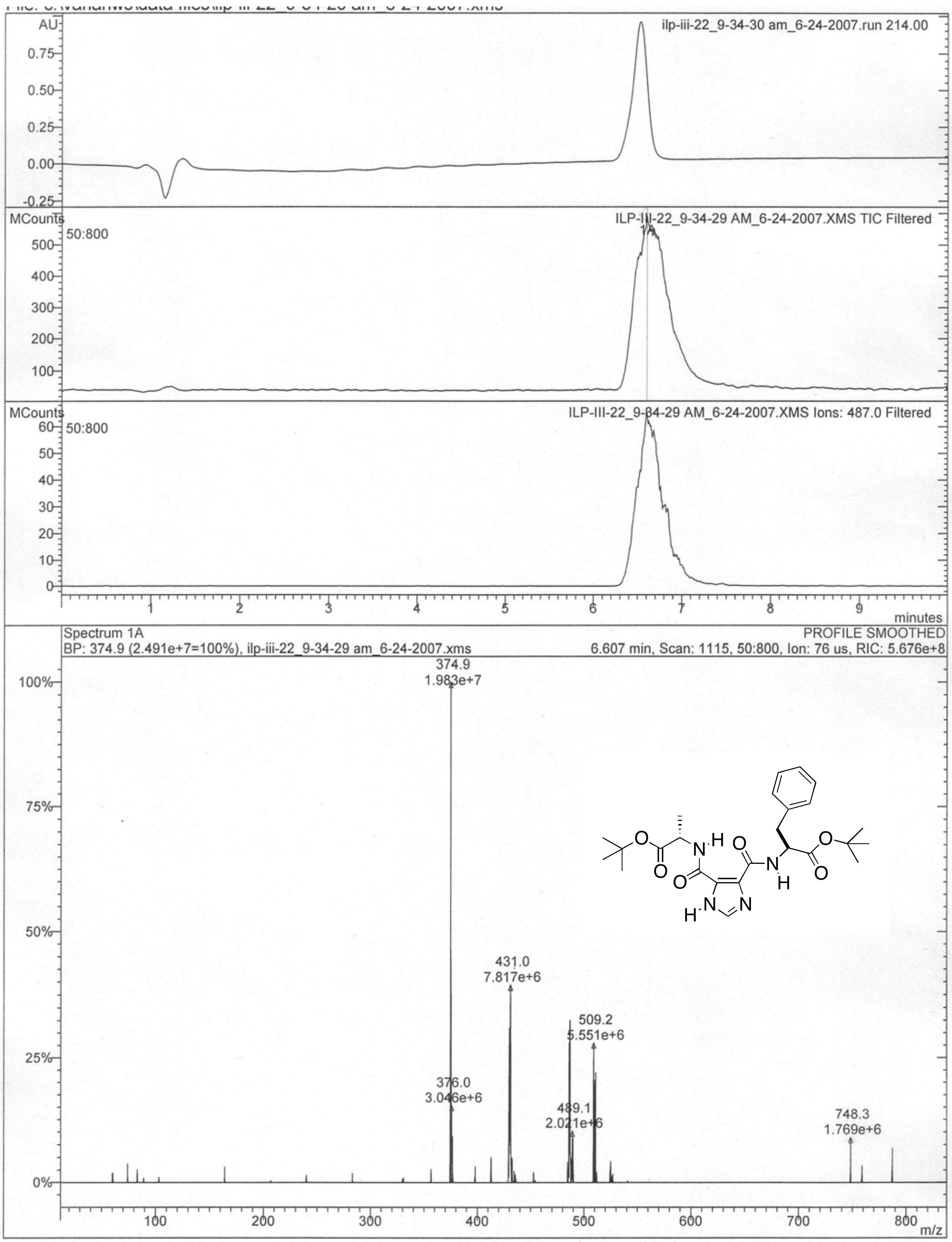

Figure S28. LC/MS data for **4{**28}.

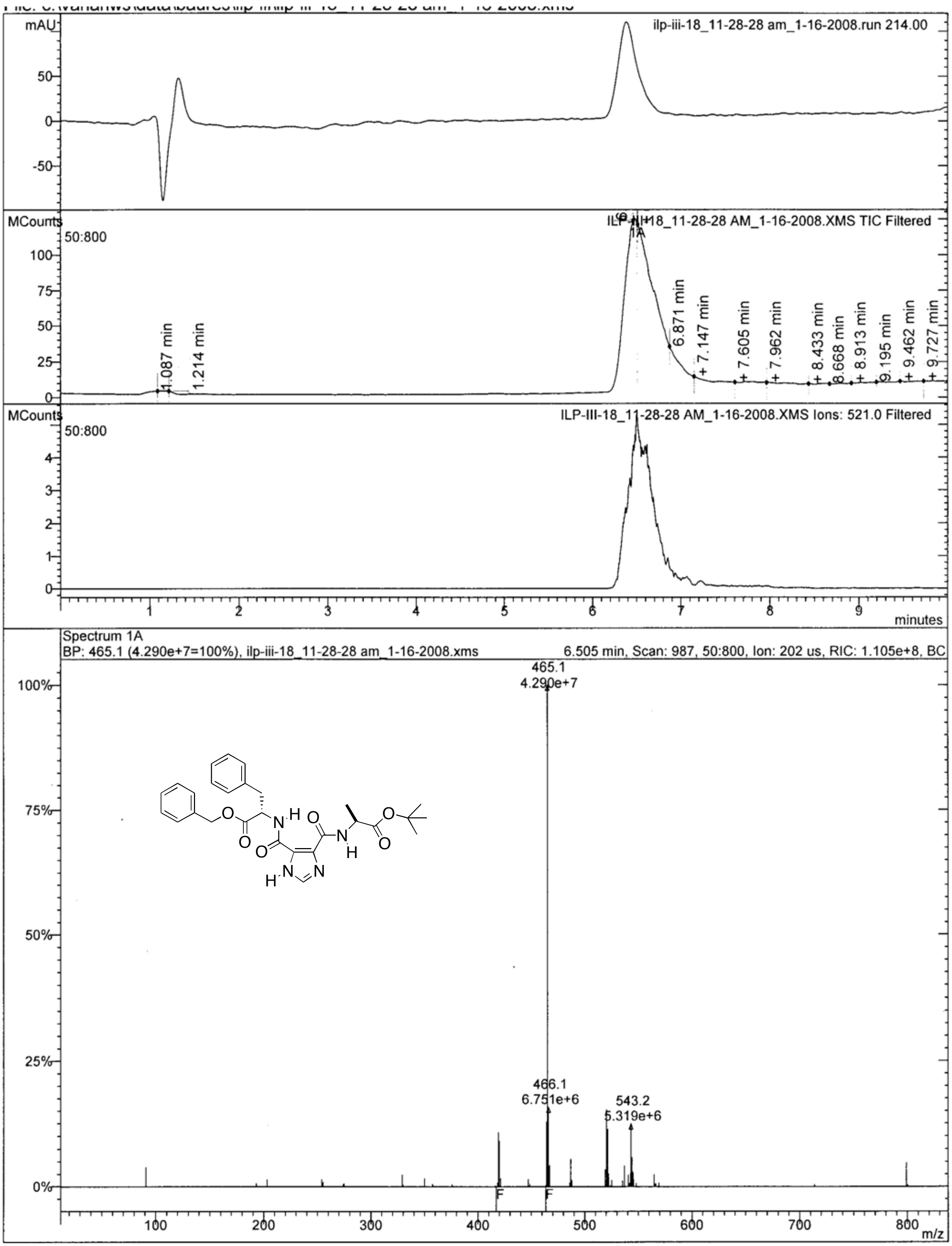

Figure S29. LC/MS data for **4{**29}.

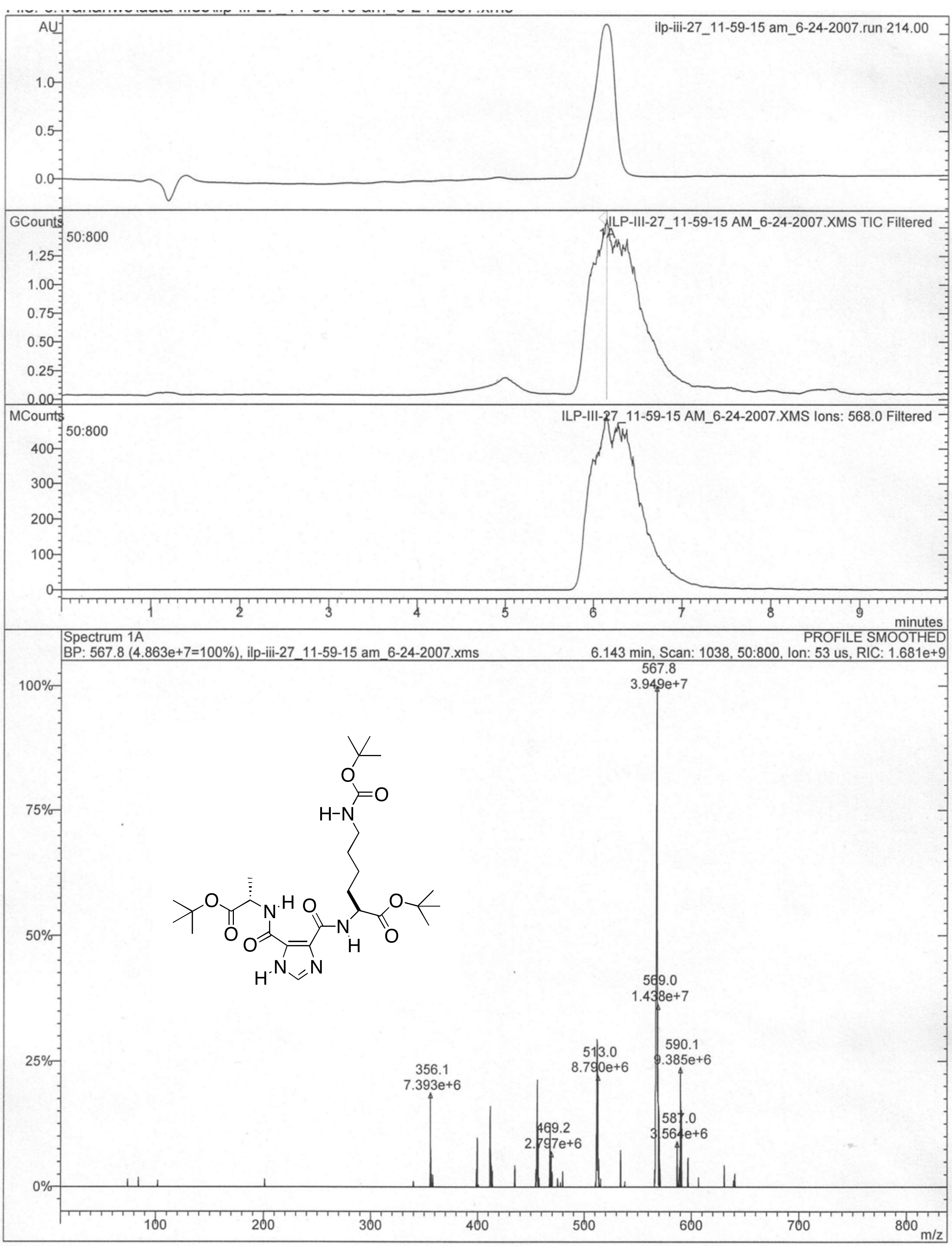

Figure S30. LC/MS data for **4{**30}.

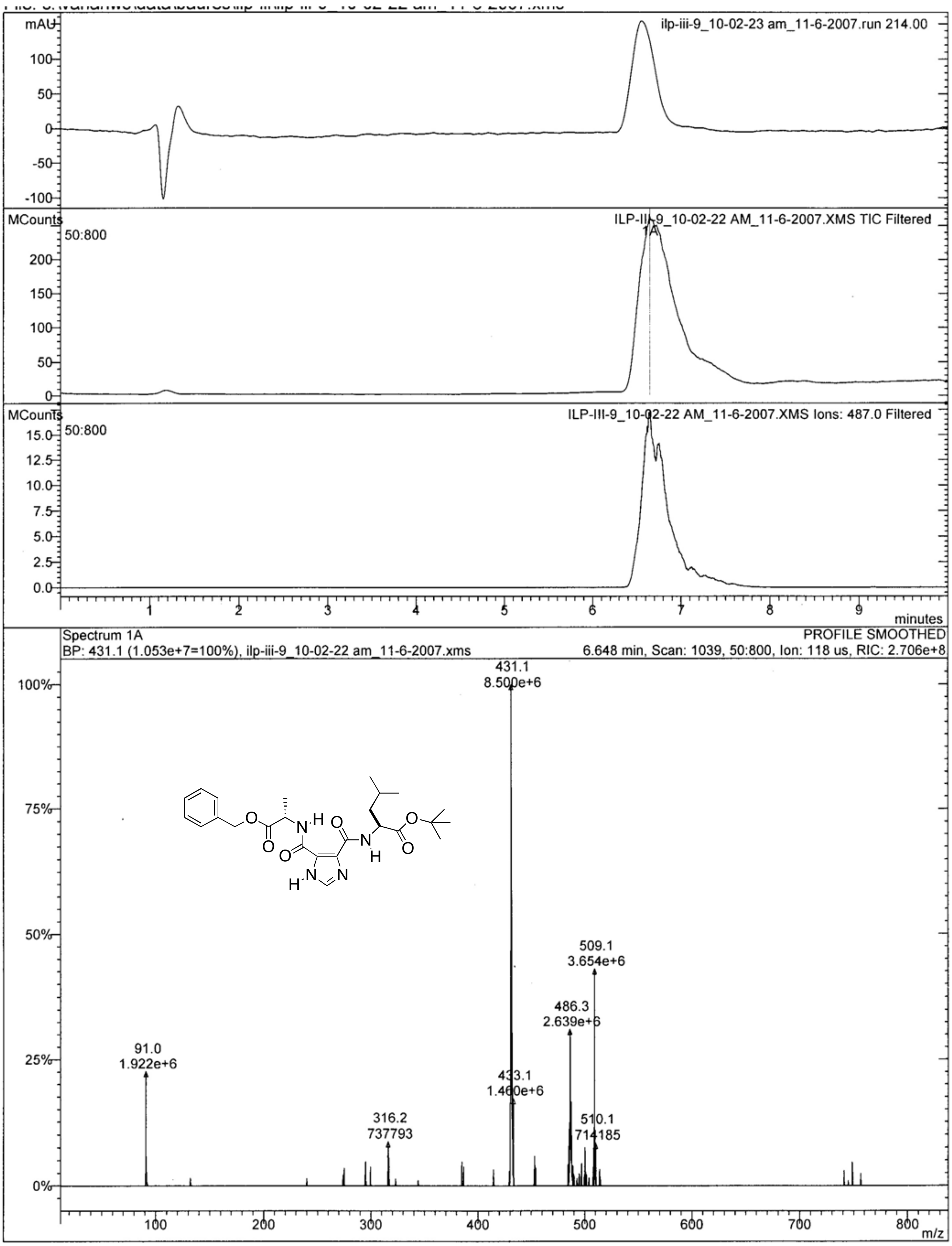

Figure S31. LC/MS data for **4{**31}.

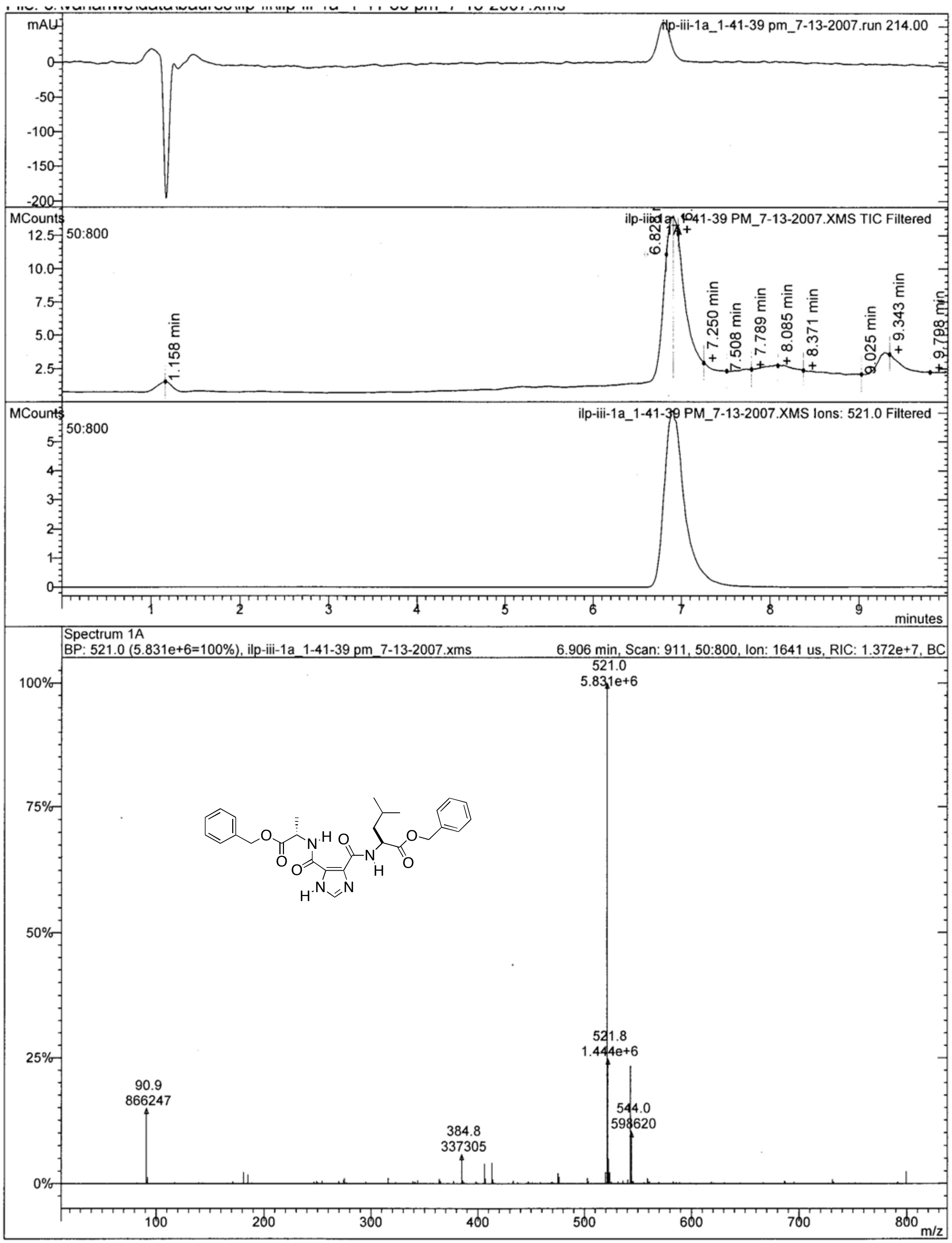

Figure S32. LC/MS data for **4{**32}.

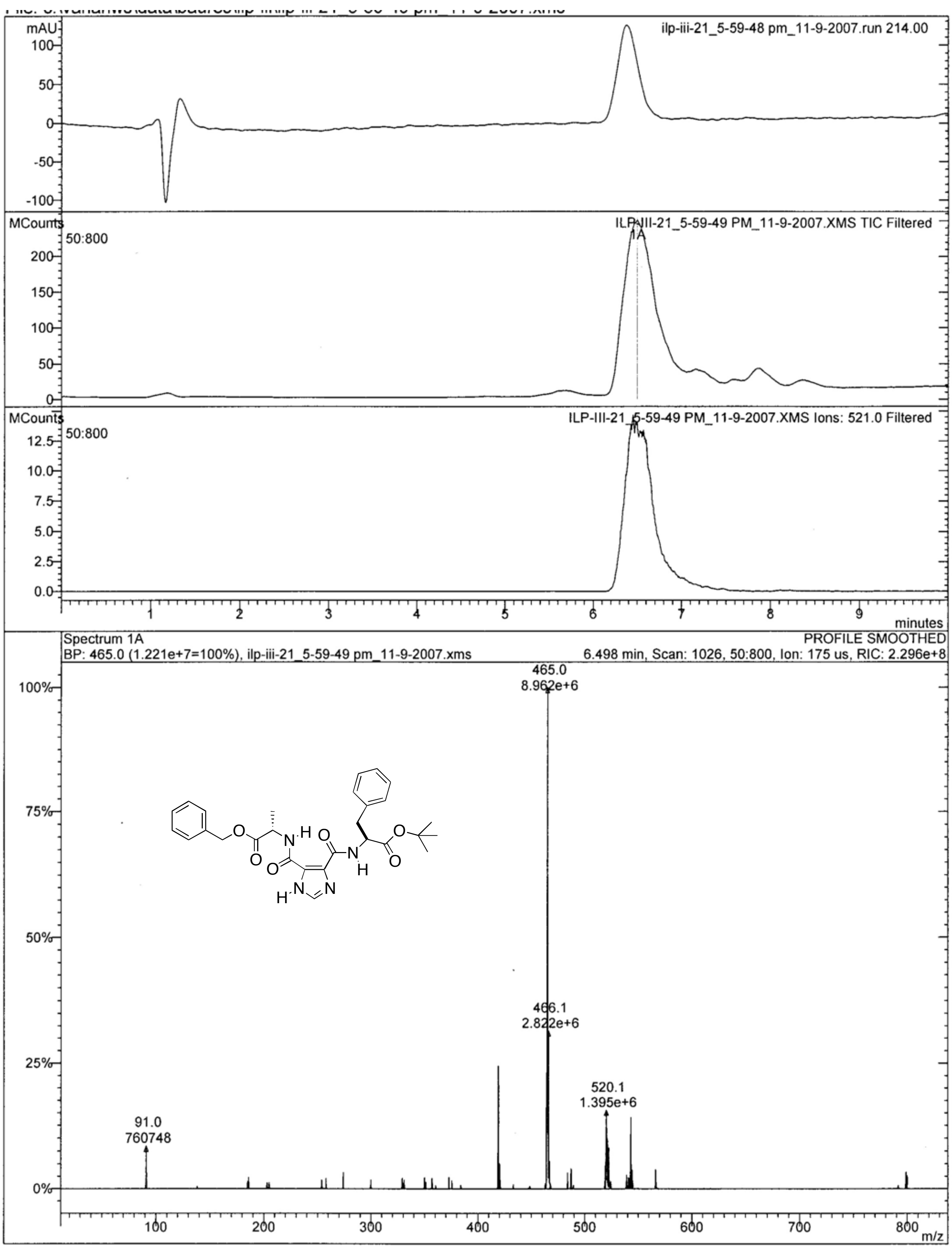

Figure S33. LC/MS data for **4{**33}.

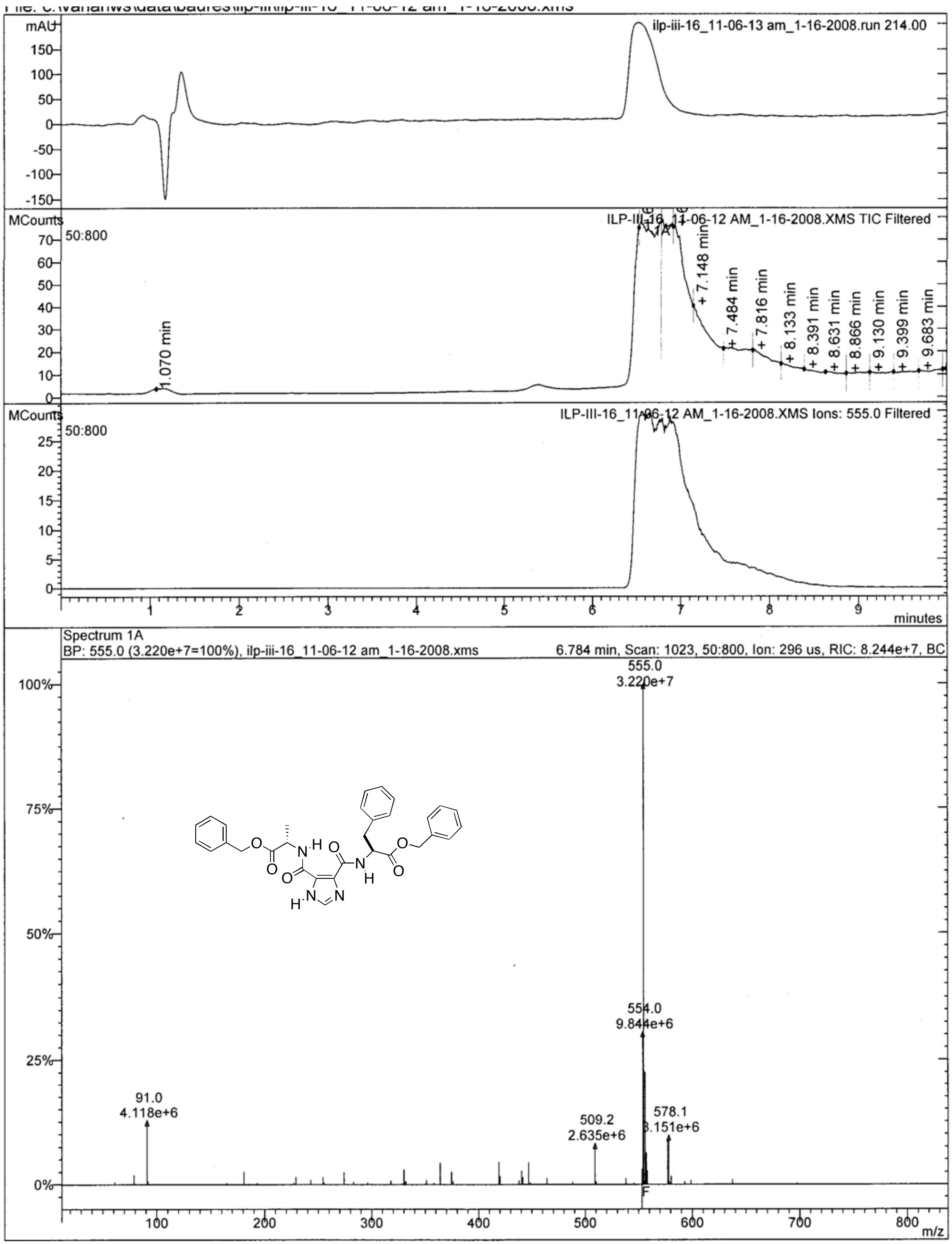

Figure S34. LC/MS data for **4{**34}.

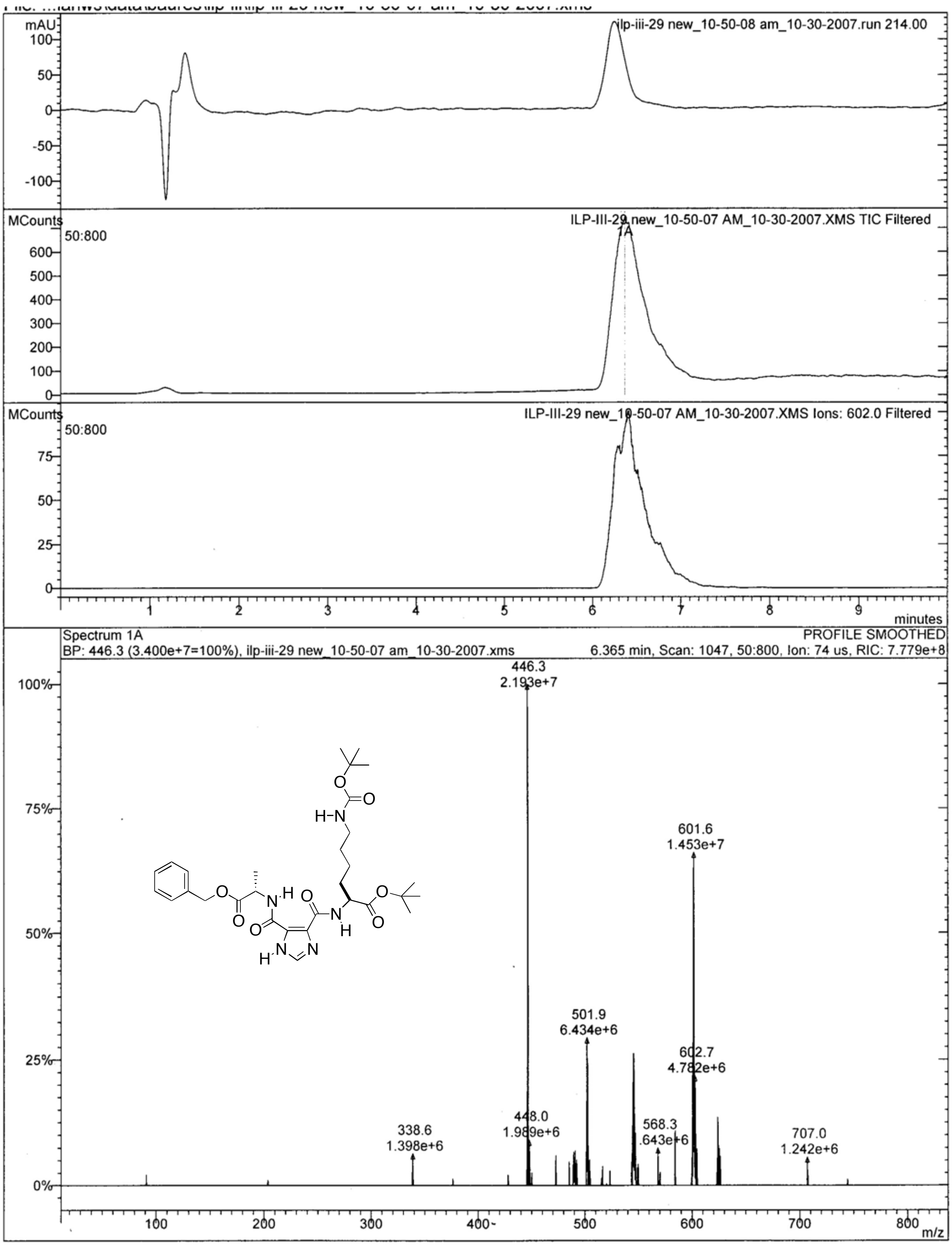

Figure S35. LC/MS data for **4{**35}.

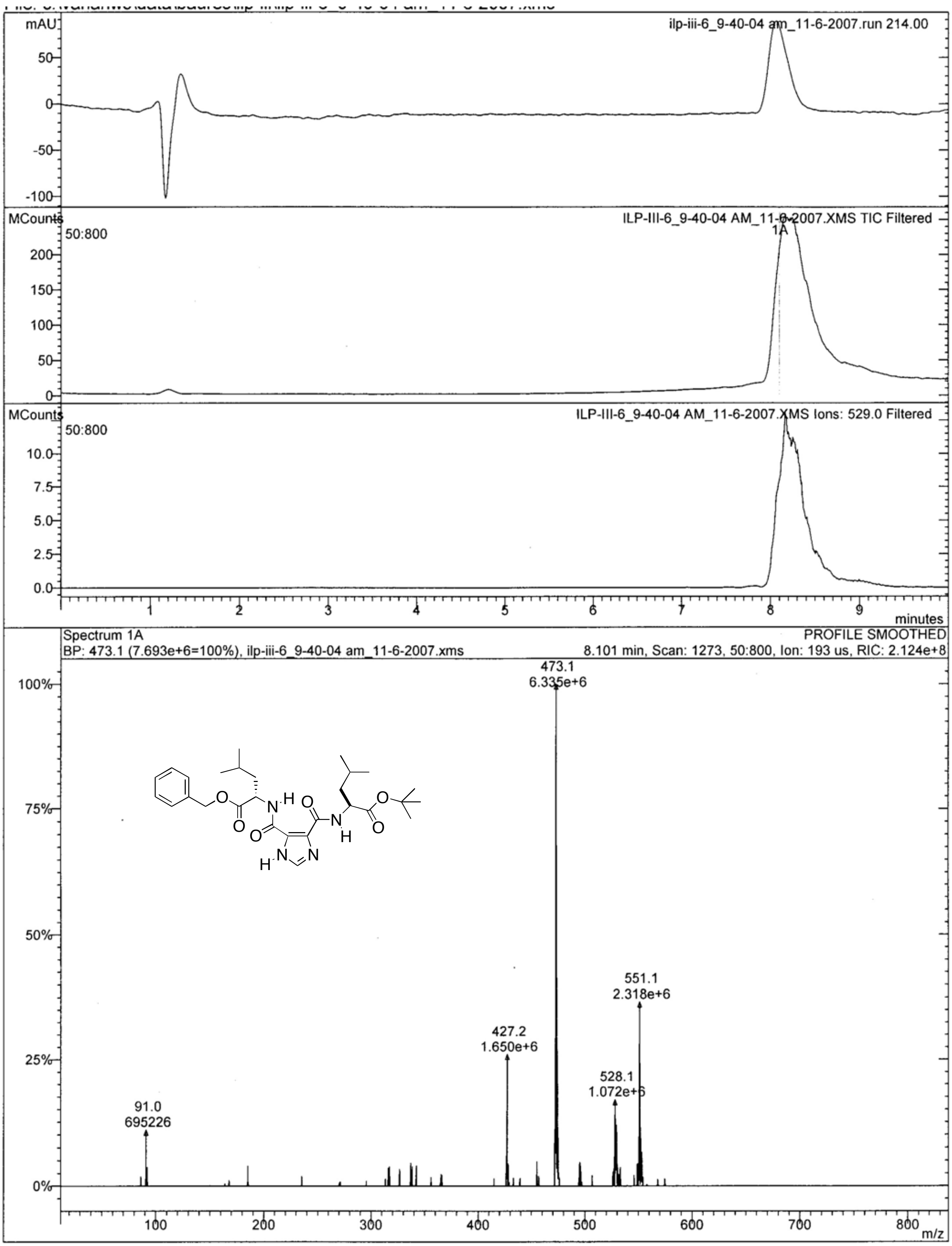

Figure S36. LC/MS data for **4{**36}.

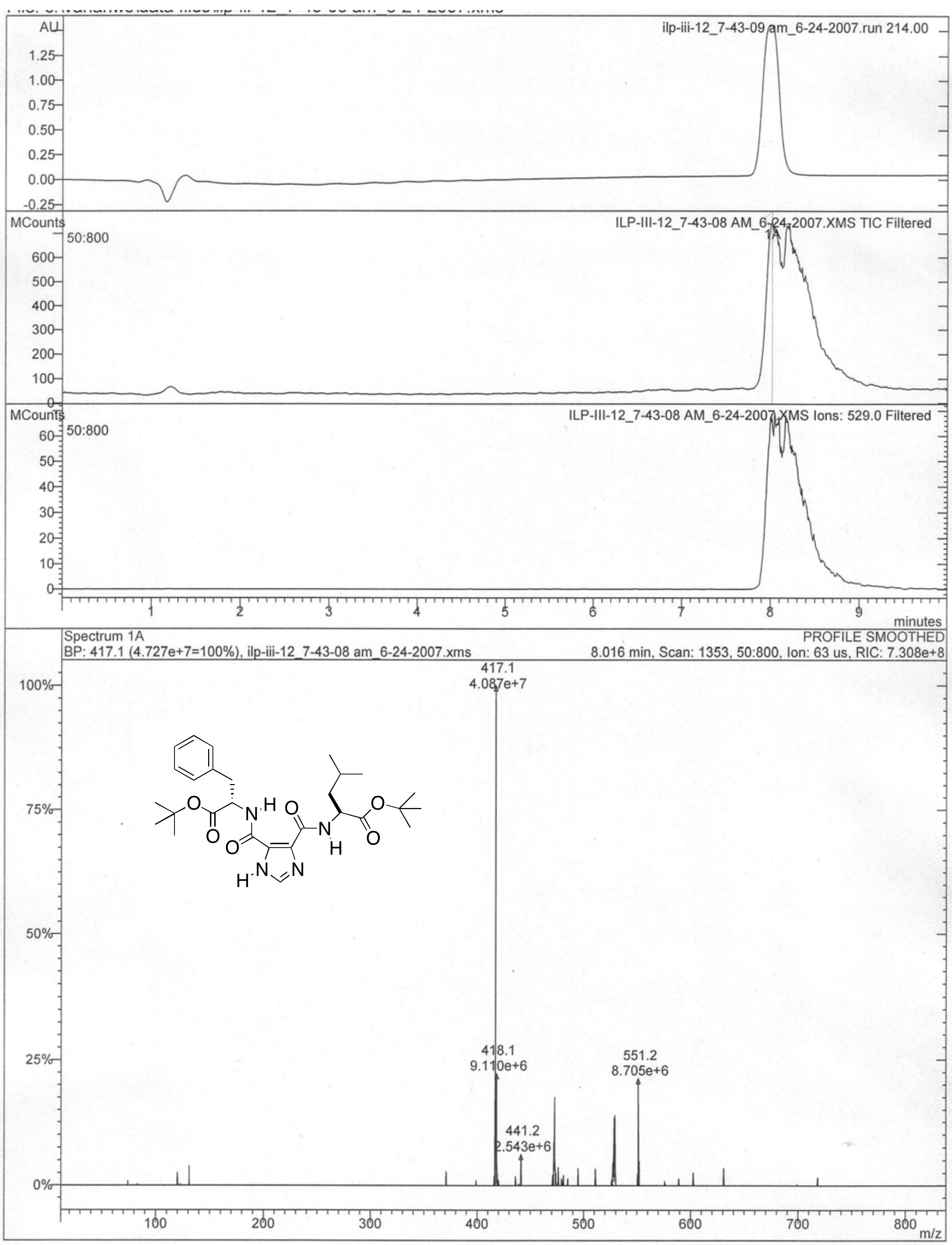

Figure S37. LC/MS data for **4{**37}.

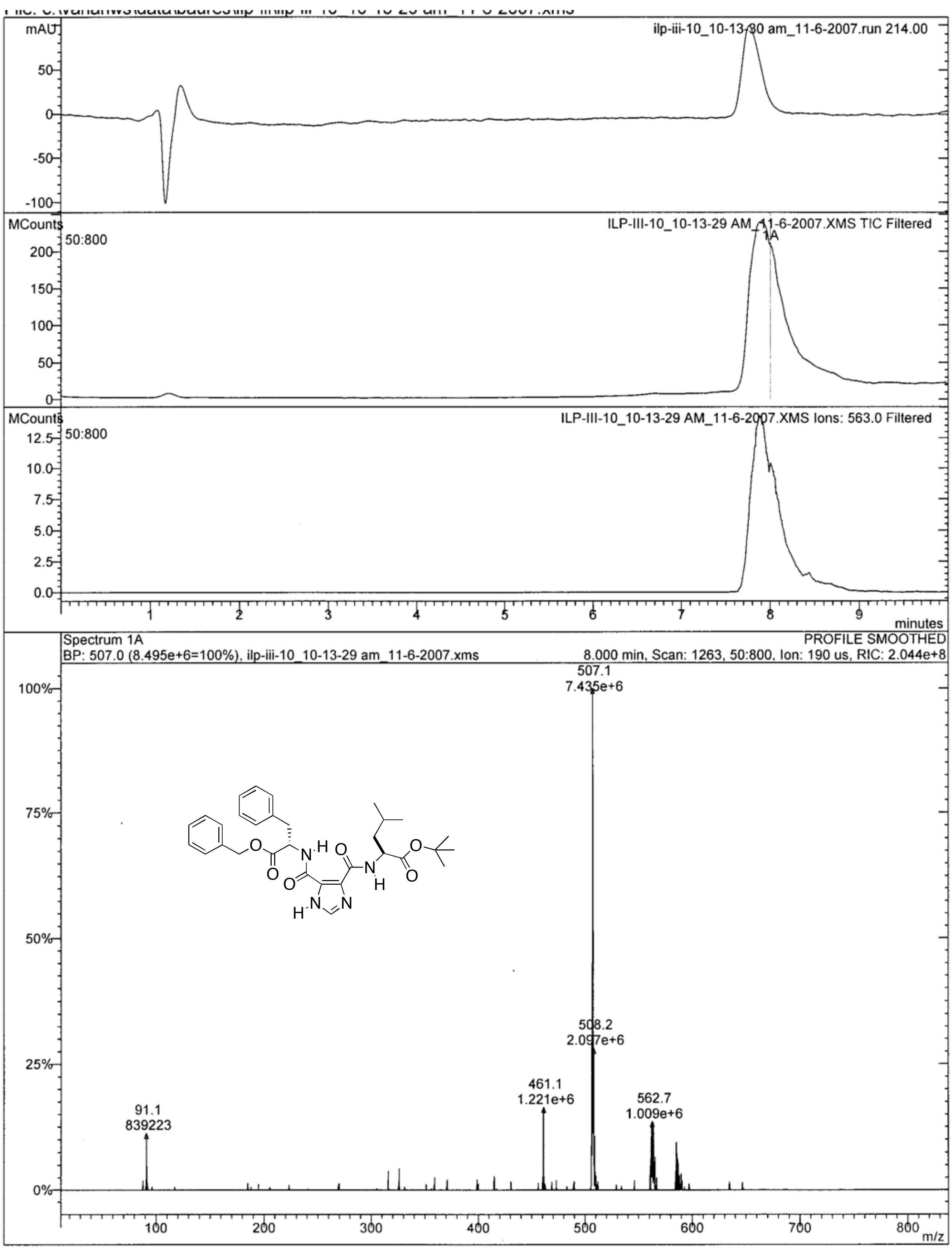

Figure S38. LC/MS data for **4{**38}.

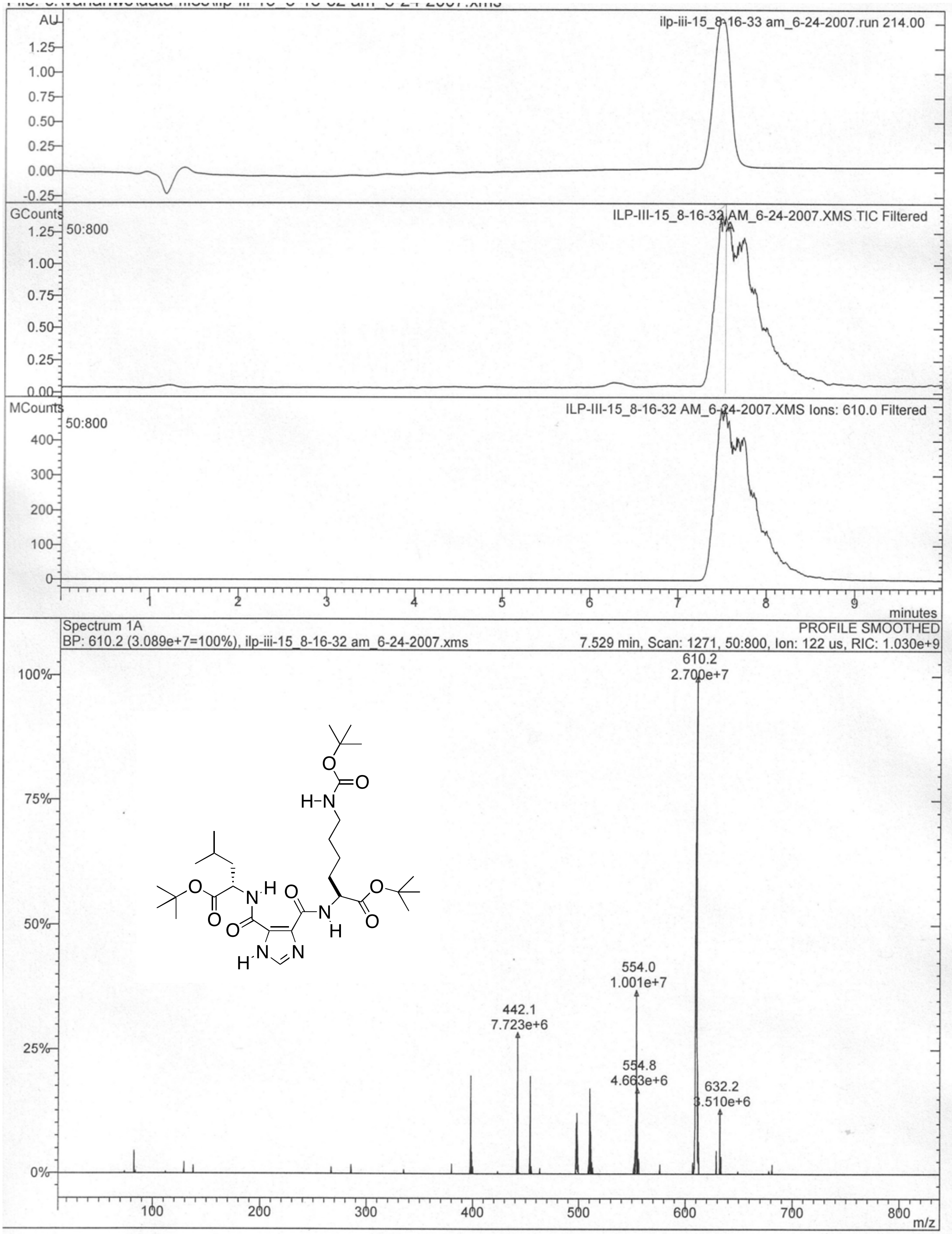

Figure S39. LC/MS data for **4{**39}.

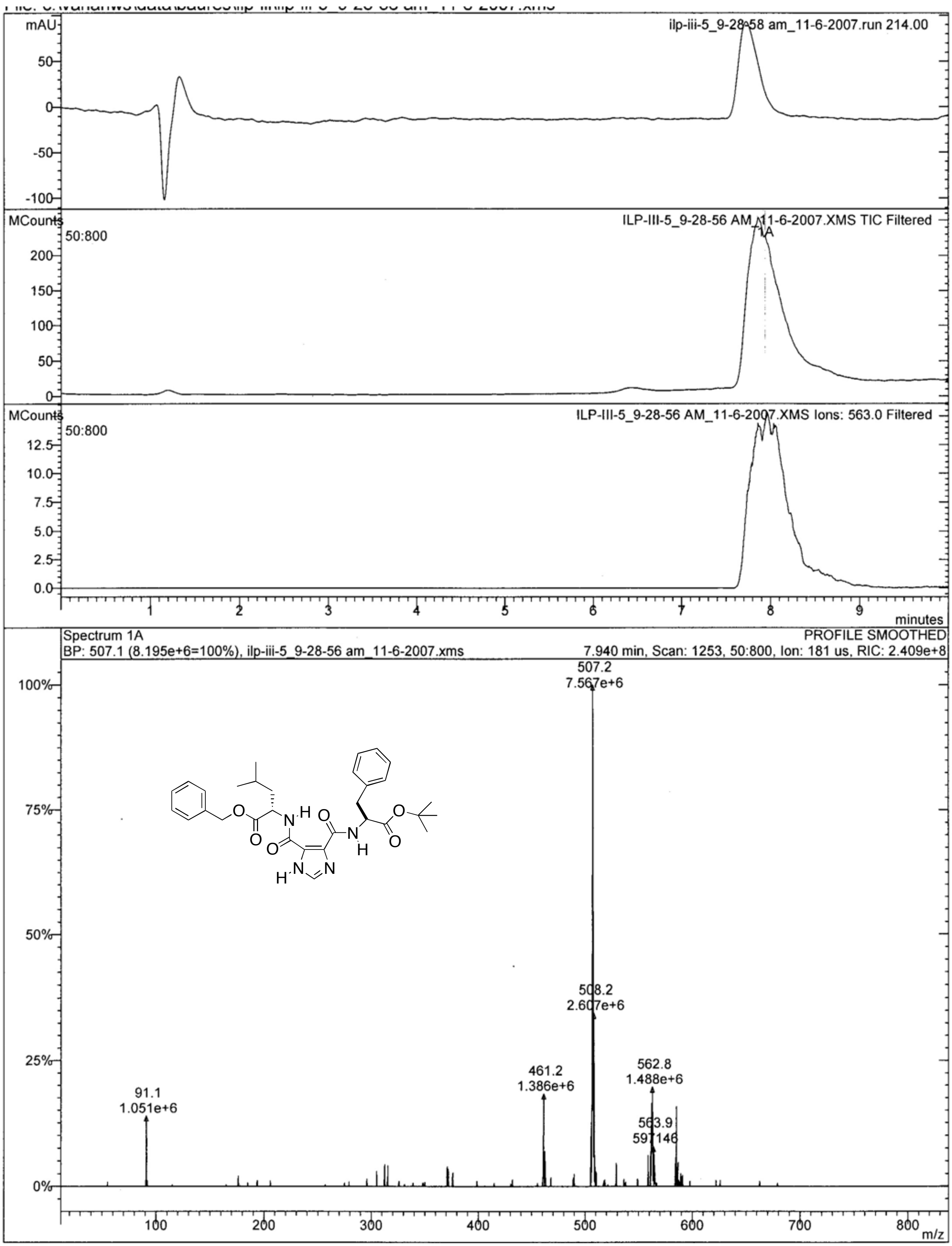

Figure S40. LC/MS data for **4{**40}.

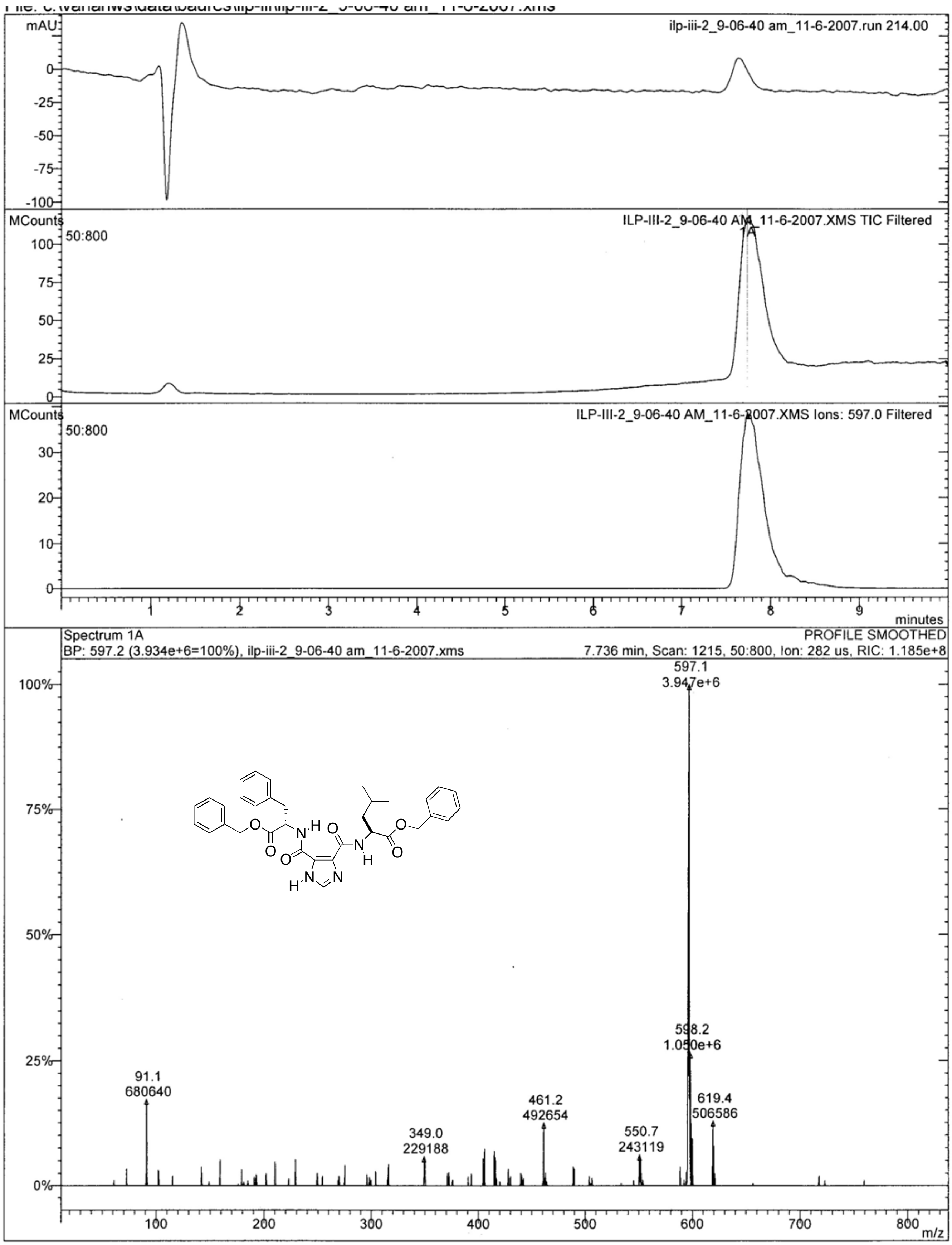

Figure S41. LC/MS data for **4{**41}.

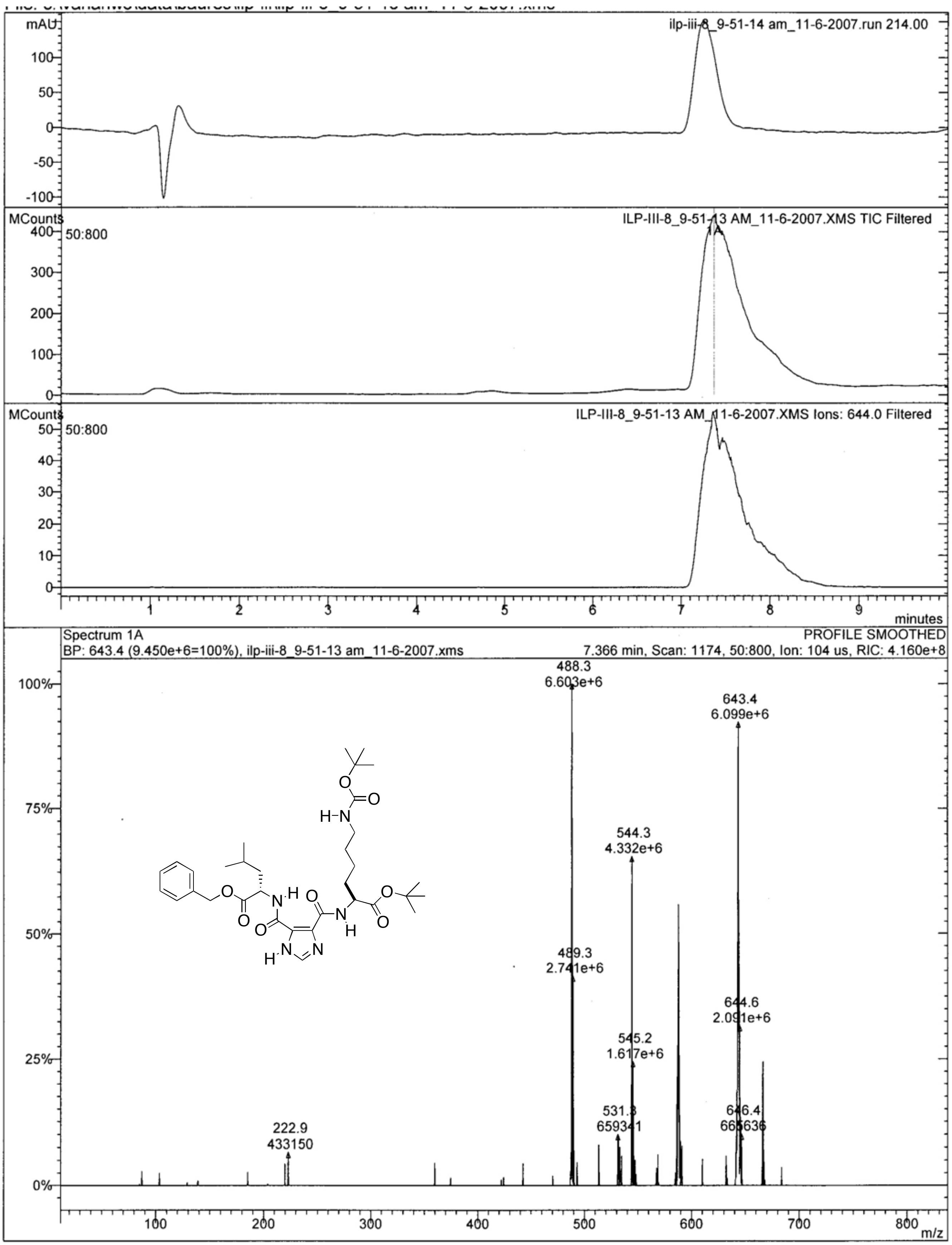

Figure S42. LC/MS data for **4{**42}.

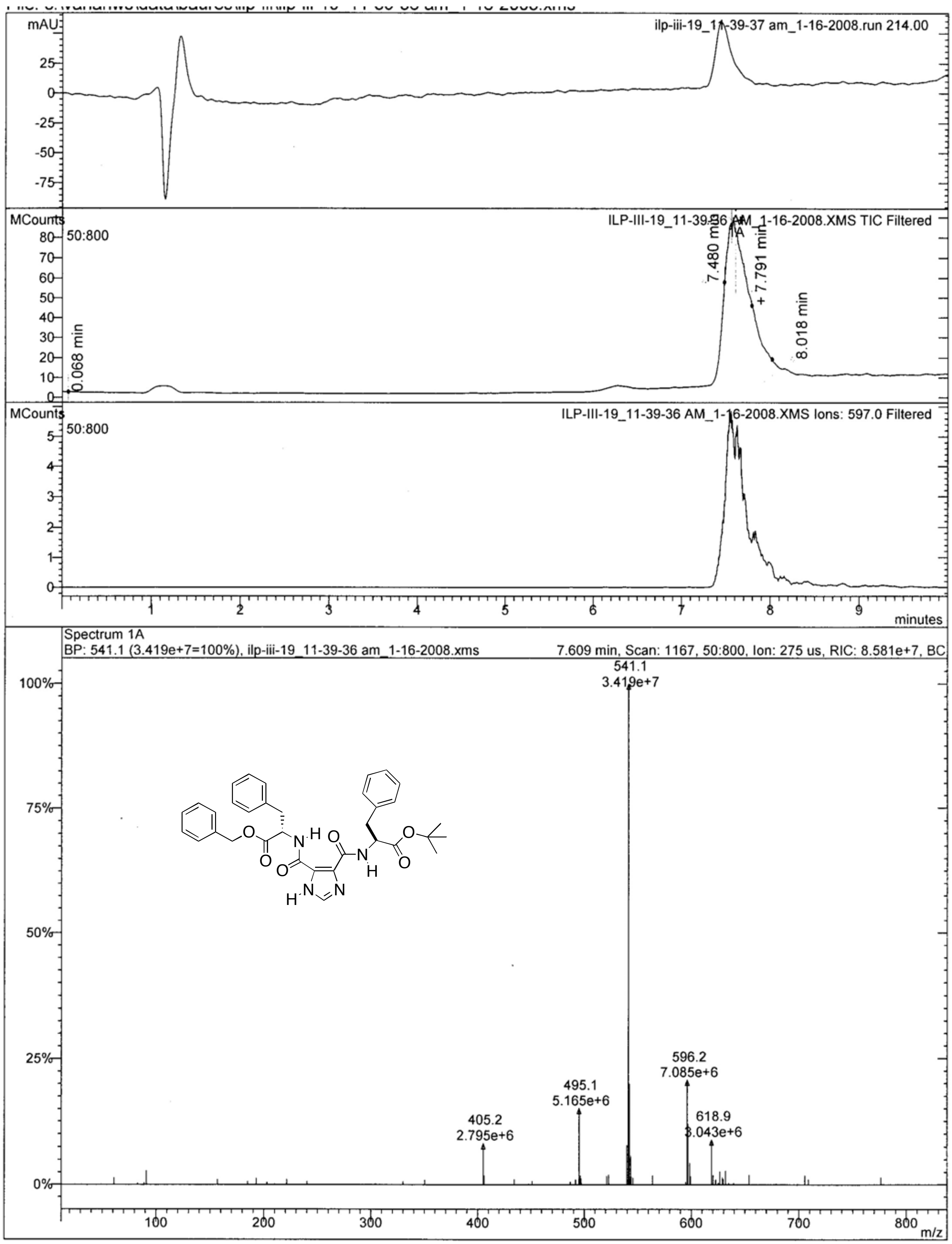

Figure S43. LC/MS data for **4{**43}.

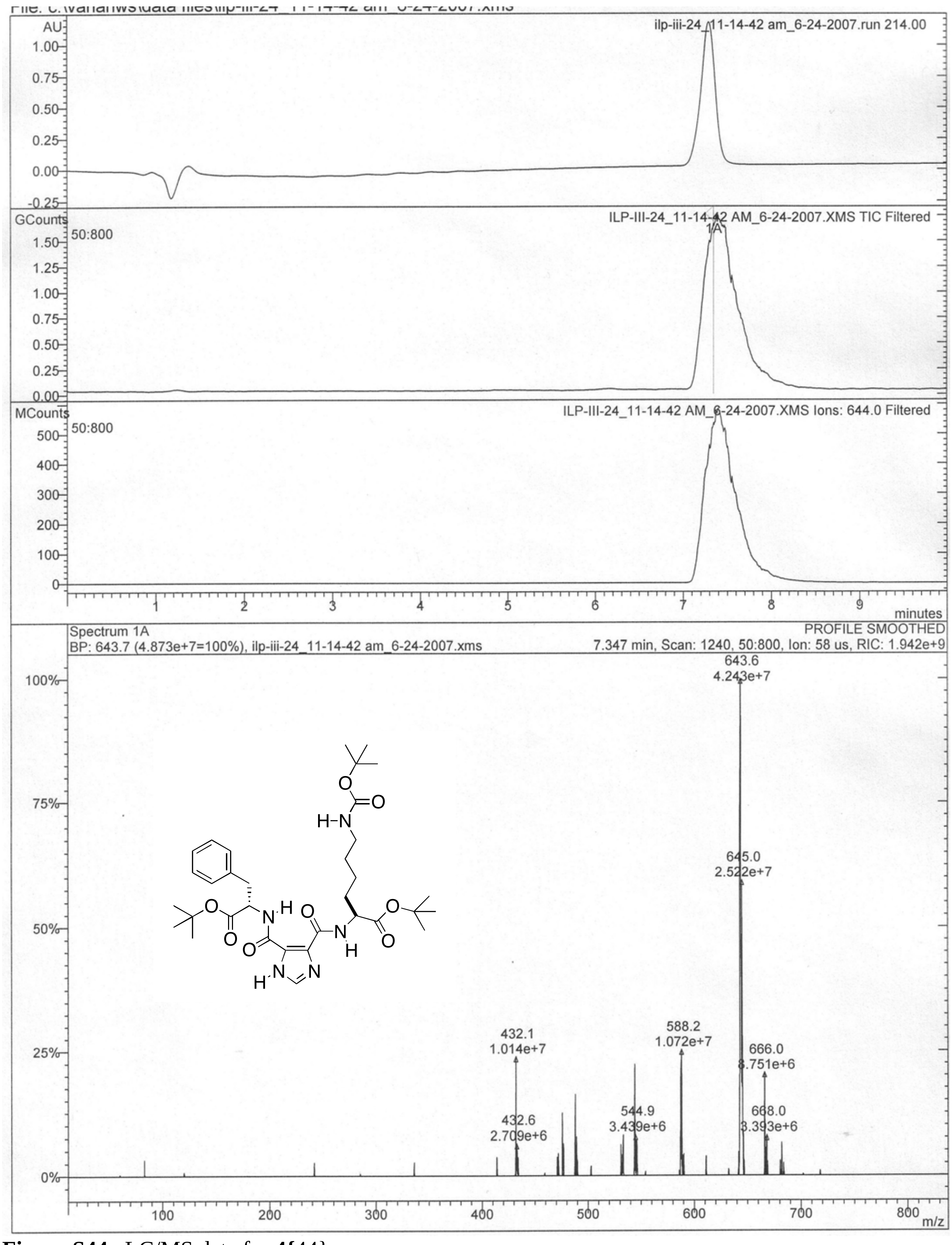

Figure S44. LC/MS data for **4{**44}.

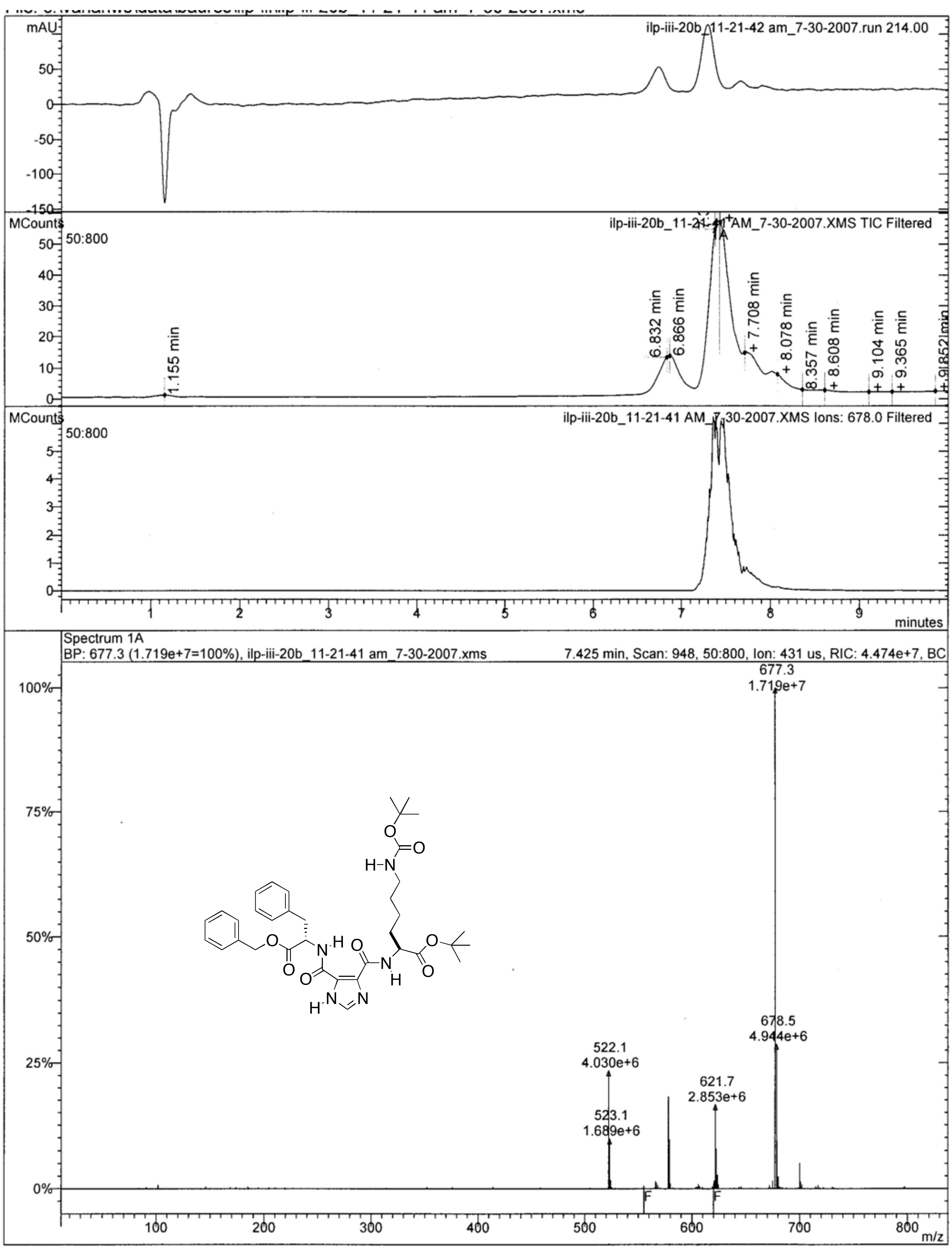

Figure S45. LC/MS data for **4{**45}.

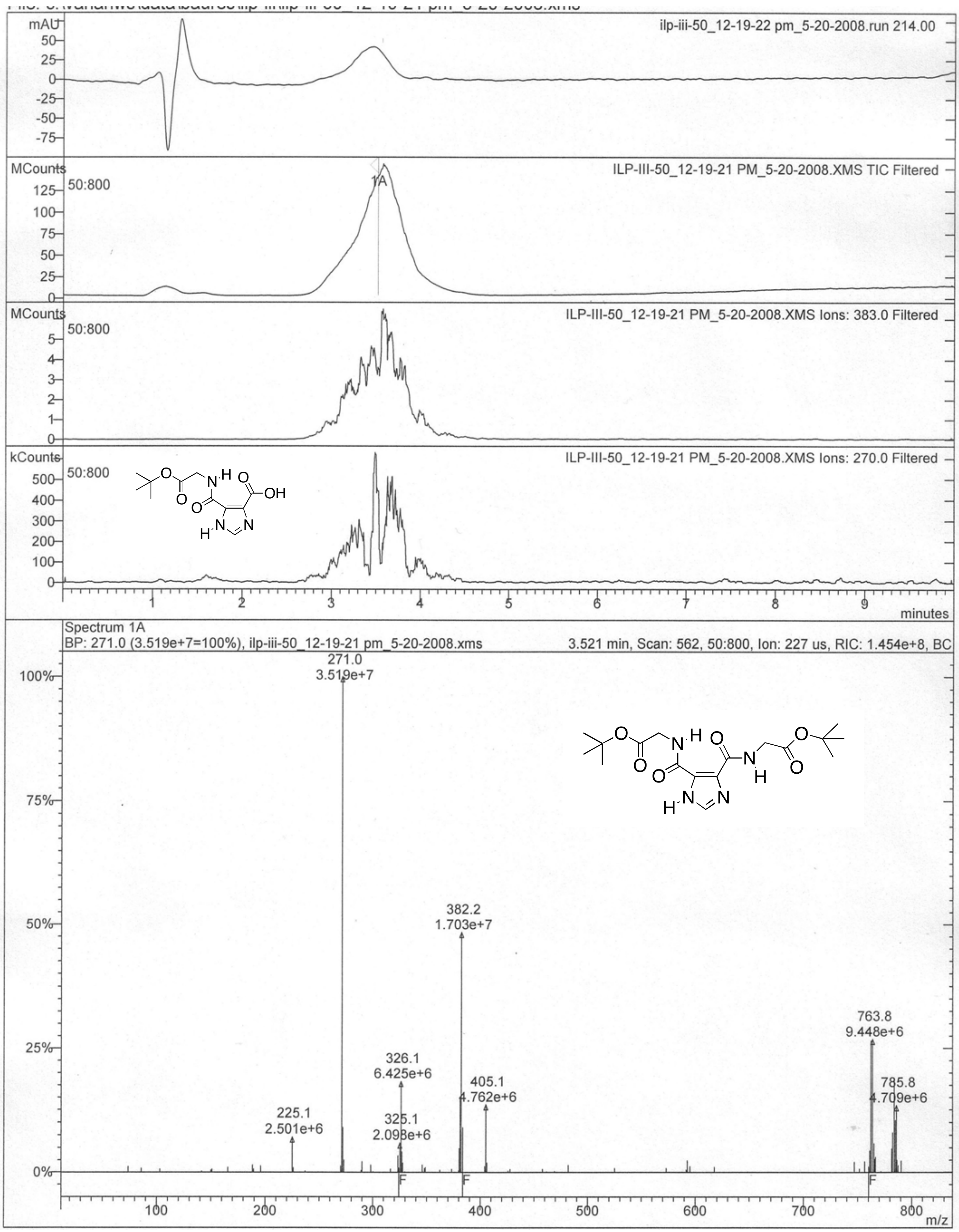

Figure S46. LC/MS data for the crude reaction to yield **4{**1**}**.
