# Supplementary material for: Parallel Synthesis of a Library of Symmetrically- and Dissymmetrically-disubstituted Imidazole-4,5-dicarboxamides Bearing Amino Acid Esters
Source: Molecules. 2009 Jan 13;14(1):352–63. doi: 10.3390/molecules14010352 (PMC2635021; doi:10.3390/molecules14010352)
Supplement: Supplementary File 3 [file molecules-14-00352-s003.pdf]

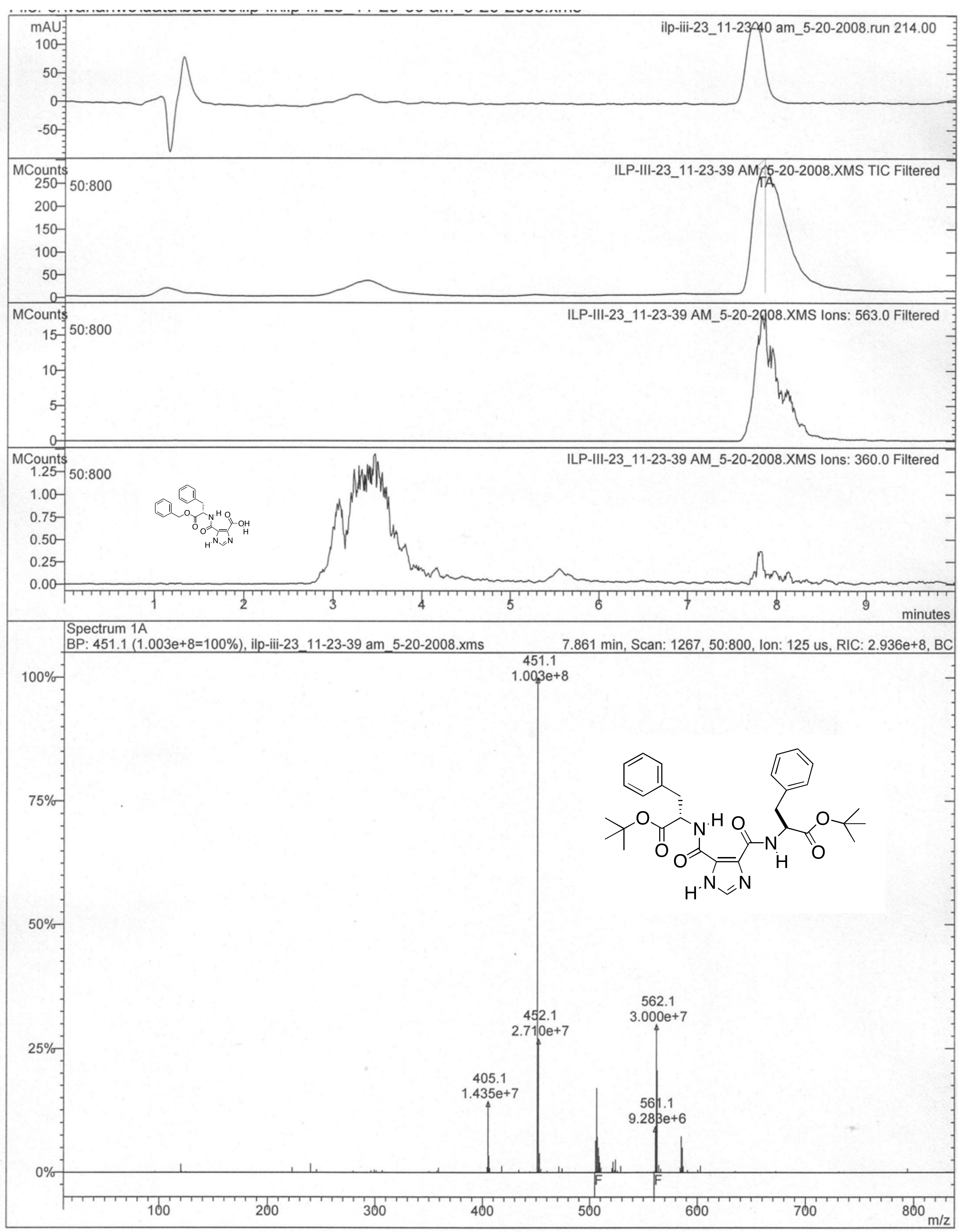

Figure S47. LC/MS data for the crude reaction to yield **4{**7}.

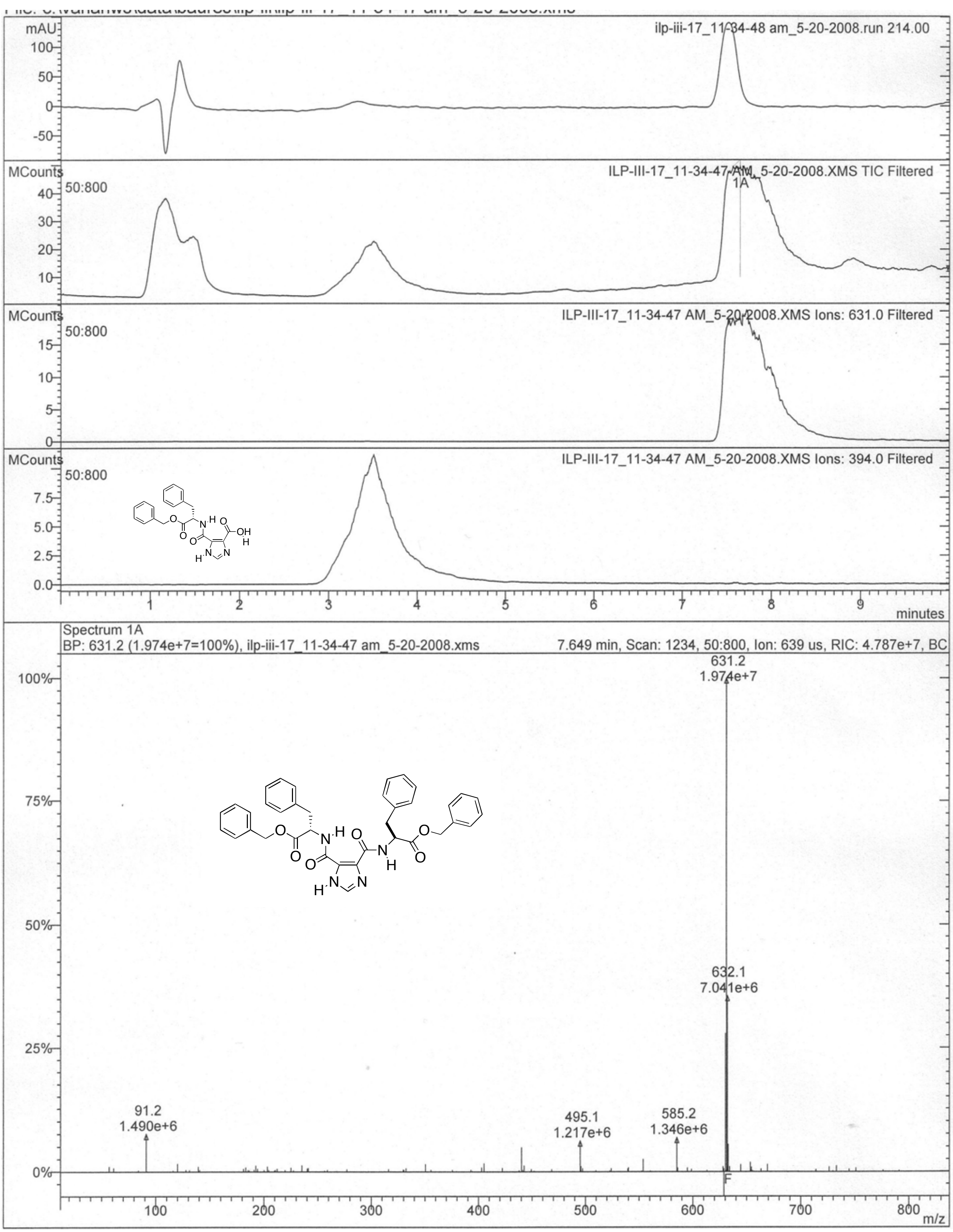

Figure S48. LC/MS data for the crude reaction to yield **4{**8**}**.

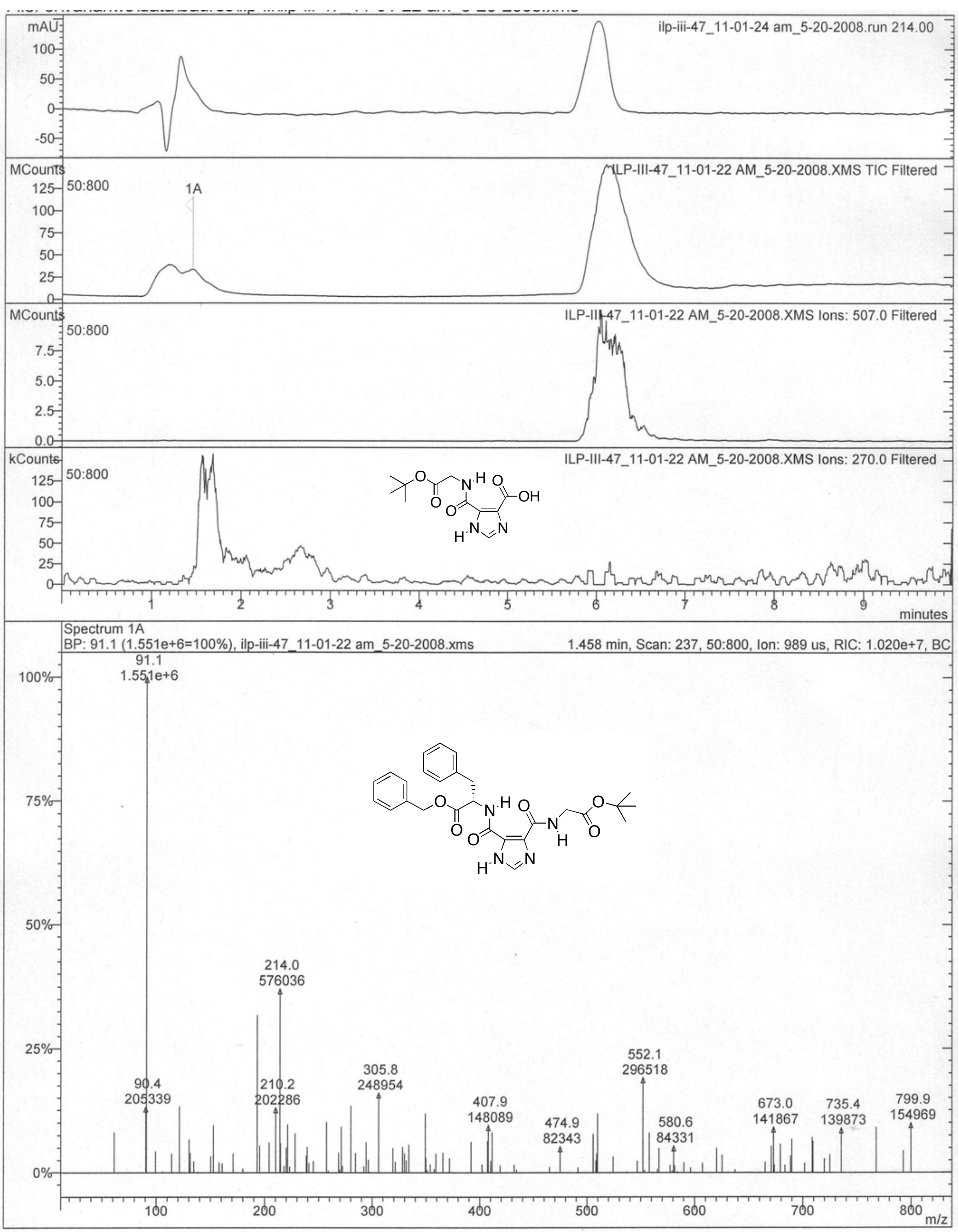

Figure S49. LC/MS data for the crude reaction to yield **4{**16**}**.

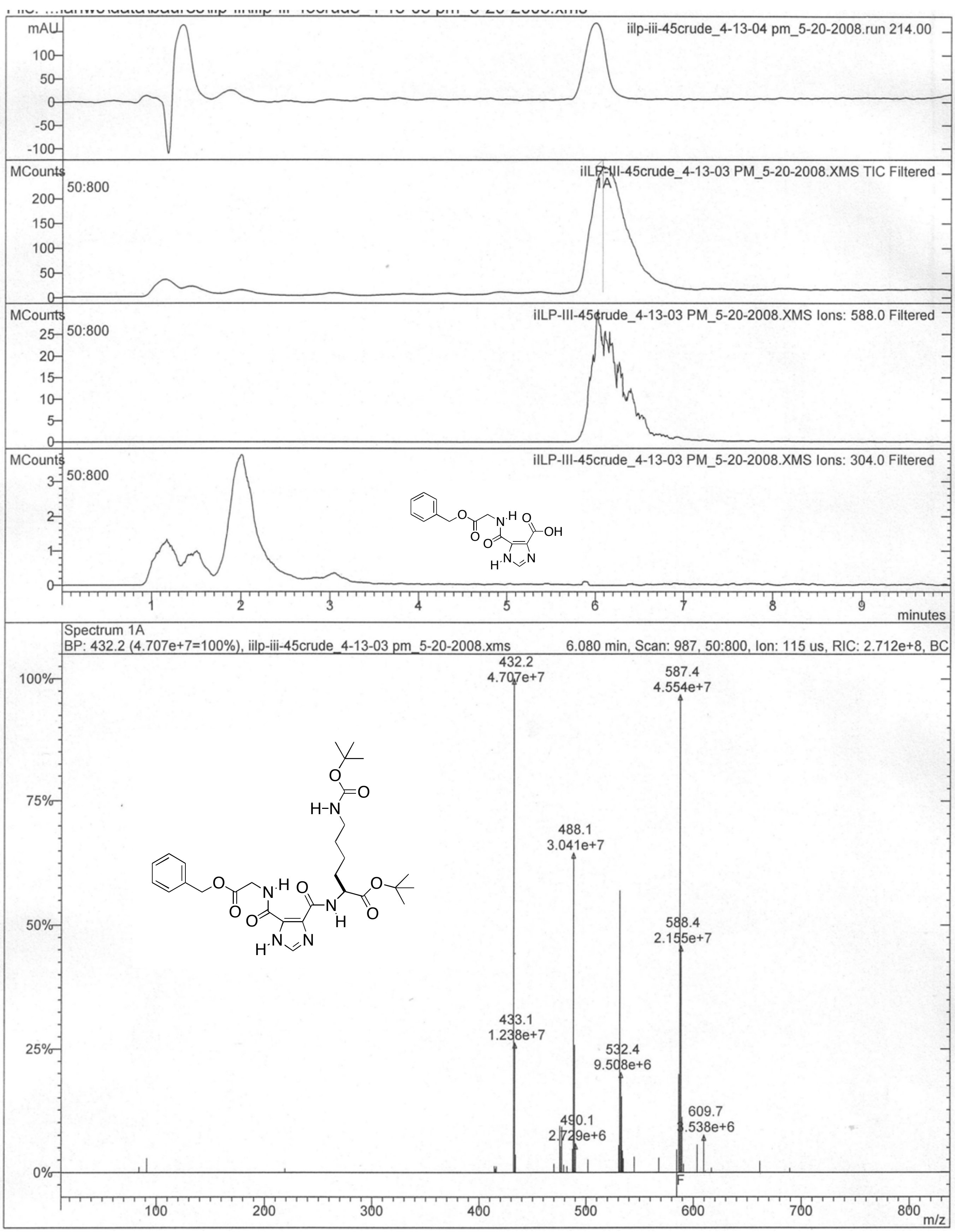

Figure S50. LC/MS data for the crude reaction to yield **4{**24**}**.

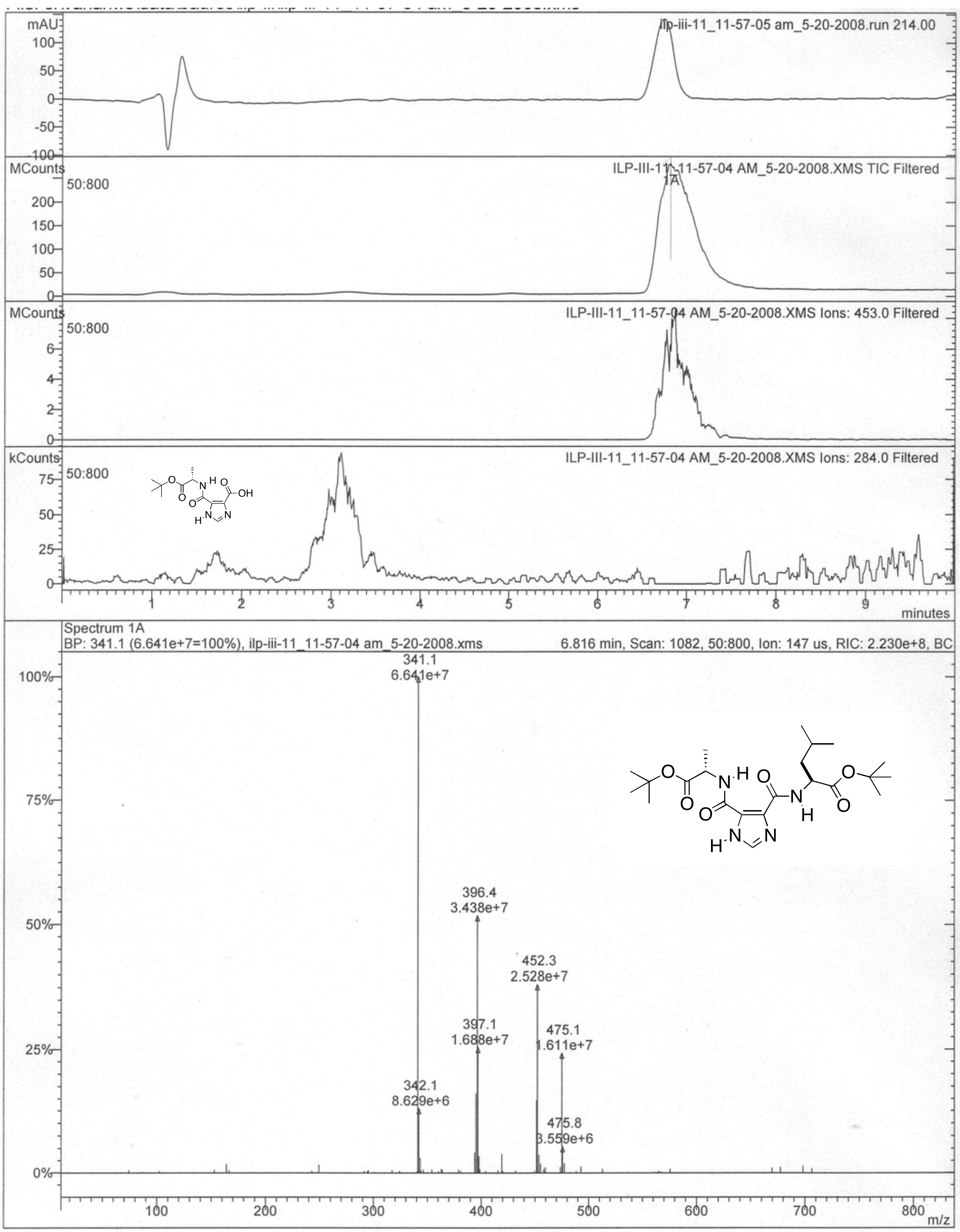

Figure S51. LC/MS data for the crude reaction to yield **4{**26**}**.

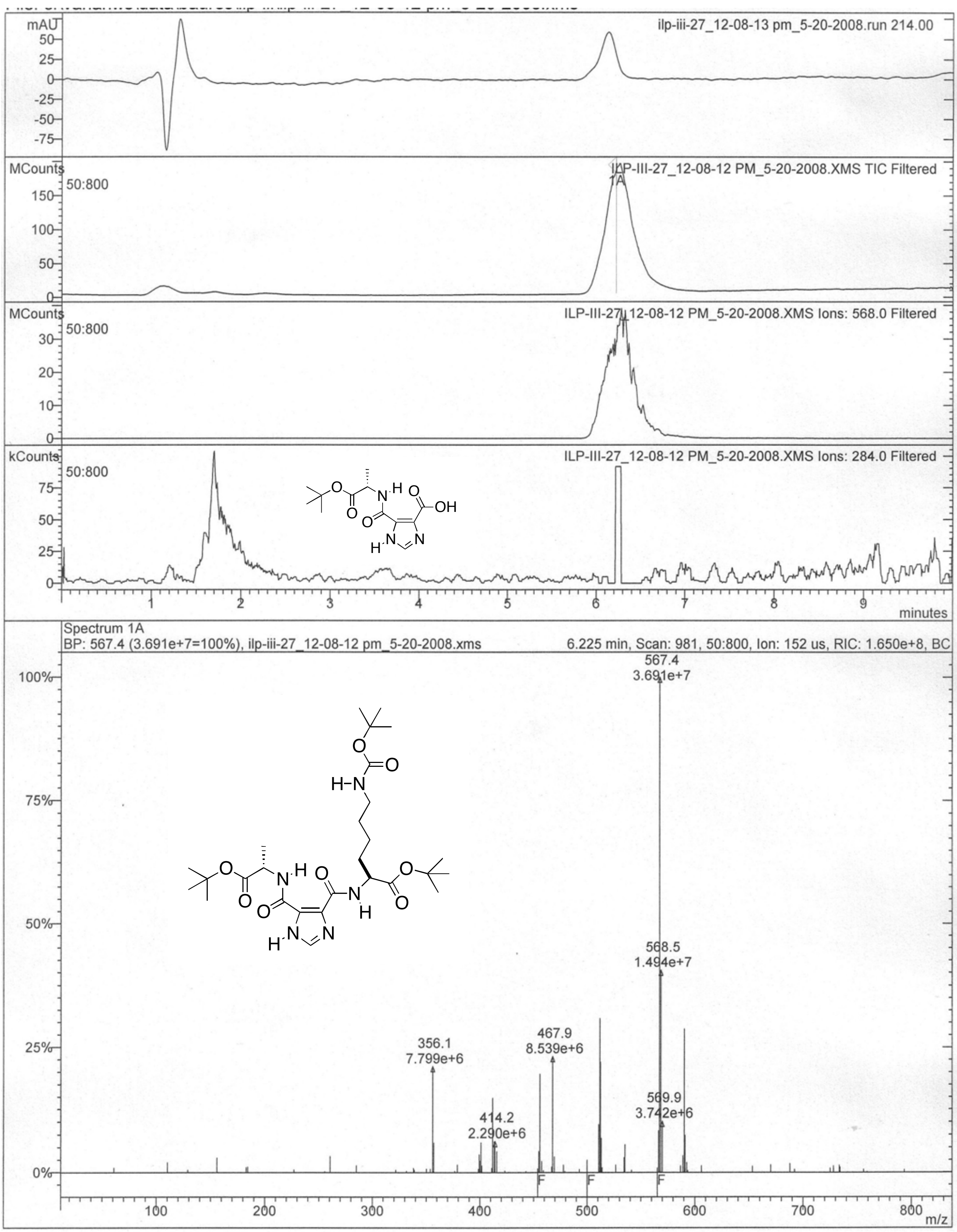

Figure S52. LC/MS data for the crude reaction to yield **4{**30}.

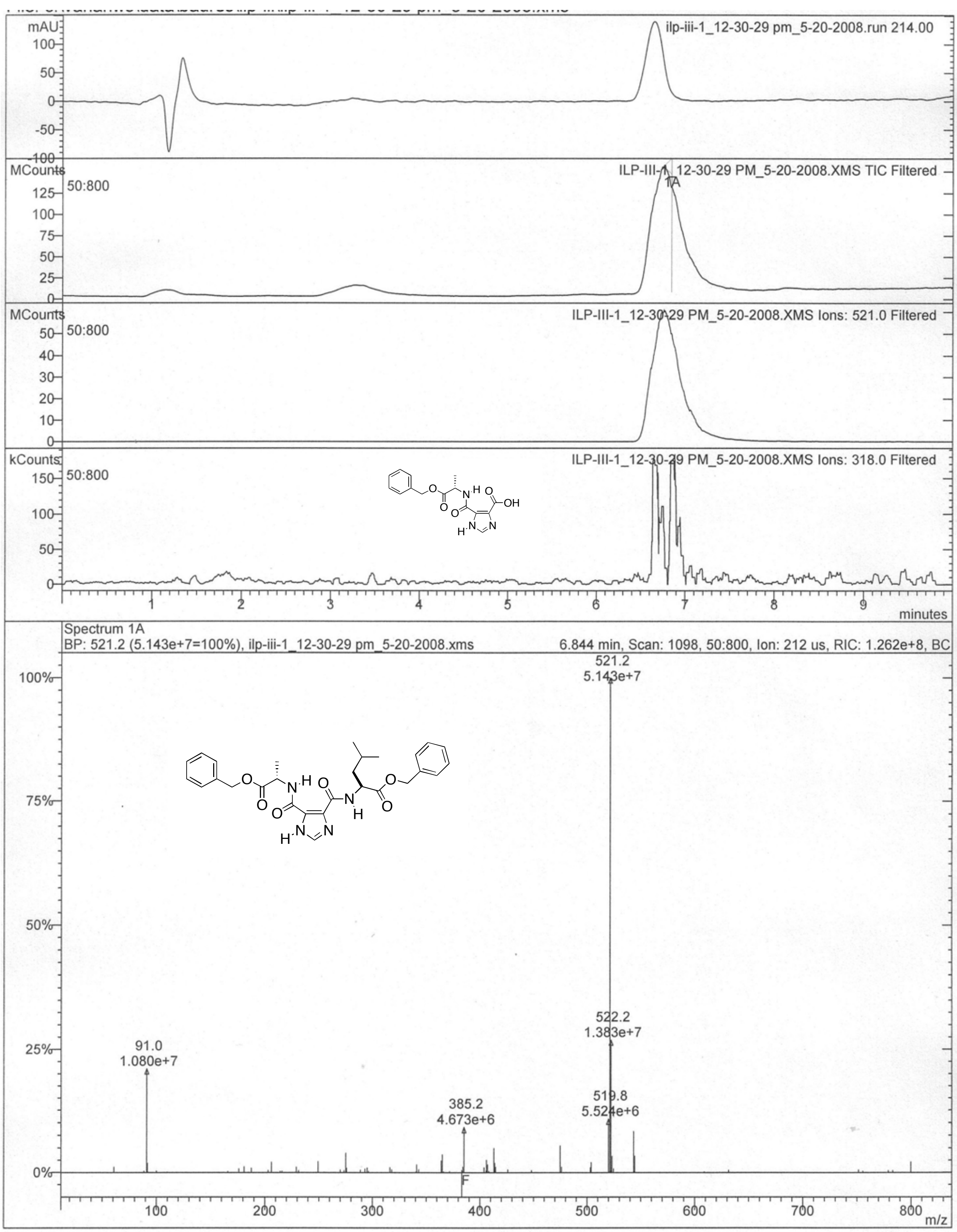

Figure S53. LC/MS data for the crude reaction to yield **4{**32**}**.

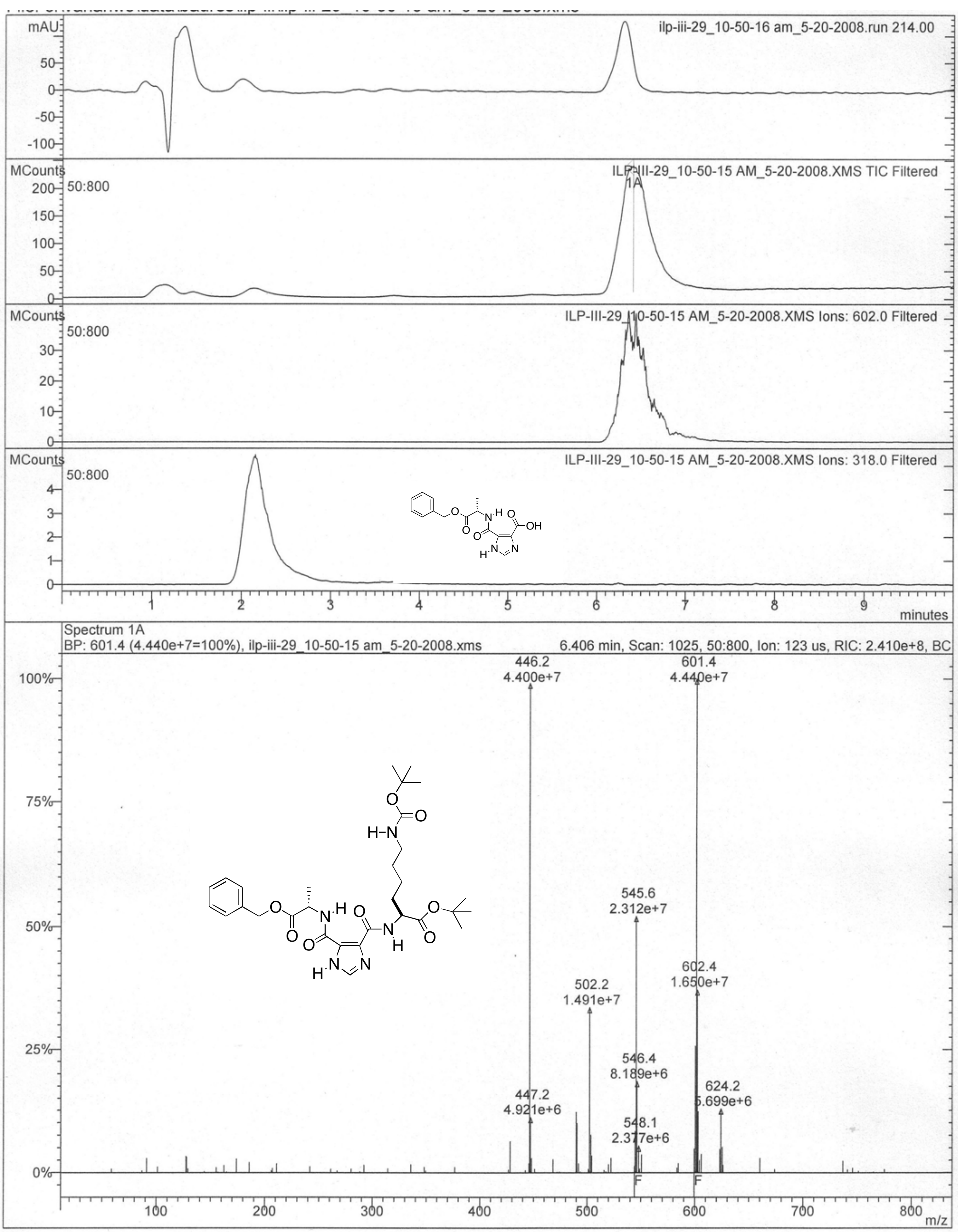

Figure S54. LC/MS data for the crude reaction to yield **4{**35**}**.

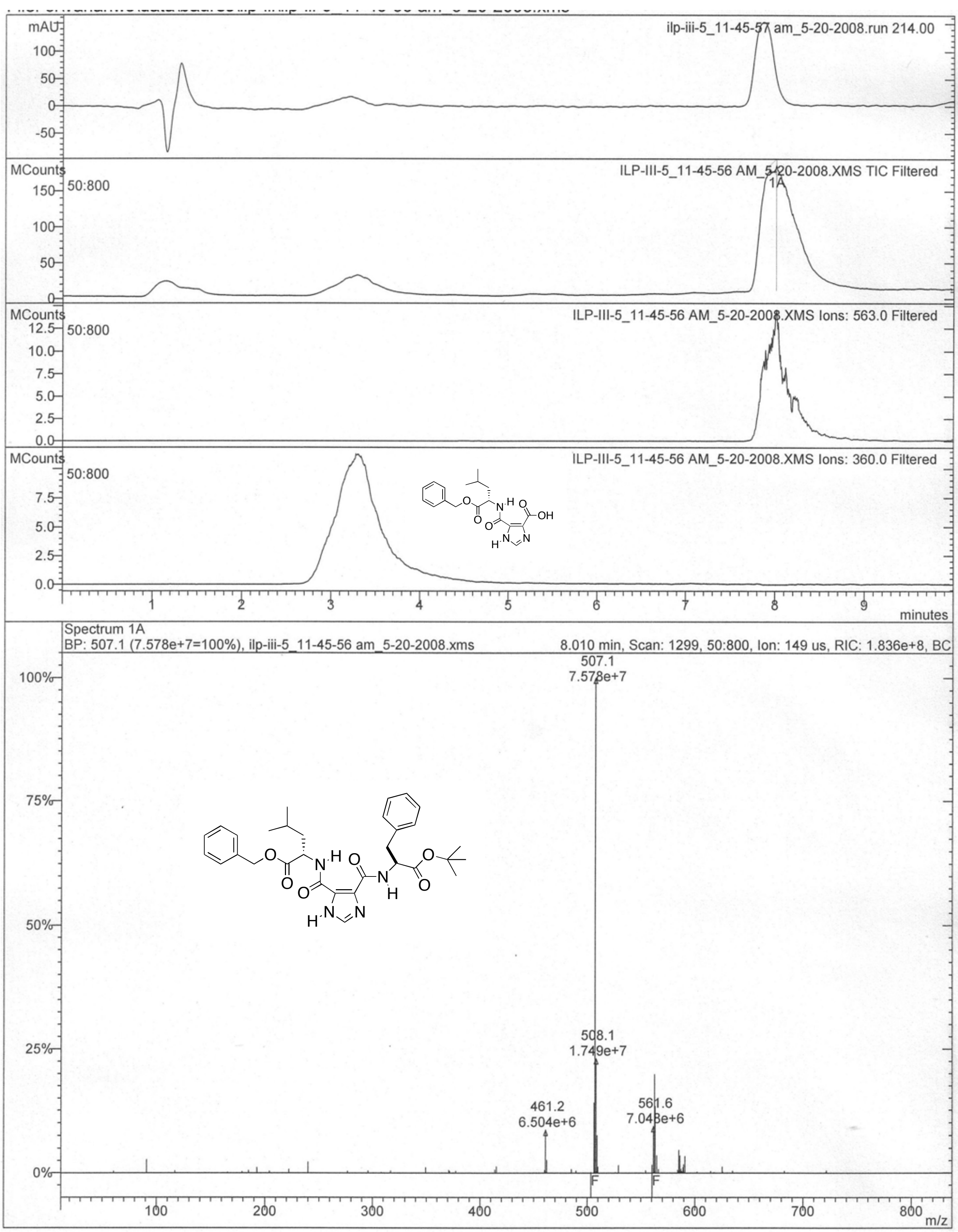

Figure S55. LC/MS data for the crude reaction to yield **4**{40}.

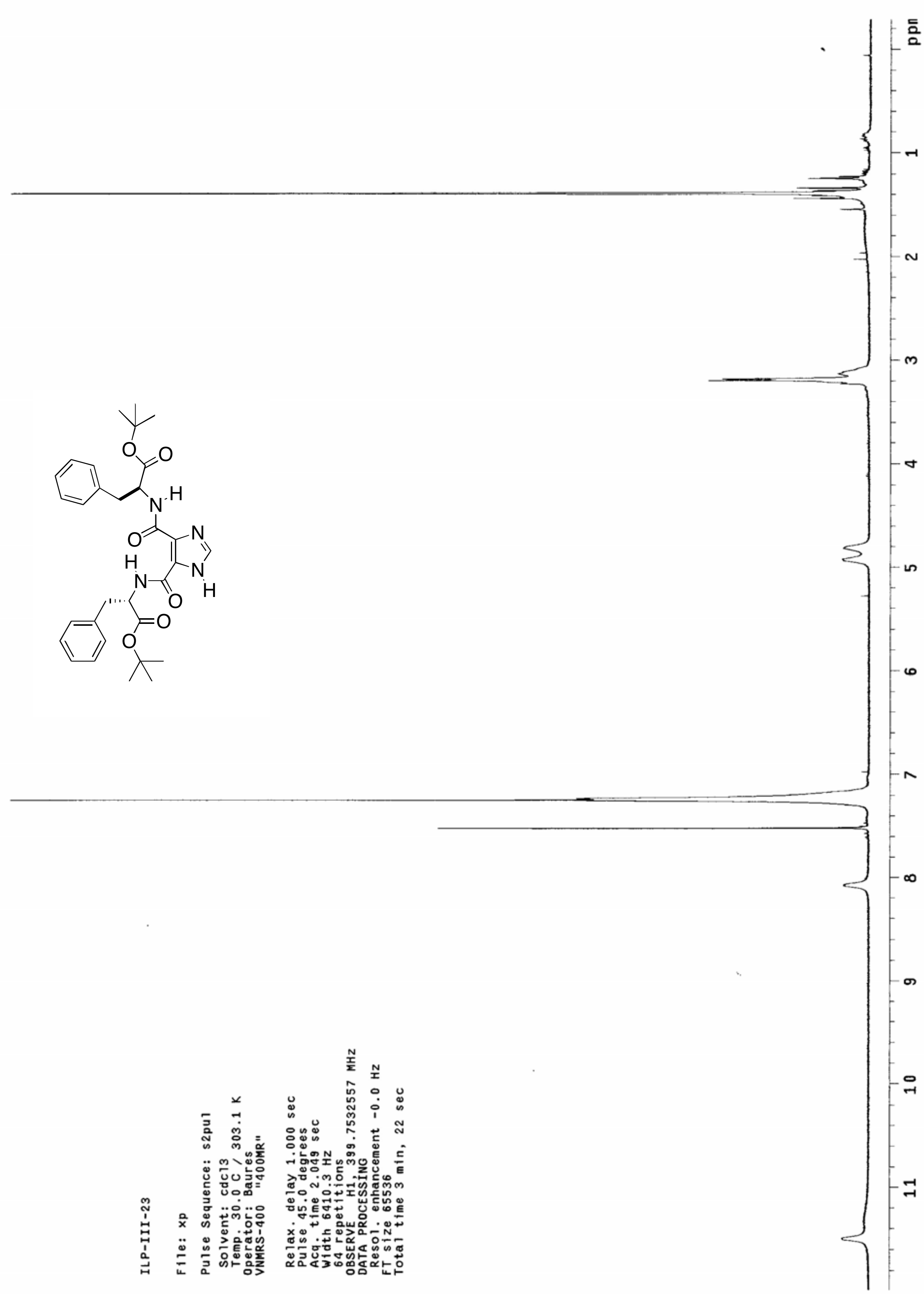

Figure S56. ^{1}H-NMR for **4{**7}.

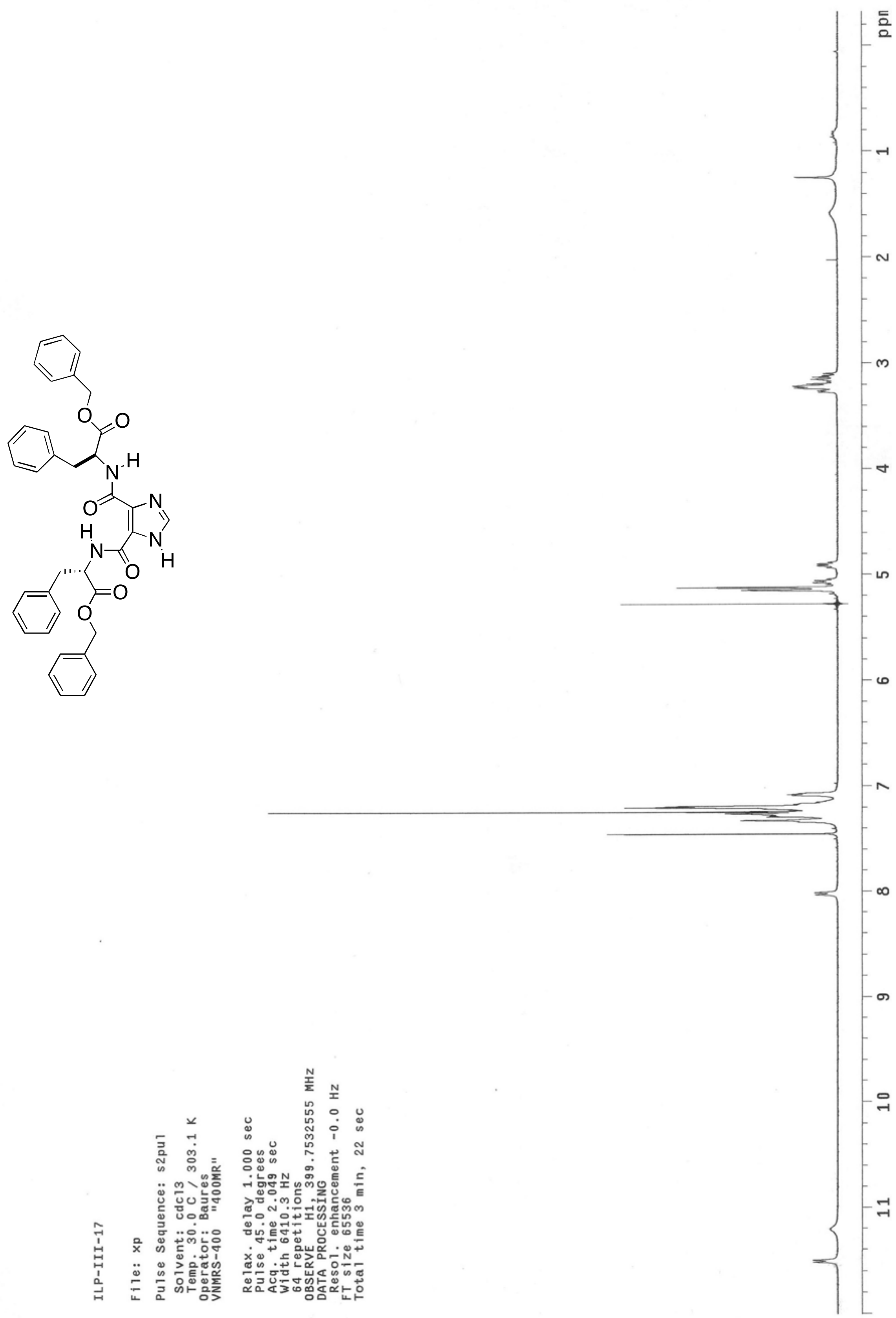

Figure S57. ^{1}H-NMR for **4{**8}.

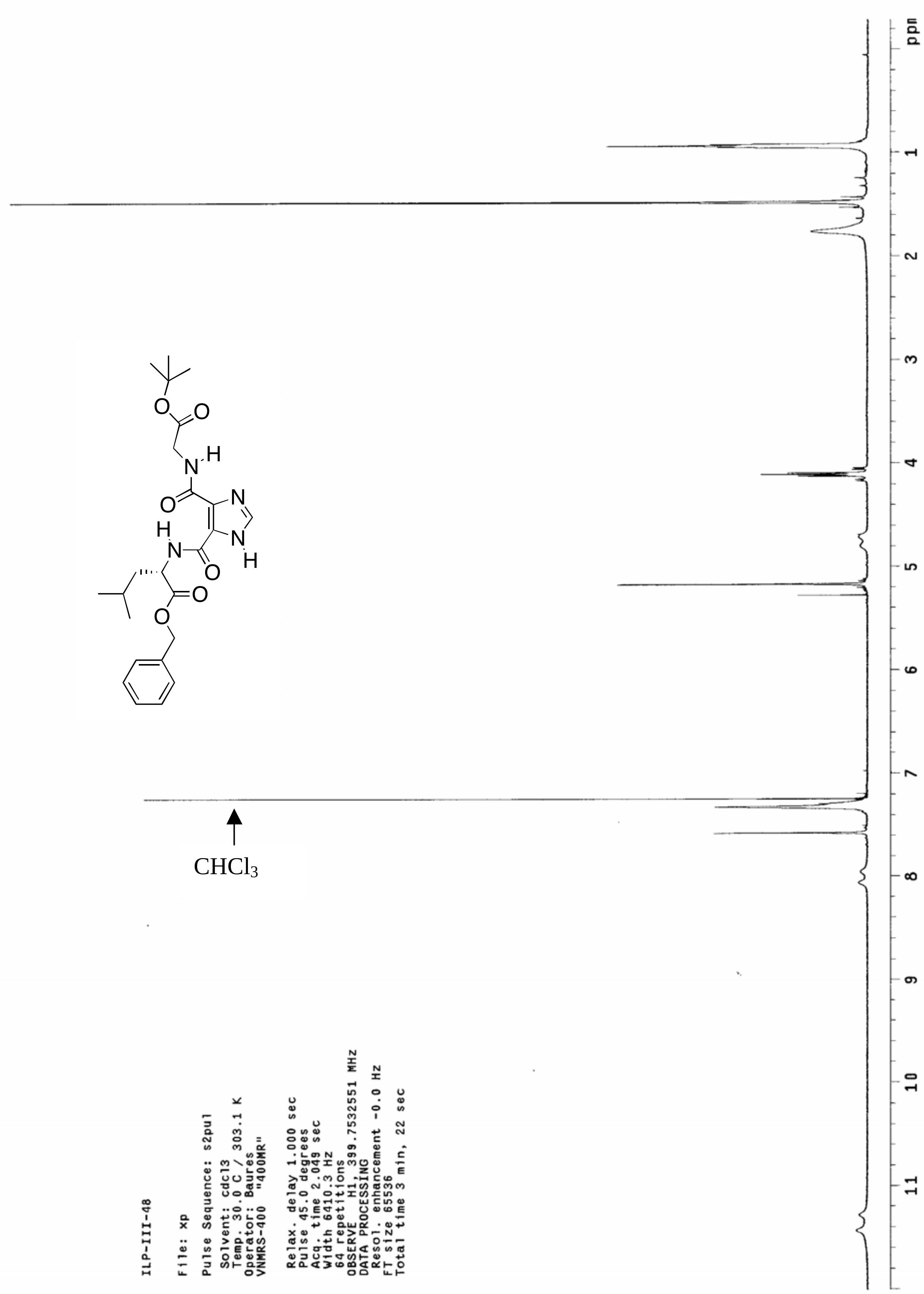

Figure S58. ^{1}H-NMR for **4{**14}.

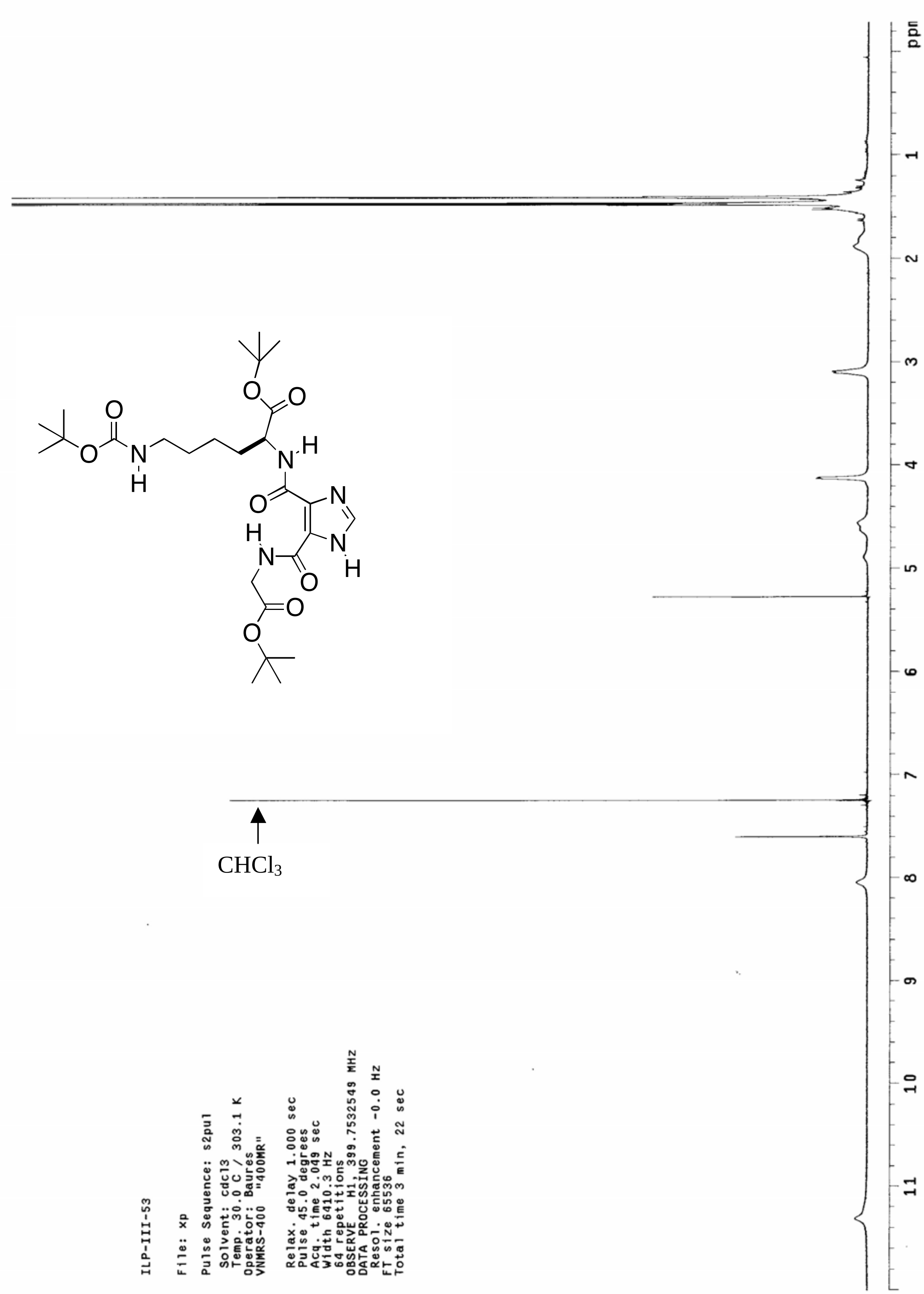

Figure 59. ^{1}H-NMR for **4{**17}.

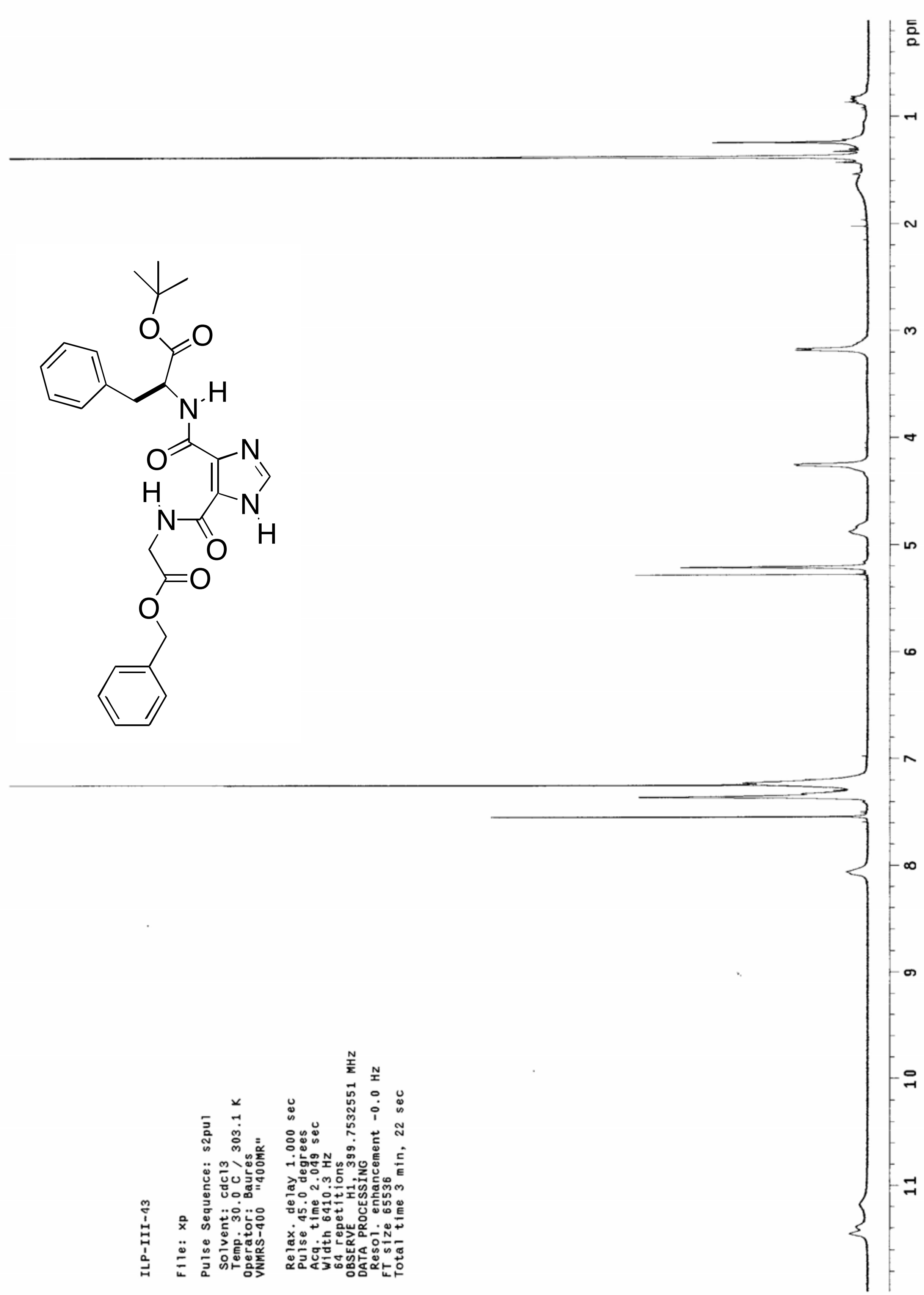

Figure S60. ^{1}H-NMR for **4{**22}.

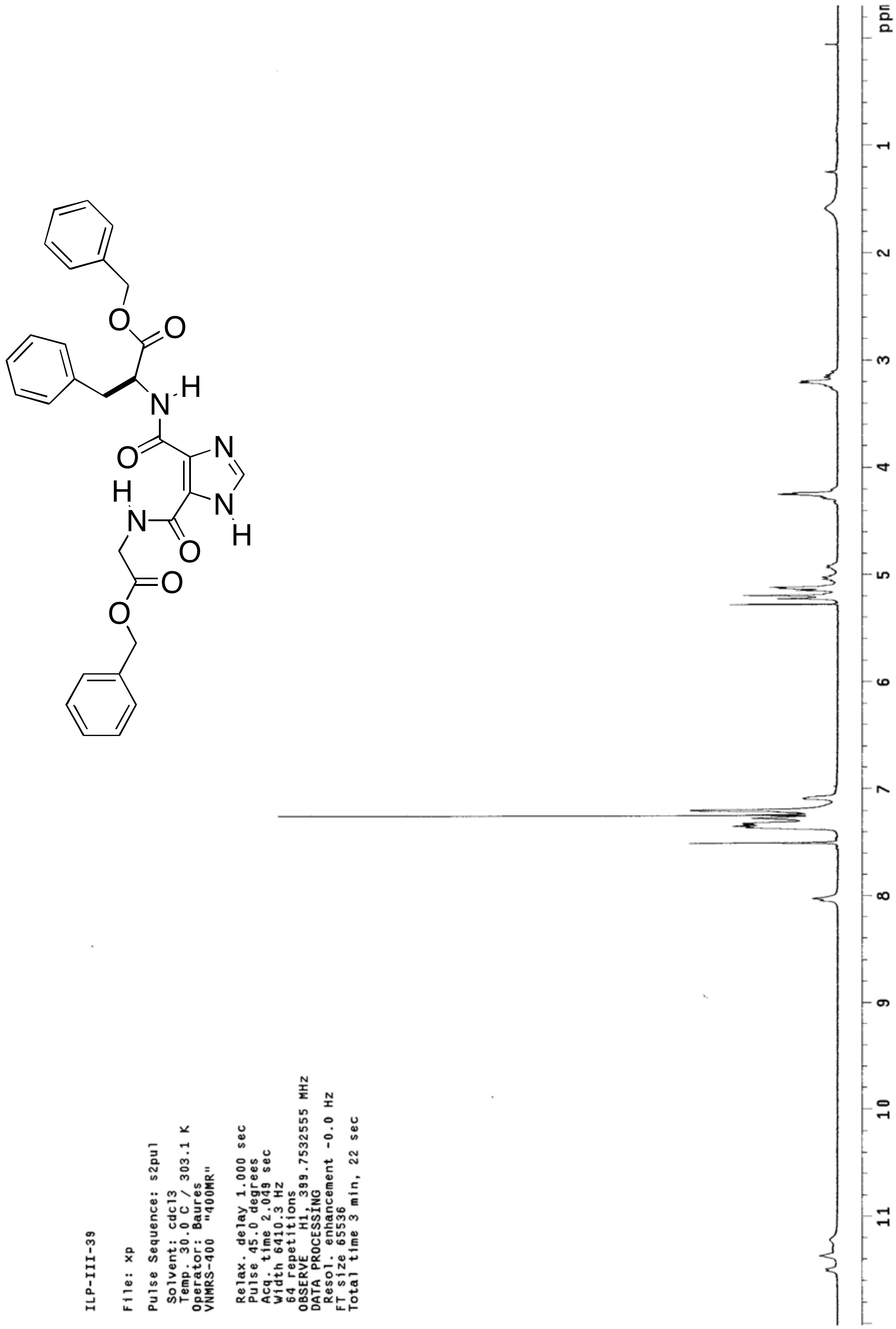

Figure S61. ^{1}H-NMR for **4{**23}.

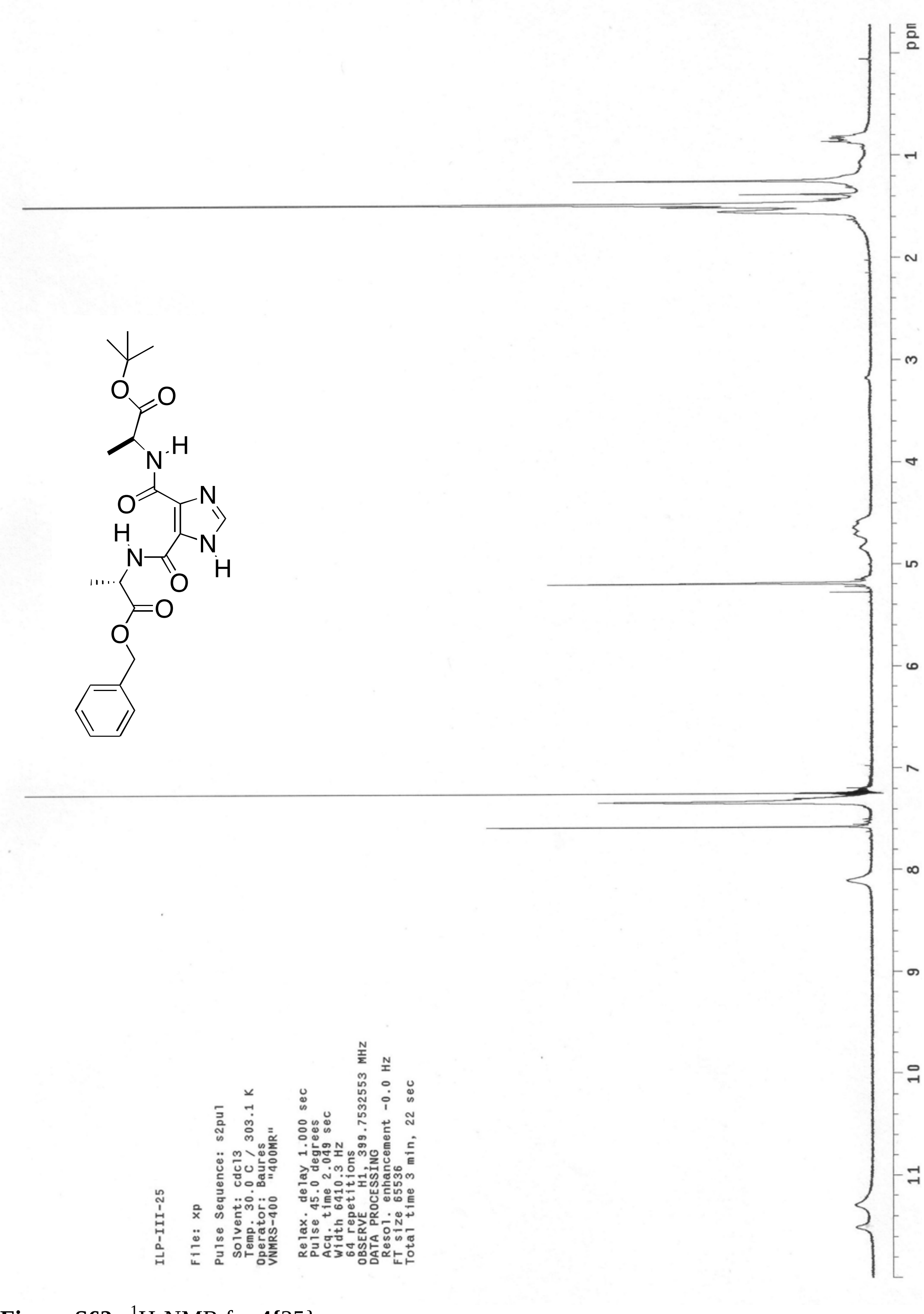

Figure S62. ^{1}H-NMR for **4{**25}.

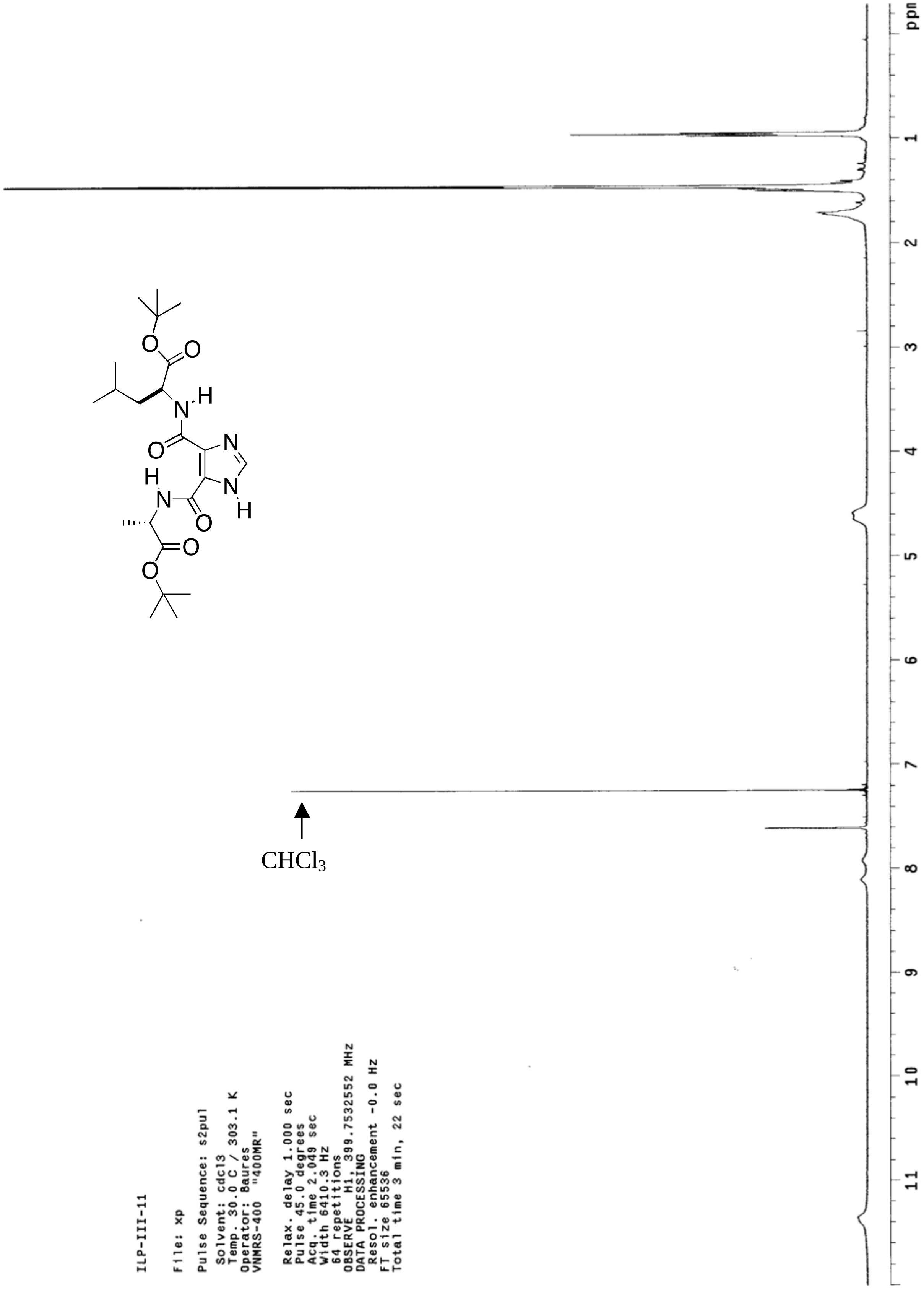

Figure S63. ^{1}H-NMR for **4{**26}.

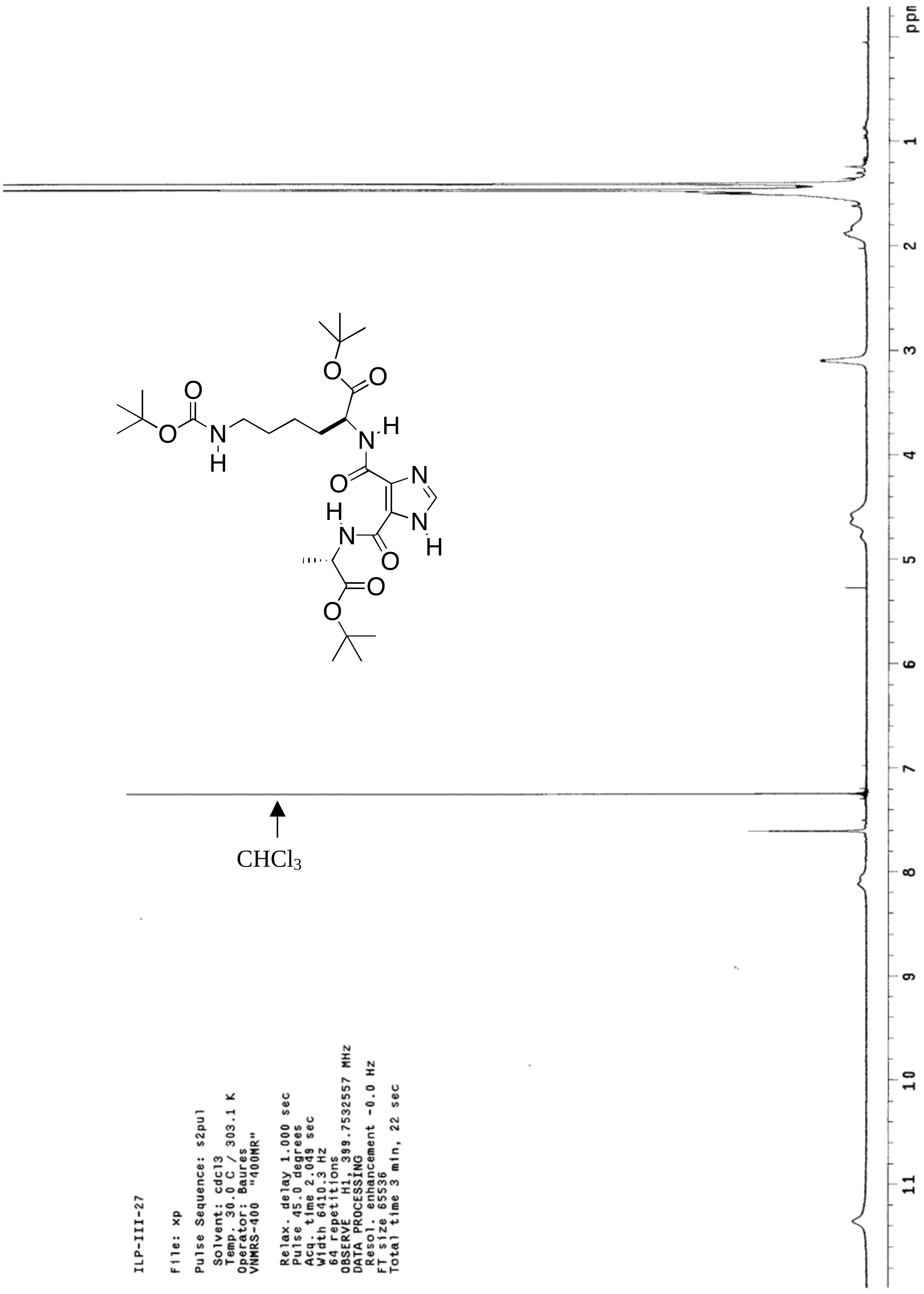

Figure S64. ^{1}H-NMR for **4{**30}.

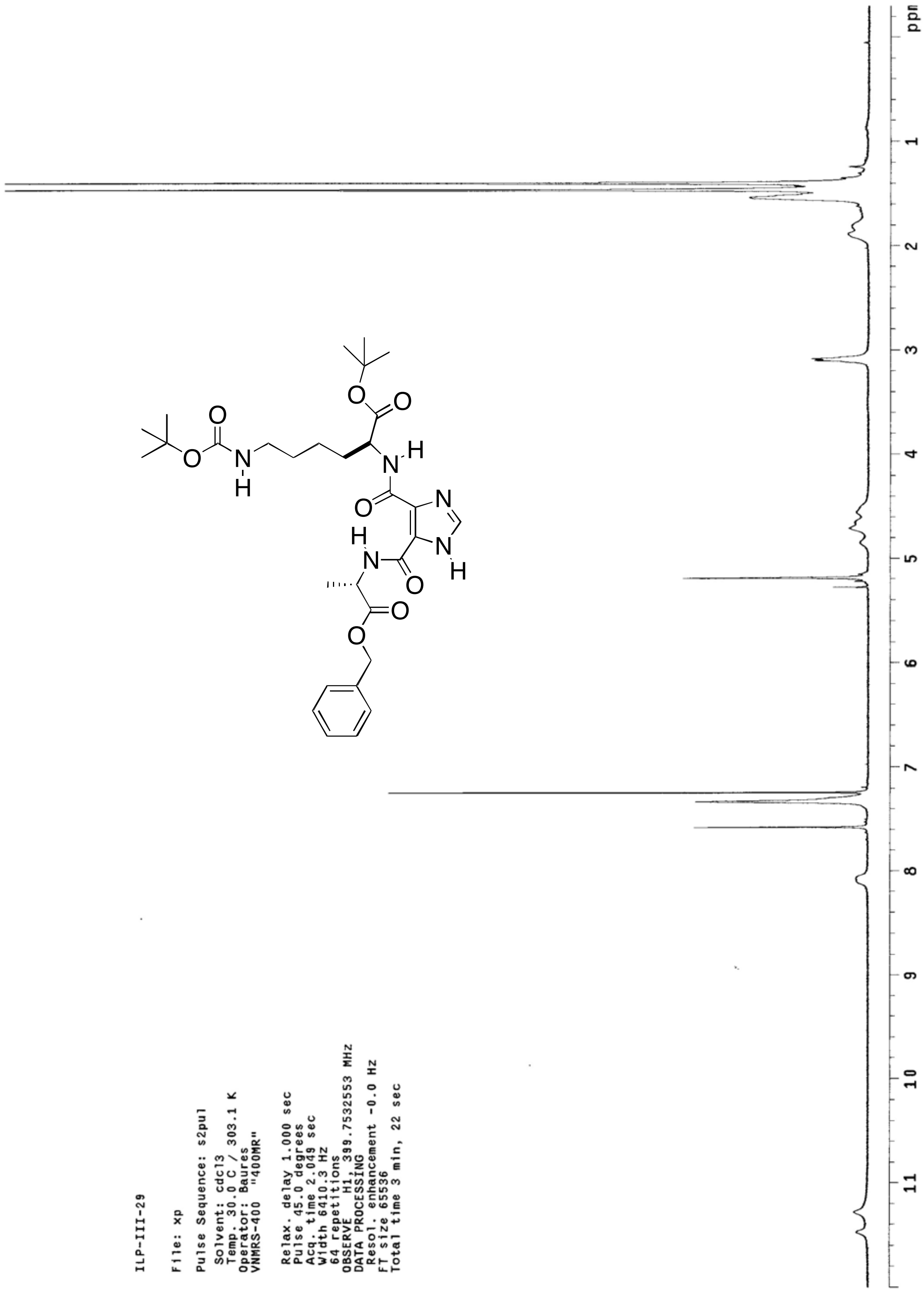

Figure S65. ^{1}H-NMR for **4{**35}.

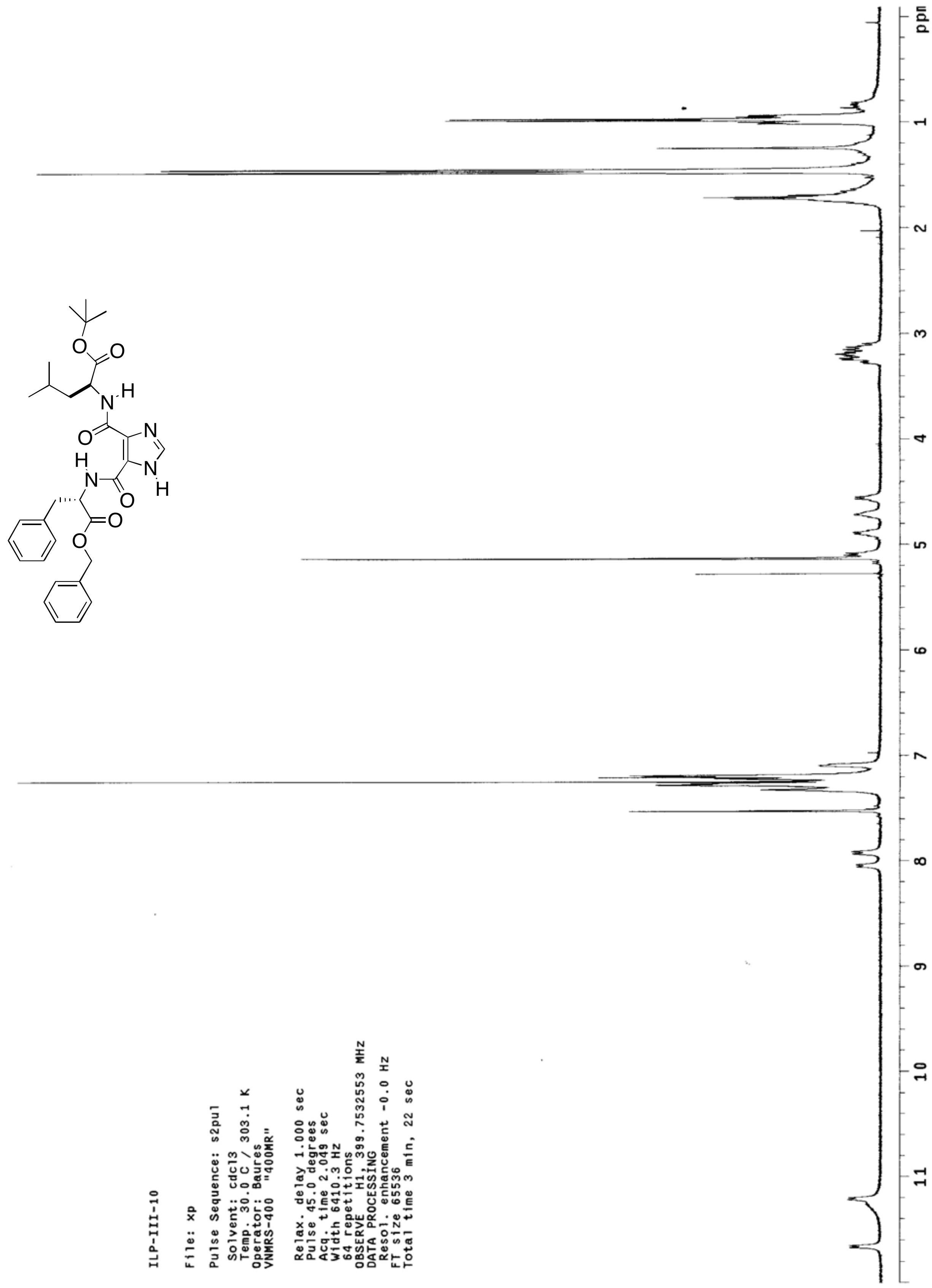

Figure S66. ^{1}H-NMR for **4{**38}.

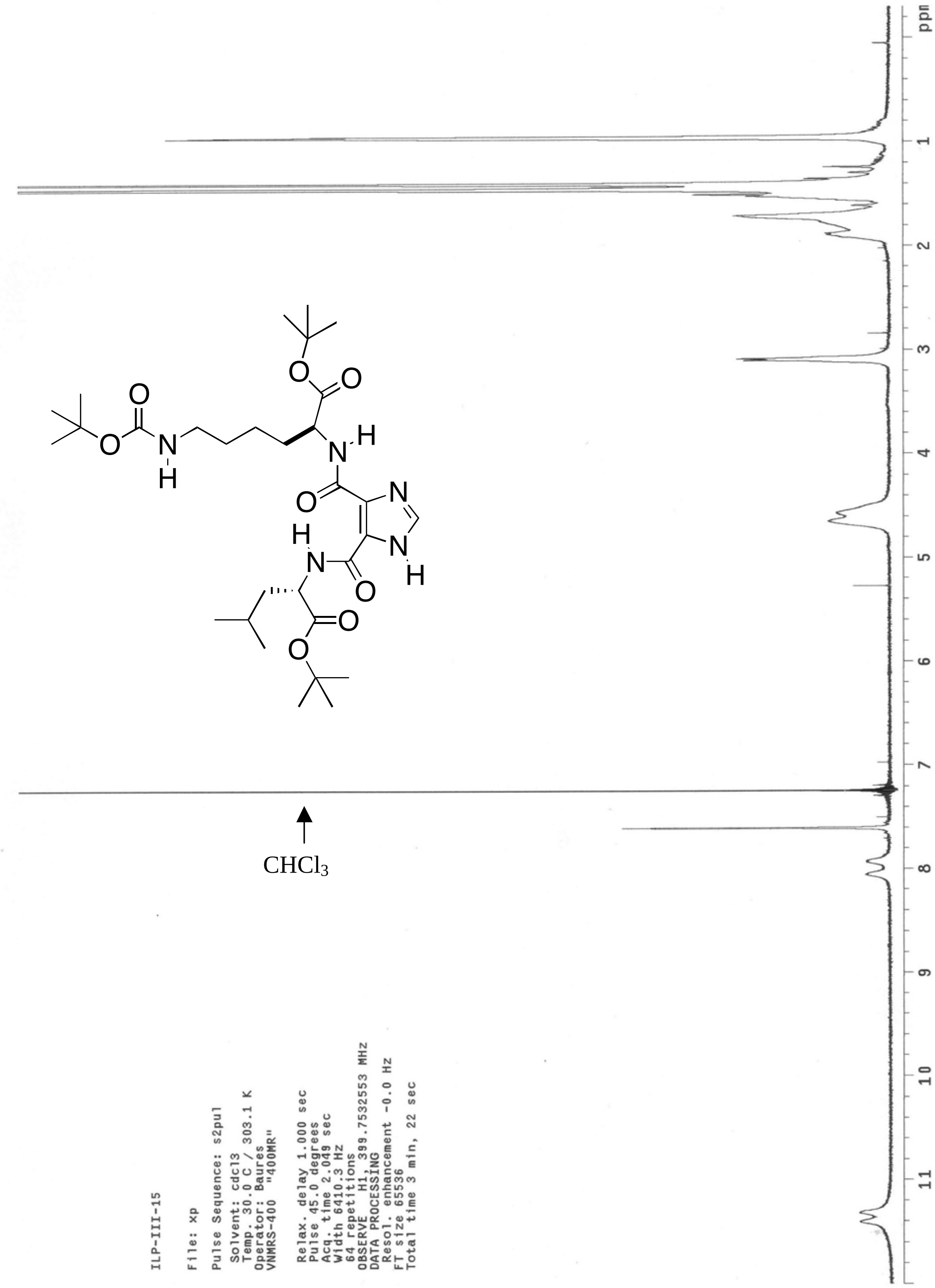

Figure S67. ^{1}H-NMR for **4{**39}.

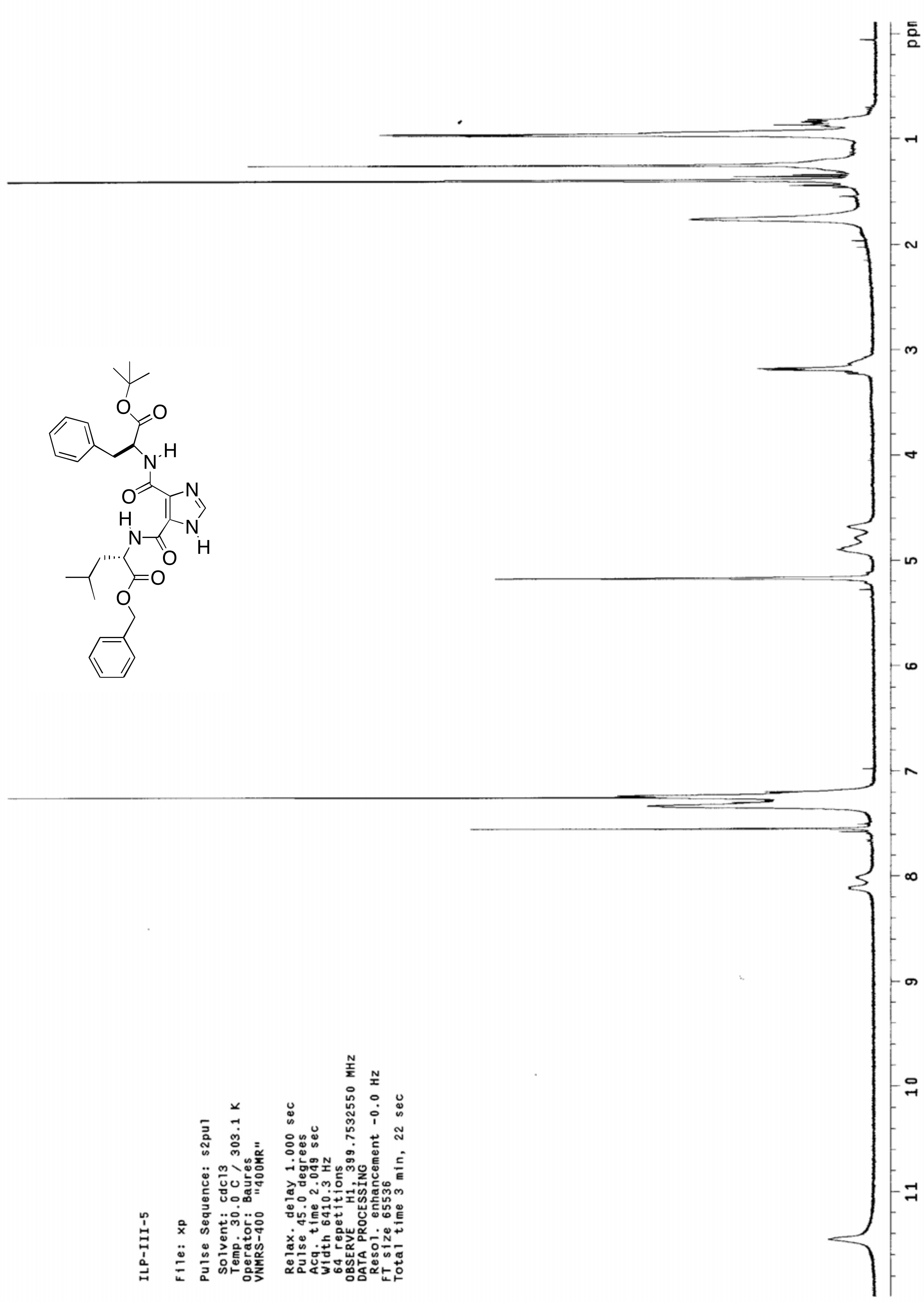

Figure S68. ^{1}H-NMR for **4{**40}.

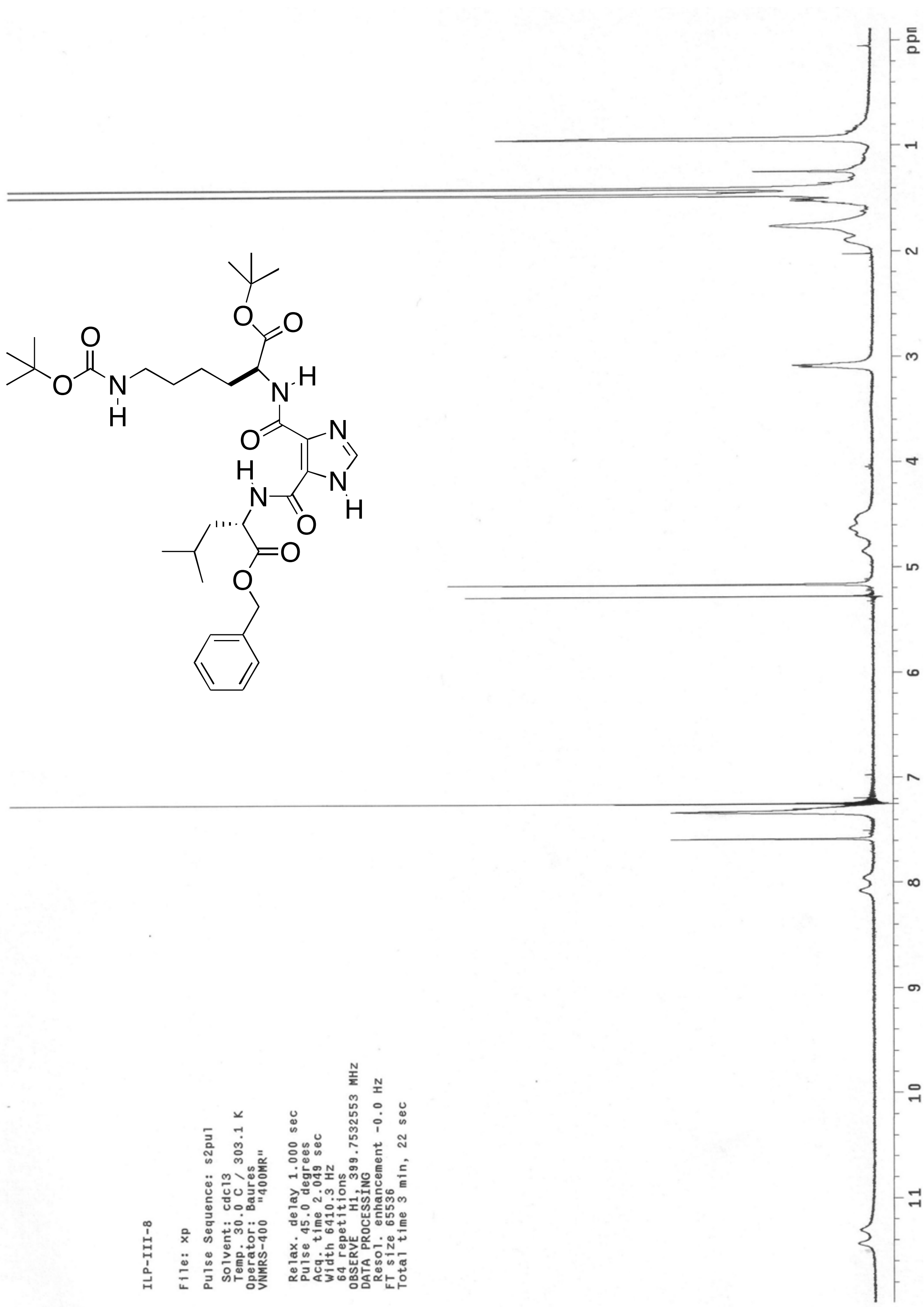

Figure S69. ^{1}H-NMR for **4{**42}.

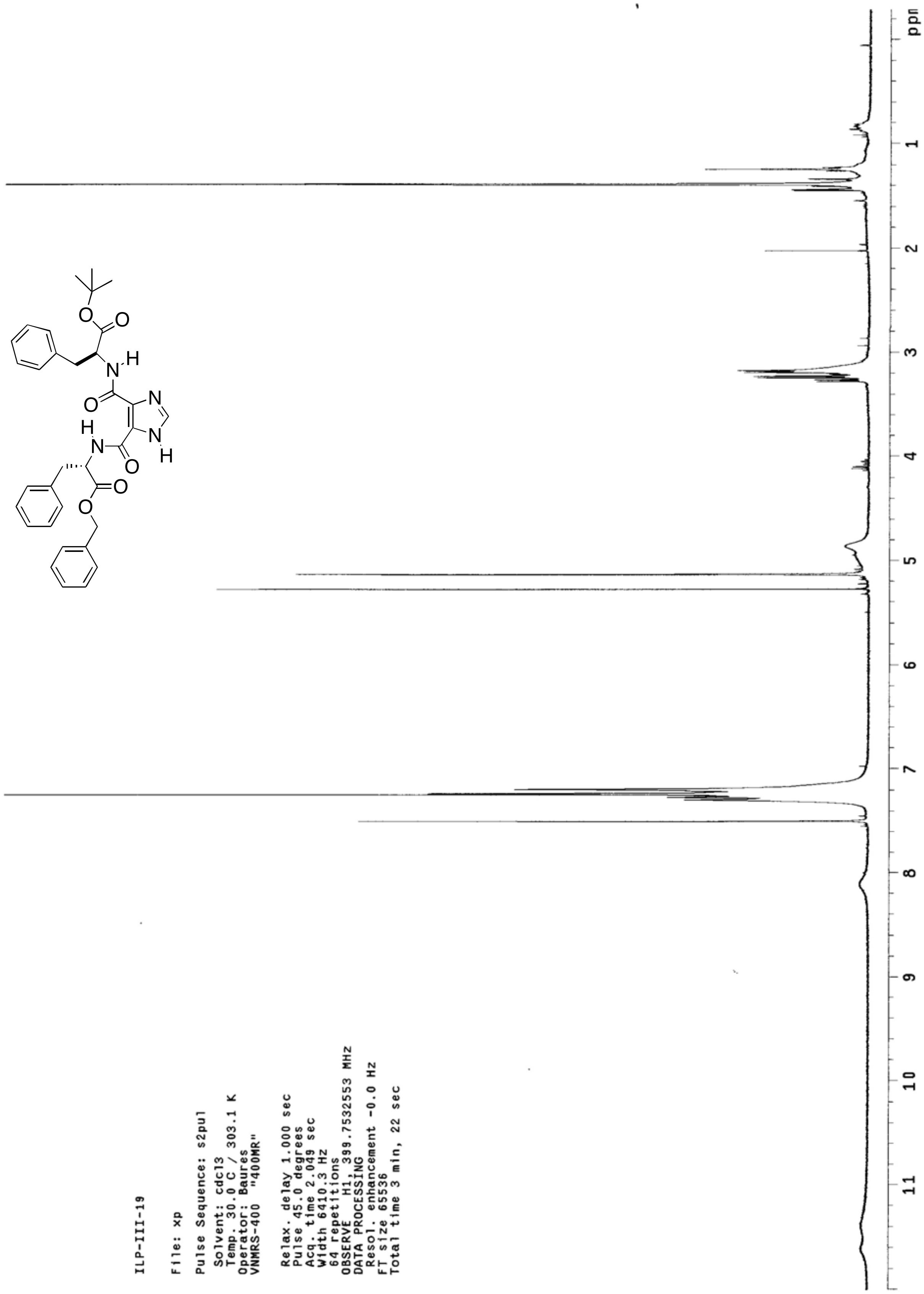

Figure S70. ^{1}H-NMR for **4{**43}.

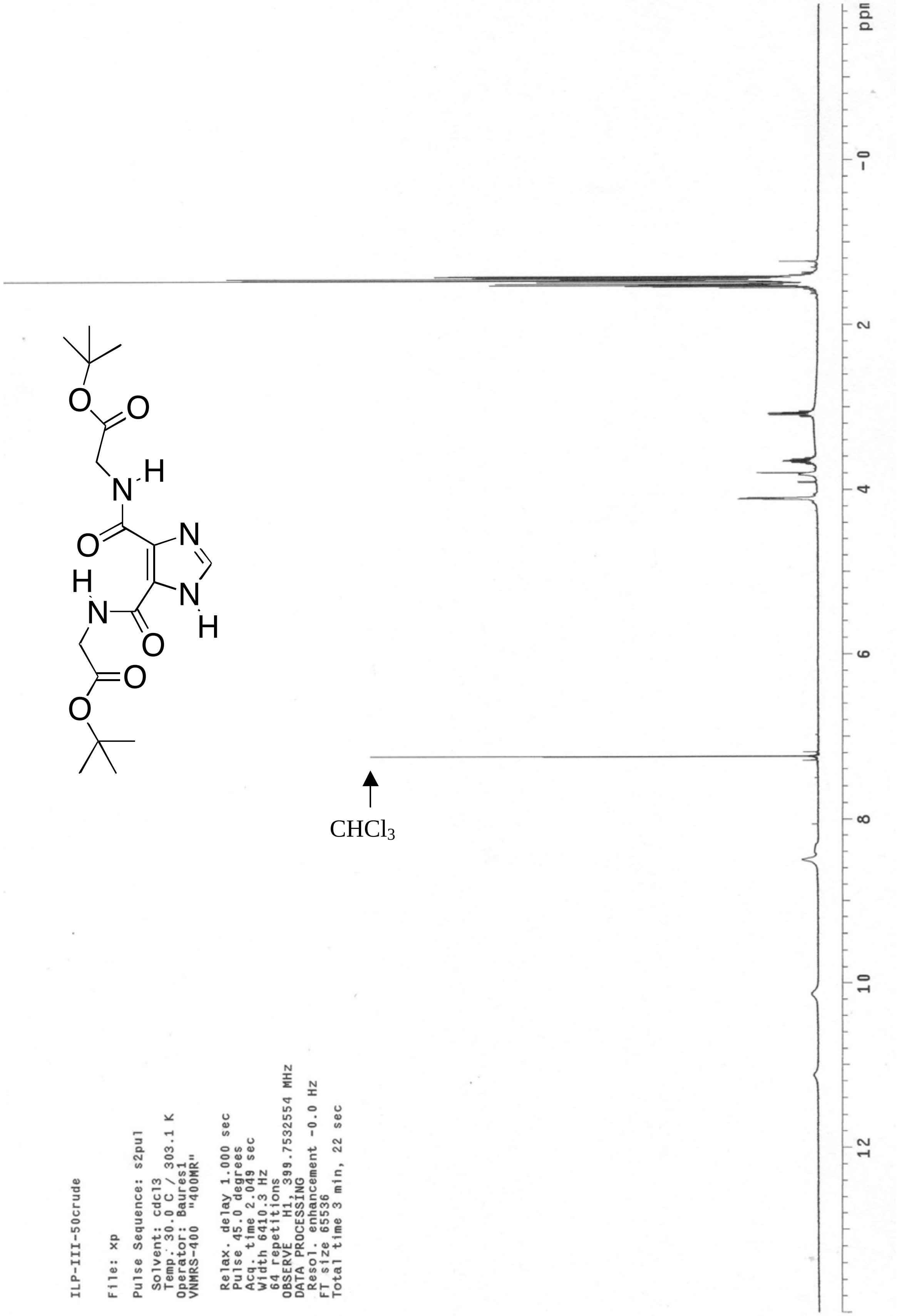

Figure S71. ^{1}H-NMR for the crude reaction to yield **4{**1}.

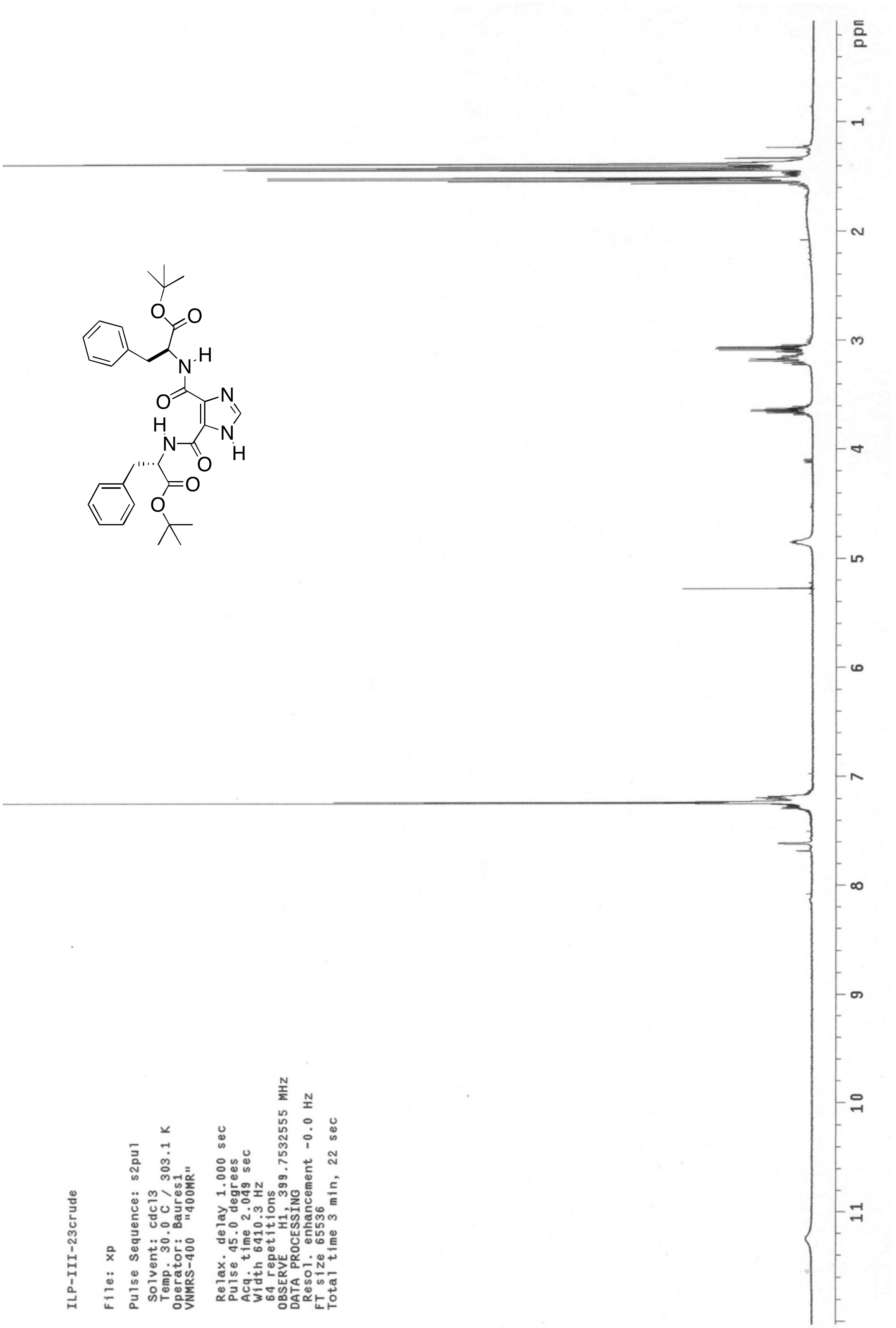

Figure S72. ^{1}H-NMR for the crude reaction to yield **4{**7}.

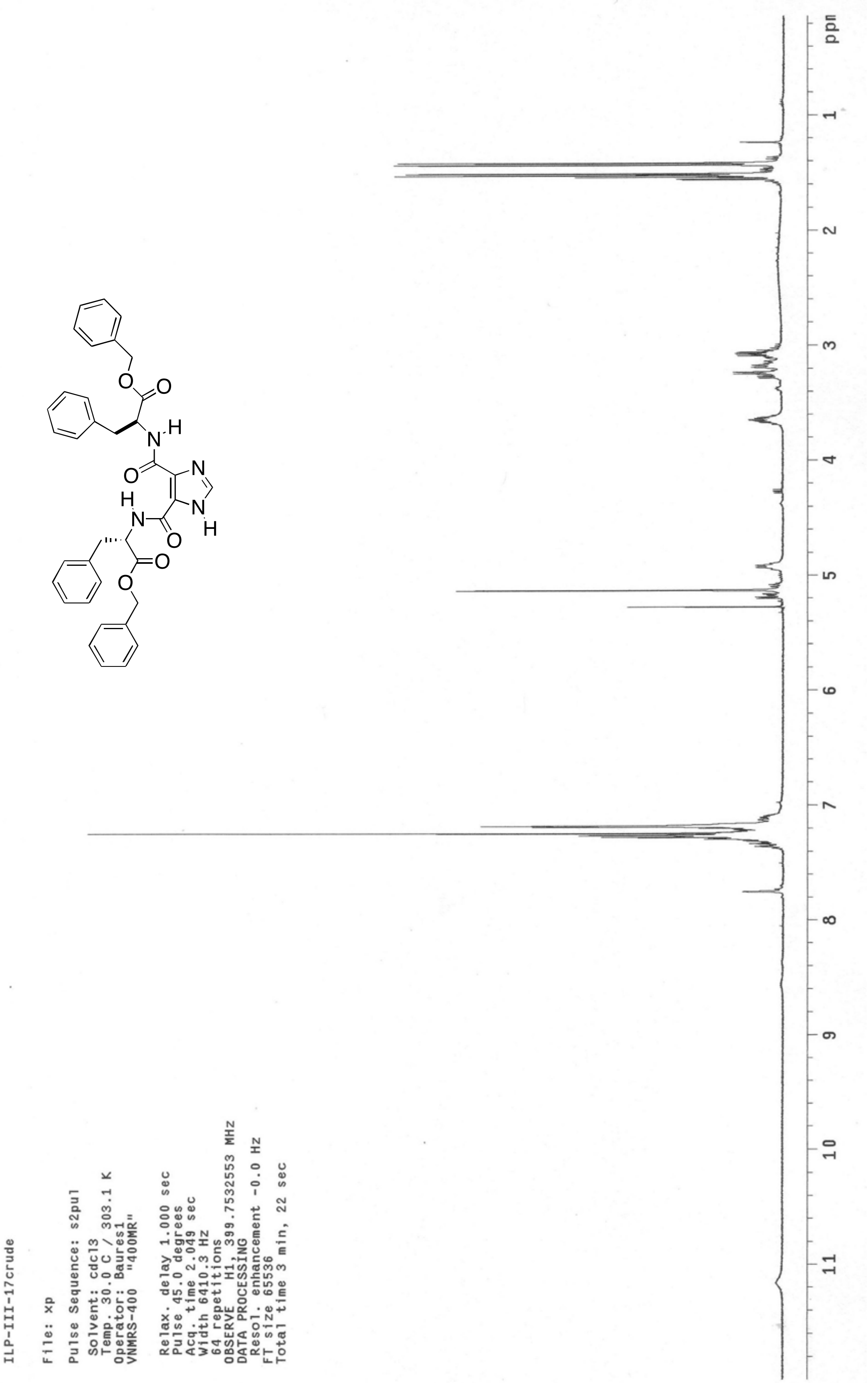

Figure S73. ^{1}H-NMR for the crude reaction to yield **4{**8}.

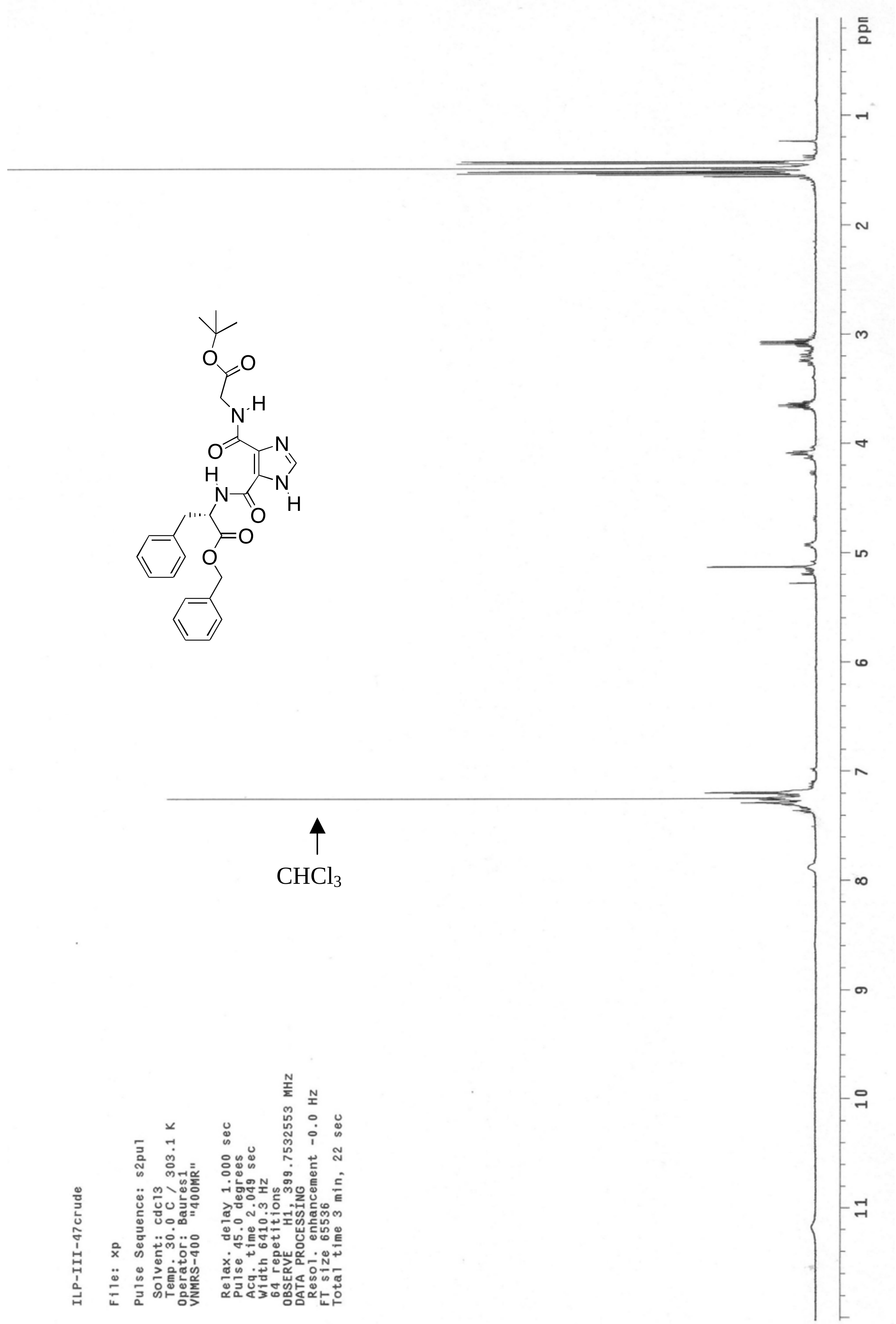

Figure S74. ^{1}H-NMR for the crude reaction to yield **4{**16}.

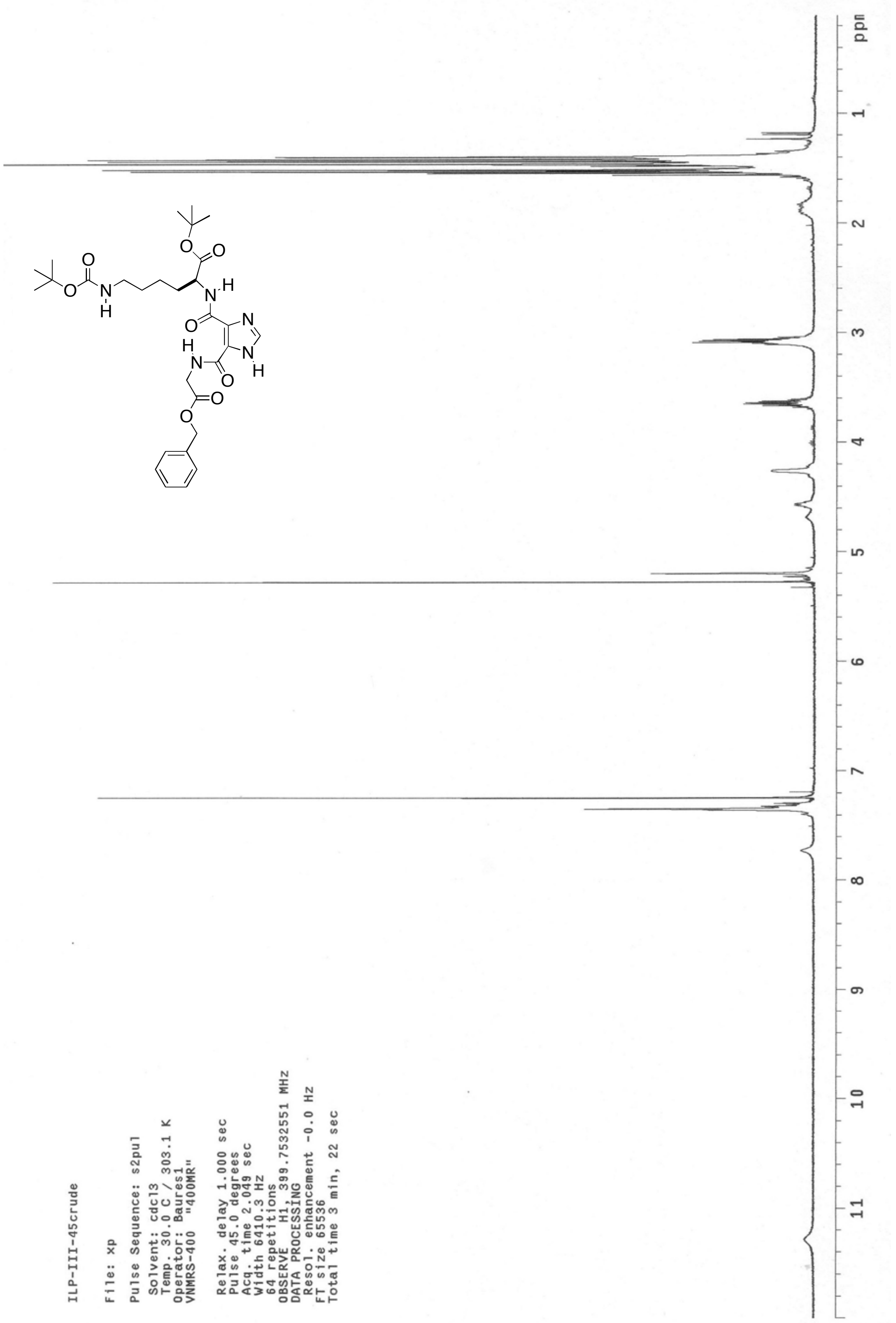

Figure S75. ^{1}H-NMR for the crude reaction to yield **4{**24}.

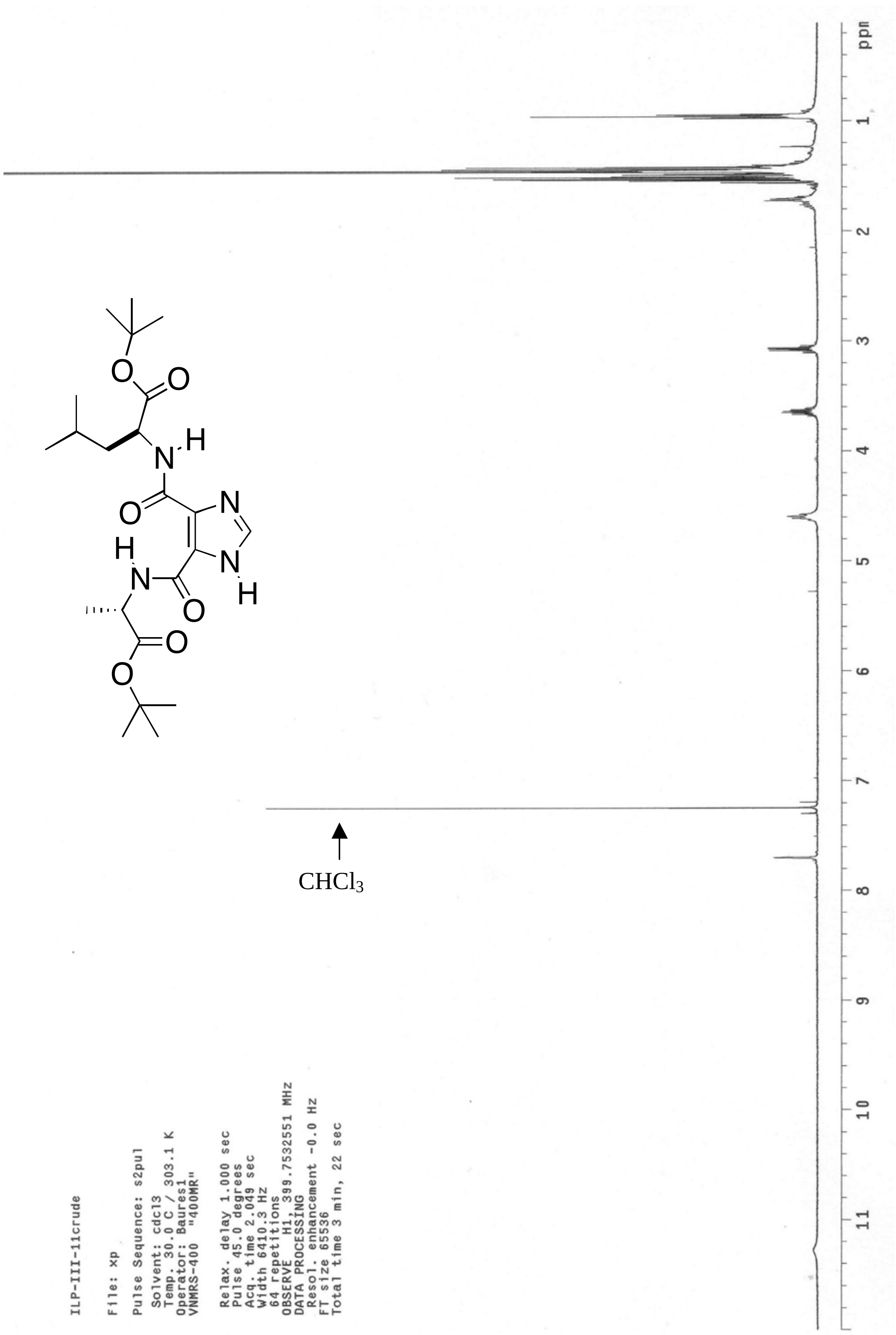

Figure S76. ^{1}H-NMR for the crude reaction to yield **4{**26}.

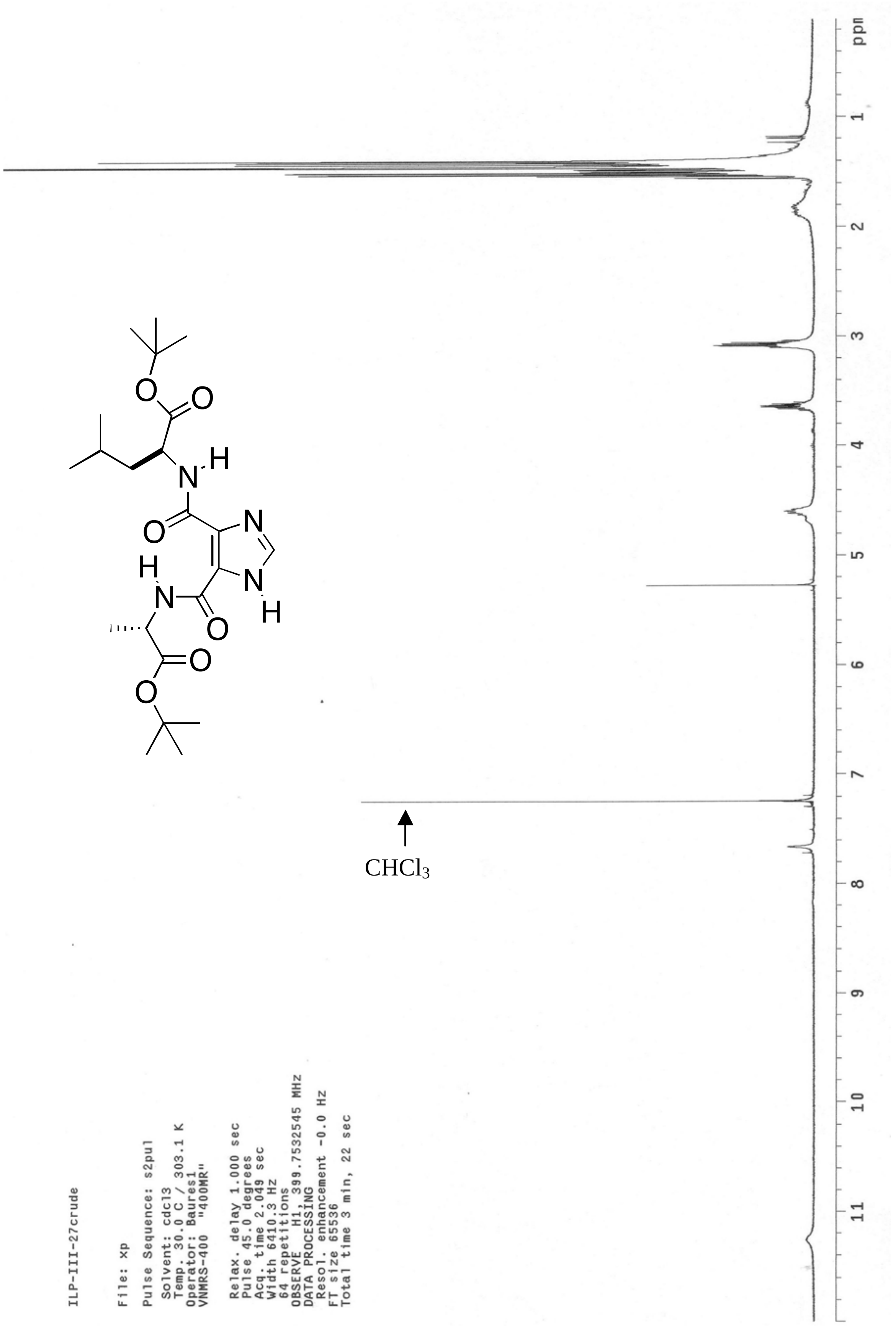

Figure S77. ^{1}H-NMR for the crude reaction to yield **4{**30}.

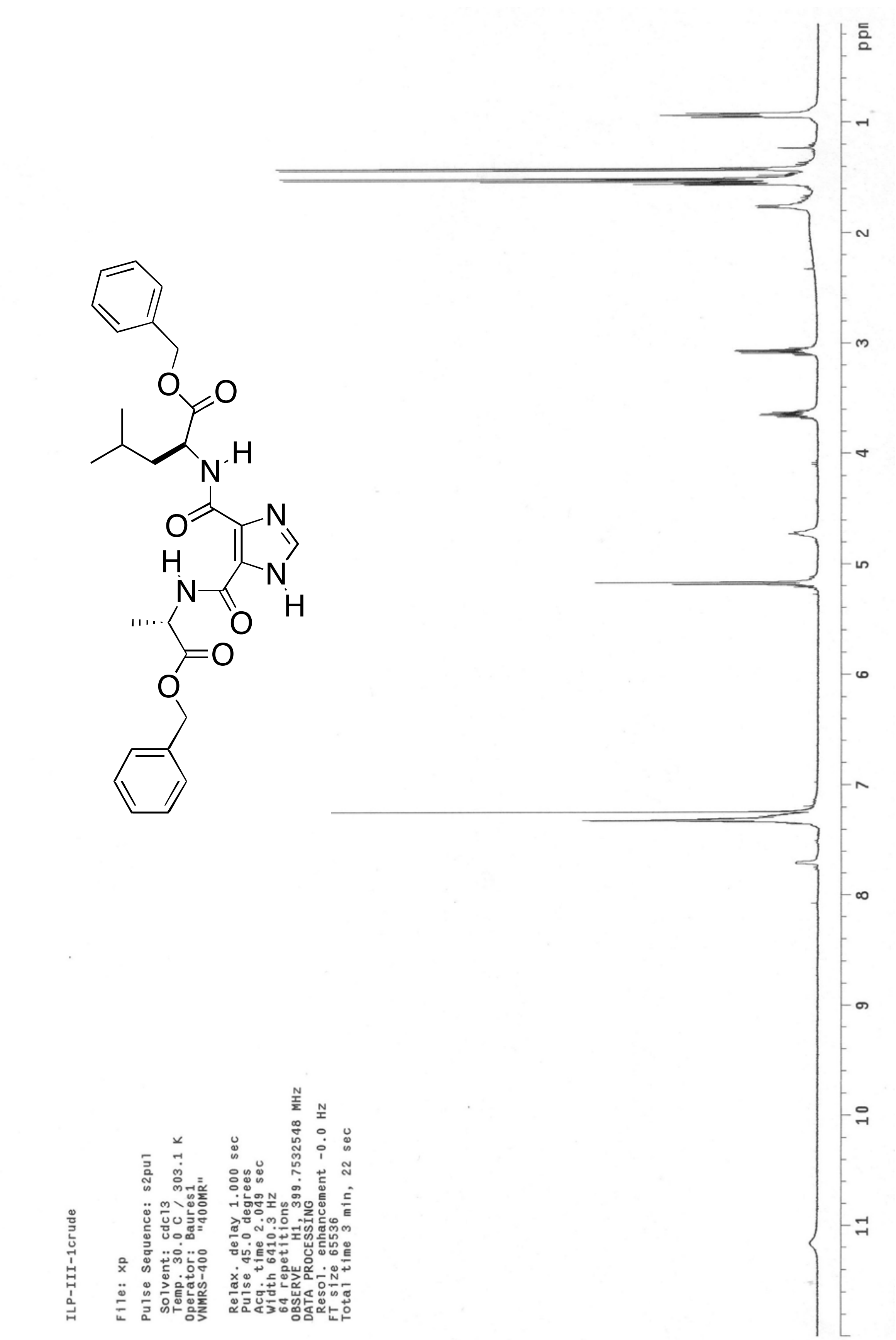

Figure S78. ^{1}H-NMR for the crude reaction to yield **4{**32}.

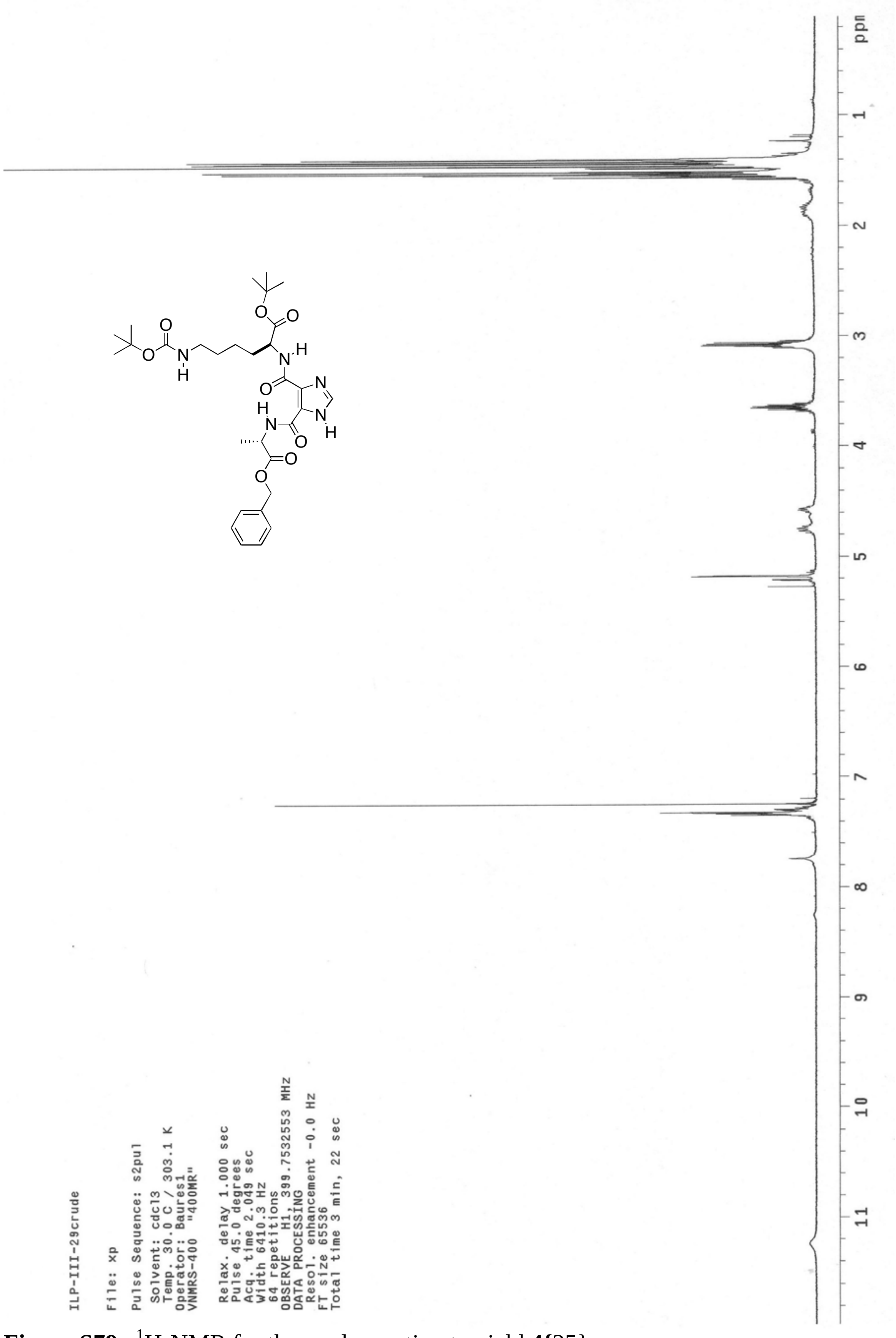

Figure S79. ^{1}H-NMR for the crude reaction to yield **4{**35}.

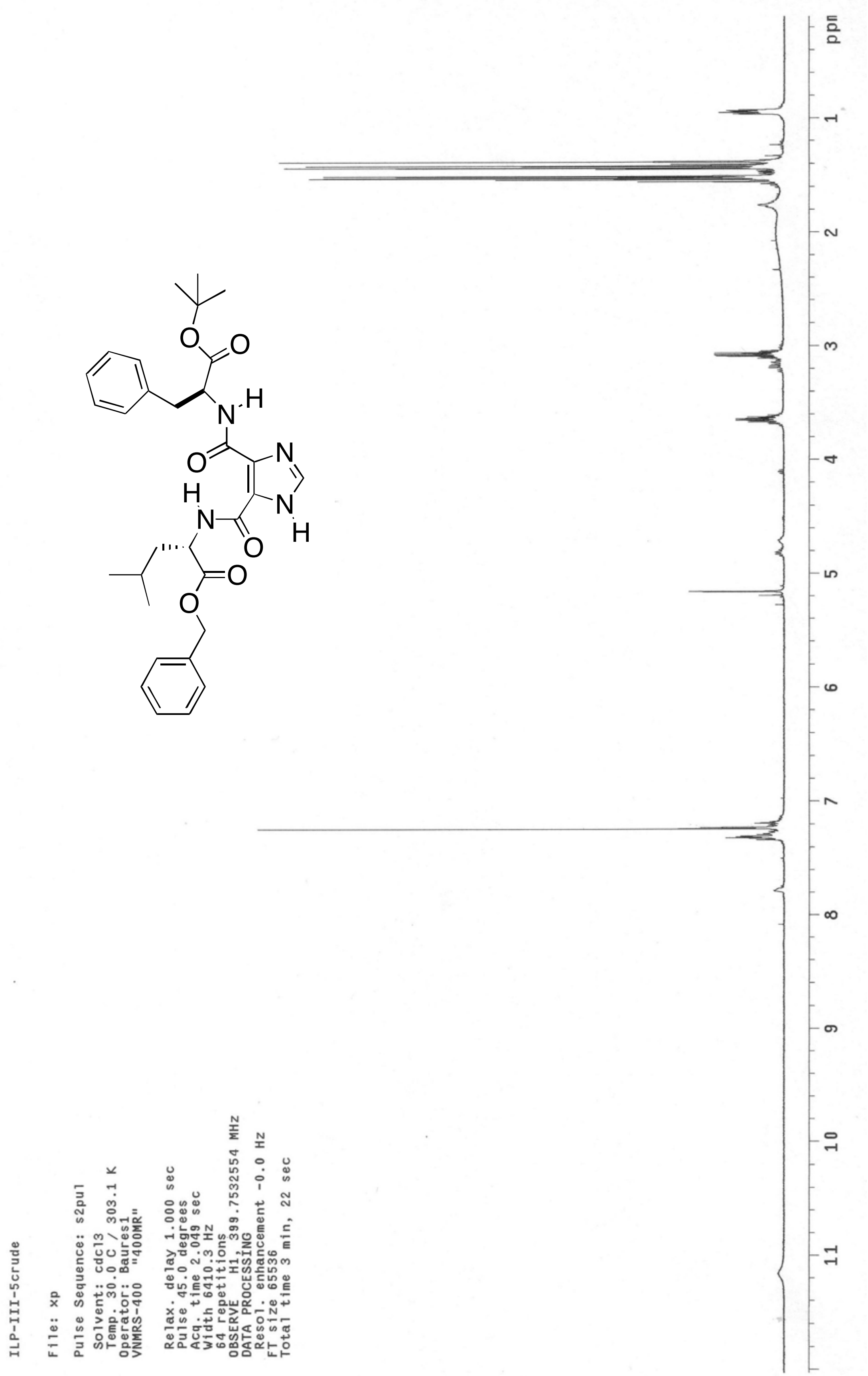

Figure S80. ^{1}H-NMR for the crude reaction to yield **4{**40}.
